# Mendelian Randomization Combined with Bioinformatics Revealed Specific Allergy-Mediated Protective Mechanisms Against Renal Cell Carcinoma

**DOI:** 10.1007/s43657-025-00229-6

**Published:** 2025-11-03

**Authors:** Zixuan Xing, Hao Lei, Yaohui Jiang, Shaobo Wu, Qijuan Zang, Sikai Qiu, Enrui Xie, Yuan Wang, Ning Gao, Yee Hui Yeo, Fanpu Ji, Zhengxiao Li

**Affiliations:** 1 Department of Hepatology, The Second Affiliated Hospital of Xi’an Jiaotong University, 157 Xi Wu Road, Xi’an, 710004 China; 2 Department of Dermatology, the First Affiliated Hospital of Xi’an Jiaotong University, Xi’an, 710061 China; 3 Department of Medicine, Xi’an Jiaotong University, Xi’an, 710061 China; 4 Department of Infectious Diseases, The First Affiliated Hospital of Xi’an Jiaotong University, Xi’an, 710061 China; 5 Karsh Division of Gastroenterology and Hepatology, Cedars-Sinai Medical Center, 8700 Beverly Blvd, Los Angeles, CA 90048 USA; 6 National & Local Joint Engineering Research Center of Biodiagnosis and Biotherapy, The Second Affiliated Hospital of Xi’an Jiaotong University, Xi’an, 710004 China; 7 Key Laboratory of Surgical Critical Care and Life Support (Xi’an Jiaotong University), Ministry of Education, Xi’an, 710000 China; 8 Department of Dermatology, Second Affiliated Hospital of Xi’an Jiaotong University, No. 157 Xi Wu Road, Xi’an, 710004 Shaanxi Province People’s Republic of China

**Keywords:** Allergy, Immune microenvironment, Mendelian randomization, Quantitative trait loci, Renal cell carcinoma

## Abstract

There is evidence that allergic diseases are associated with carcinogenesis. According to translational and epidemiological data, it appears that different cancer types yield different associations. We investigated the relationship between allergic diseases and 28 cancers by Mendelian randomization. Quantitative trait locus analysis was utilized to determine genes expressed in kidney tissue that were affected by allergy-related loci. We further explored the underlying molecular mechanism between allergic diseases and renal cell carcinoma (RCC) with bioinformatics. Of the 28 cancers, evidence suggested that allergies specifically suppressed kidney cancer. Seventy single nucleotide polymorphisms associated with allergic diseases affected the expression of 134 genes in kidney tissue. These 134 genes were enriched in immune-related pathways represented by the major histocompatibility complex class II antigen presentation pathway. Among them, seven core genes were significantly positively correlated with T helper 2 cells. Allergic diseases specifically suppressed RCC through multiple immune pathways. Among them, the major histocompatibility complex class II antigen presentation pathway and T helper 2 cells were the most critical. Our study sheds light on the underlying mechanisms of allergic diseases and RCC and provides therapeutic targets for RCC.

**Supplementary Information:**

The online version contains supplementary material available at 10.1007/s43657-025-00229-6.

## Introduction

With 431,288 new cases and 179,368 annual deaths reported in the 2020 GLOBOCAN statistics, the incidence and mortality of renal cell carcinoma (RCC) are continually increasing (Sung et al. 2021). The pathological types of RCC include clear cell RCC, papillary RCC, chromophobe cell carcinoma, and other rare types, among which clear cell RCC is the most common pathological type, accounting for about 75% of all cases (Hakimi et al. 2016). Although targeted therapy and immunotherapy have significantly improved the prognosis of RCC, drug resistance is still a serious challenge in the clinical treatment of RCC (Alsaab et al. 2018). A considerable proportion of patients with RCC metastasis exhibit primary drug resistance to molecularly targeted drugs. As treatment continues, many patients who were initially sensitive to treatment experience secondary drug resistance about eight years after treatment initiation, which greatly reduces the efficacy of molecularly targeted drugs (Kotecha et al. 2019). Therefore, a better understanding of the exact mechanisms of RCC pathogenesis and progression is imperative to develop clinically feasible strategies to improve prognosis.

Allergic diseases are a group of multiorgan, multisystem syndromes. The prevalence of allergic diseases has steadily increased over the past few decades, affecting up to 30% of the global population (Chinratanapisit et al. 2019; Morales et al. 2019; Pawankar et al. 2011). The diseases caused by type I allergies are the most common and include asthma, allergic rhinitis, eczema, etc. These diseases are essentially immune hyperreactions caused by the participation of various immune factors and immune cells mediated by immunoglobulin E (IgE). Allergic diseases most commonly involve the skin, respiratory tract, and digestive tract because they are rich in cells that bind to IgE.

Immunological homeostasis imbalance is the common pathogenic basis of cancer and allergic diseases. In general, allergic diseases and tumors represent two distinct states of immune function. When the body's immunity is too strong or immune tolerance is impaired, it may lead to allergic diseases. When immunity is low or the immune tolerance is excessive, it may lead to the occurrence of tumors (Carneiro et al. 2021). At present, the relationship between allergic diseases and tumors is still unclear. Epidemiological studies have shown a negative correlation between allergic diseases and tumors (Crawford et al. 2018). Studies of human IgE and cancer risk have shown a clear inverse relationship between serum IgE and the overall cancer risk (Sherman et al. 2008; Van Hemelrijck et al. 2010). In contrast, researchers hypothesize that allergies can increase the risk of tumors. Studies have shown that atopic dermatitis and asthma are risk factors for the development of skin cancer and lung cancer, respectively (Rittmeyer and Lorentz 2012). However, most of these studies are retrospective studies with limited sample sizes and various degrees of bias. In short, the relationship between allergic diseases and tumors and the related mechanisms urgently needs further clarification.

A genome-wide association study (GWAS) will identify sequence variations in the entire human genome and determine single nucleotide polymorphisms (SNPs) associated with the disease. GWAS greatly promotes research on complex diseases. However, many SNP sites identified by GWAS are located in non-coding regions. Due to the existence of linkage disequilibrium, it is impossible to pinpoint the candidate genes causing the disease and decipher the underlying biological processes of genetic effects (Zhao et al. 2020). The position of genes controlling quantitative traits in the genome is called the quantitative trait locus (QTL). It can locate genes controlling quantitative traits in the genome to better understand the genetic mechanism of quantitative traits (Montgomery and Dermitzakis 2011). In this study, we combined Mendelian randomization (MR) and bioinformatics methods to explore the relationship and mechanism of action between allergic diseases and RCC. These findings will provide new insights for the development of clinical therapies and the prevention of diseases.

## Materials and Methods

**Fig. 1:**
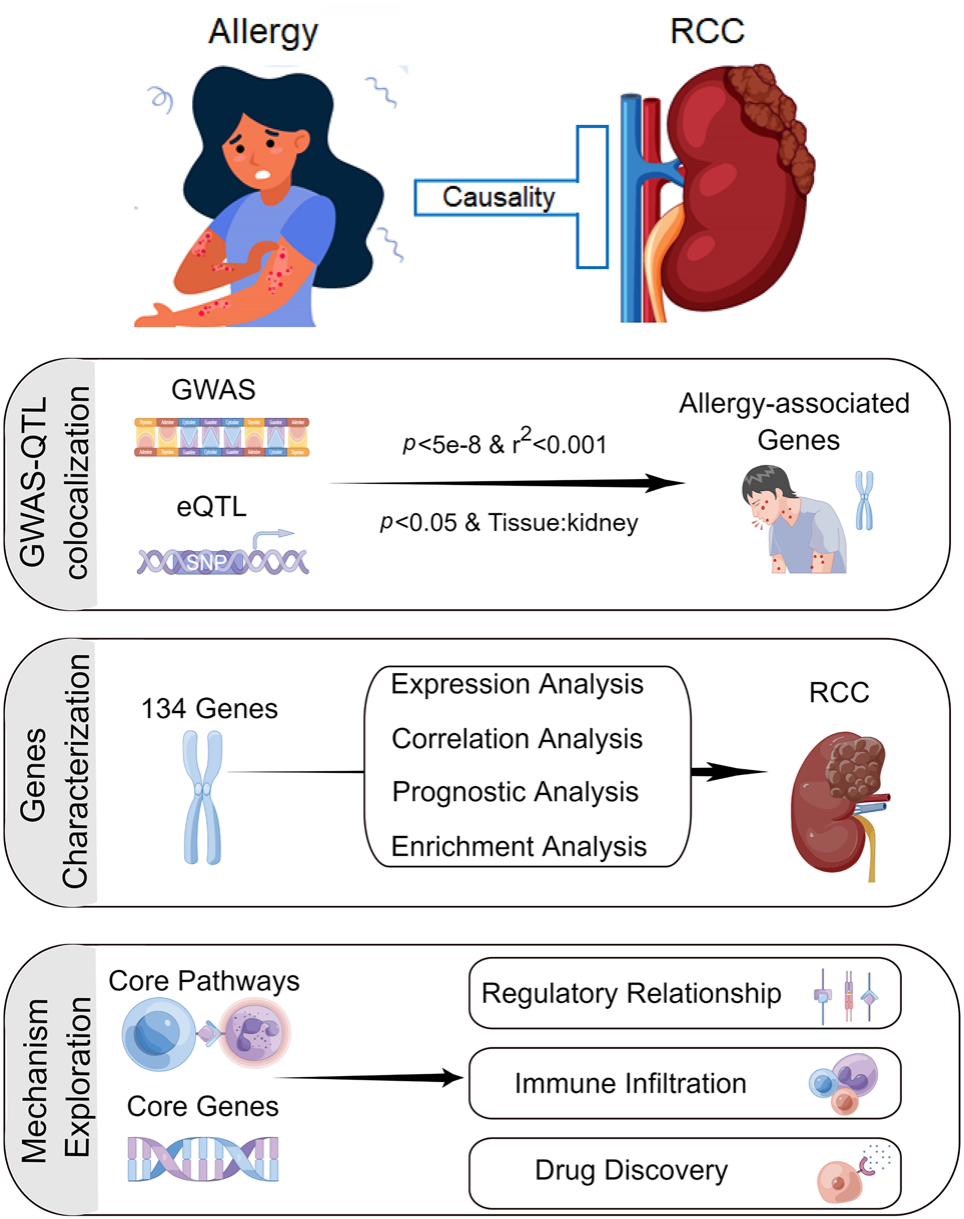
Schematic of the study design and flowchart of analyses performed in this study

The overall study design is shown in Fig. 1. Details of study participants and methods are provided below.

### GWAS Data Sources

We used the summary statistics data from the GWAS of allergic diseases (asthma, hay fever, or eczema) by Ferreira et al. (2017). This analysis included 180,129 cases reporting asthma and/or hay fever and/or eczema as well as 180,709 controls without any of these diseases. We picked 28 cancer types from the FinnGen Consortium based on the types of tumors in The Cancer Genome Atlas (TCGA) to explore possible molecular mechanisms. The summary statistics for 28 cancers were derived from the FinnGen Consortium (https://www.finngen.fi/en). Table S1 gives a summary of data sources for different traits, including GWAS ID, number of cases, number of controls, the number of SNPs, sample size, etc. All GWAS data used in this study were of European ancestry.

### MR analysis

We extracted SNPs (*p* < 5 × 10^–8^) related to allergic diseases (ebi-a-GCST005038) from IEU OpenGWAS using R software. SNP independence was confirmed by a linkage disequilibrium test (r^2^ < 0.001). Then, we calculated R^2^ and F-statistics for each SNP and excluded SNPs with F-statistics less than 10. We extracted data for SNPs in the outcome and removed SNPs associated with the outcome (*p* < 5 × 10^–8^). We also harmonized the exposure and outcome data to ensure that exposure and outcome data were consistent.

We used five methods to estimate the relationship between allergic diseases and various cancers to obtain robust results. Among them, the inverse-variance weighted (IVW) method was used as the primary MR method (Lee et al. 2016). MR Egger intercept and MR Presso test were used to assess horizontal pleiotropy effects. We also performed a series of sensitivity analyses to test the robustness of causality, including Cochran's Q test, and leave-one-out analysis.

The above statistical analyses were performed by TwoSampleMR (version 0.5.6) in R (version 4.1.1). *p* < 0.05 was considered significant unless otherwise stated.

### QTL analysis

We used the QTLbase2 database (http://www.mulinlab.org/qtlbase) to obtain possible effects of specific SNPs on mRNA expression and epigenetic regulation (Huang et al. 2023) and to obtain possible causal regulatory mechanisms.

### Gene expression analysis

TCGA gene expression data for 22 different types of tumors were downloaded from the TCGA dataset (http://portal.gdc.cancer.gov/) (Blum et al. 2018). We analyzed the mRNA expression of genes obtained by expression QTL (eQTL) analysis in kidney chromophobe (KICH), kidney renal clear cell carcinoma (KIRC), kidney renal papillary cell carcinoma (KIRP), and corresponding normal tissues. We further estimated the correlation of these genes in the three tumor tissues by Pearson coefficients. Visualization was achieved using the R package "pheatmap", "circlize", and "ggpubr".

### Prognosis analysis

Prognostic information, including overall survival, for the three RCCs was also downloaded from the TCGA database. Univariate Cox regression and Kaplan–Meier models were then used to evaluate the prognostic role of genes derived from the eQTL analysis for each cancer. The median gene expression was used to divide the high expression group and the low expression group. All analyses were performed using the R packages "survival" and "survminer".

### Enrichment analysis

Pathway enrichment analyses were performed on several databases (Gene Ontology, Kyoto Encyclopedia of Genes and Genomes, and Reactome). Significant pathways were selected using a statistical threshold criterion of adjusted *p* < 0.05. Enrichment analysis and visualization were achieved through the R packages "org.Hs.eg.db", "enrichplot", "GOplot", and "clusterProfiler".

"CBNplot" (R package) was used to visualize the regulatory relationship of each pathway in the enrichment analysis and to identify the regulatory relationship between genes in the pathways of interest (Sato et al. 2022). The core pathways in regulatory relationships in pair-wise tissues were analyzed using the R package "GSVA".

The R package "Mfuzz" was used to find modules associated with the development of tumor tissue from normal tissue and divided clusters of genes or proteins with similar expression patterns to understand the dynamic patterns of these biological molecules and the link to function (Kumar and M 2007).

### Immune Cell Infiltration Analysis

Through the R package "IOBR", we compared the situation of four types of main genes in three types of RCCs and normal tissues, including the major histocompatibility complex (MHC), effector cells, immune checkpoints, and immunosuppressive cells. The immunophenotype score for each sample was determined by considering these four main types of genes. The correlation analysis and visualization of core genes and immune cells were performed through the R package "Hmisc" and "gplots".

### Drug Discovery

To identify potential drugs targeting core pathways for the treatment of RCC, the Therapeutic Target Database (https://db.idrblab.net/ttd/) was queried for information on molecular targets and target outcomes in clinical trials (Zhou et al. 2022).

### Immunohistochemical Staining

A total of 17 kidney specimens (three patients with KICH; five patients with KIRC; five patients with KIRP; and four renal angiomyolipoma) were analyzed by immunohistochemical staining (IHC). Tissues were fixed with paraformaldehyde, paraffin-embedded, and sliced into four μm thick sections by microtome (RM2235, Leica Microsystems Inc, Wetzlar, Germany). Deparaffinized and rehydrated sections were subjected to immunostaining for the molecules IgE (1:50, sc-51997, Santa Cruz Biotechnology) and HLA-DRB1 (1:2000, ab133578, Abcam). Sections were subjected to heat-mediated antigen retrieval using citrate buffer (pH 6) or Tris–EDTA buffer (pH 9) and then incubated with primary antibodies overnight at 4 °C. Sections were then washed and sequentially incubated with biotinylated antibodies and peroxidase-labeled streptavidin according to the manufacturer's instructions. Sections were visualized using diaminobenzidine chromogen and observed under a light microscope (E100; Nikon Corporation, Tokyo, Japan).

## Results

### Evidence for the Association Between Allergic Diseases and renal Malignancies

**Fig. 2:**
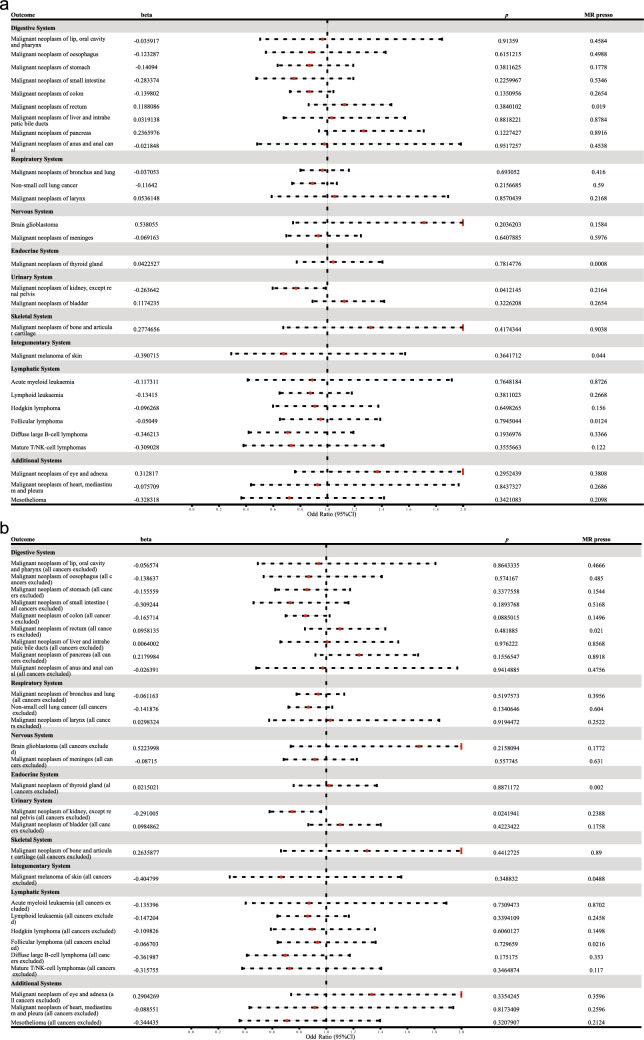
Forest plot of Mendelian randomization (MR) estimates for the association between allergic disease and 28 types of cancer. Allergic disease was significantly associated with a reduced risk of malignant neoplasm of kidney. **a** MR analysis was performed when cancers were not excluded from the control samples. **b** MR analysis was performed when all cancers were excluded from the control samples

After SNP effects were harmonized, 70 SNPs were used to explore the relationship between allergic diseases and cancer. Table S2 shows the screening process for instrumental variables. Table S3**−5** show the specific information of the SNPs in allergic diseases, malignant neoplasms of the kidney except for renal pelvis, and malignant neoplasms of kidney except for renal pelvis (all cancers excluded). In the MR analysis of the IVW approach, genetically-predicted allergic disease was nominally significantly associated with a reduced risk of malignant neoplasm of kidney (odds ratio = 0.77, 95% confidence interval: 0.60–0.99, *p* = 0.04) (Table S6). In contrast, allergic disease was not associated with other types of cancer (Fig. 2, Table S6). When all cancers were excluded from the control samples, consistent results were obtained, i.e., genetically-predicted allergic disease was significantly associated with the risk of malignant kidney cancer only (odds ratio = 0.75, 95% confidence interval: 0.58–0.96, *p* = 0.02) (Fig. 2, Table S7). Based on MR Egger, weighted median, simple mode, and weighted mode methods, the effect values were consistent with the IVW estimates.

Cochran's Q test showed no heterogeneity in MR analysis results for allergic disease and malignant kidney cancer (*p* > 0.05). MR-Egger regression and MR-Presso global test showed no horizontal pleiotropy (*p* > 0.05) for MR analysis results (Table S6 and S7). The scatter plots of the MR analysis and the results of the leave-one-out method are shown in Additional files 1 and 2.

### QTL Analysis Identified Allergy-Associated Genes in Kidney Tissue

We inspected the eQTL and methylation QTL for the 70 SNPs using the QTLbase database. After screening for *p* < 0.05, we found that the 70 SNPs affected the expression of 134 genes (Table S8) and the methylation of 17 genes in kidney tissue (Fig. S1a, Table S9). Most of the SNPs did not affect gene methylation in kidney tissue. Therefore, we conducted a follow-up analysis regarding the results obtained by the eQTL analysis.

### Characterization of 134 Genes in RCC

We applied hierarchical clustering to the 134 genes in three different types of RCC (Fig. S1**b**). Then the cluster analysis of 134 genes was performed on the three RCCs and the corresponding normal samples (Fig. S1c–e). By performing correlation analysis in tumor samples, most of the 134 genes were positively correlated [76.3% (5819/7626) of the relationships between genes in KICH; 69.5% (5390/7750) in KIRC; and 71.3% (5438/7626) in KIRP] (Fig. S1f–h). The correlation was analyzed by Pearson's correlation coefficient. Univariate analysis showed that 40 out of 122 genes in KICH, 68 out of 125 genes in KIRC, and 32 out of 124 genes in KIRP were significantly associated with overall survival (Fig. S2). We further performed survival analysis to confirm that genes, including *HLA-DRB1*, *HLA-DQB1*, and *HLA-DOB*, had a significant impact on survival (Fig. S2, Table S10).

### Core Pathways of Allergy-Mediated Protective Mechanisms Against RCC

**Fig. 3:**
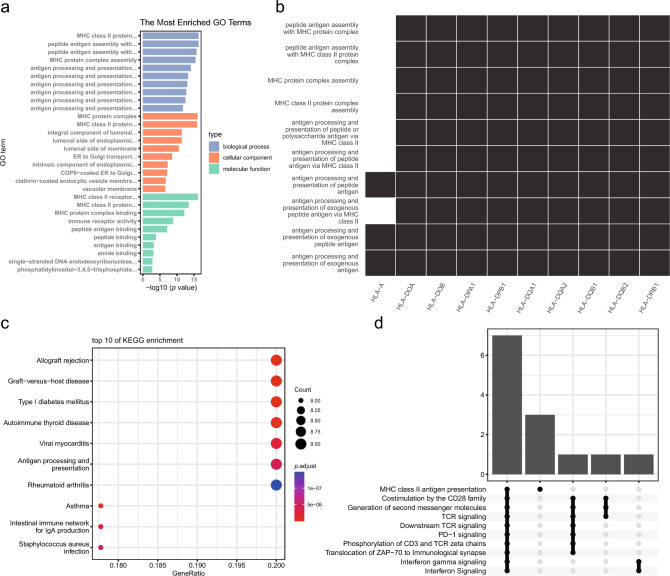
Significant pathway results from enrichment analysis of the 134 allergy-associated genes. The antigen presentation pathway was commonly enriched in the three databases. **a** Barplot for Gene Ontology enrichment analysis. **b** Heatmap for Gene Ontology enrichment analysis. The row indicates the enriched pathway, the column indicates the input gene, and the black squares indicate the pathway containing that gene. **c** Dotplot for Kyoto Encyclopedia of Genes and Genomes enrichment analysis. **d** Upset plot for Reactome enrichment analysis

To further study the molecular mechanism of the 134 genes in allergy-mediated protection, pathway analysis of three databases, Gene Ontology, Kyoto Encyclopedia of Genes and Genomes, and Reactome, was performed. The enrichment analysis results of the three databases focused on immune-related pathways including the MHC class II antigen presentation pathway, cytokine signaling, and T cell receptor signaling, among which the antigen presentation pathway was commonly enriched (Fig. 3a–d).

The pathways enriched by the Reactome database were further analyzed to obtain the upstream and downstream regulatory relationships between pathways. MHC class II antigen presentation, generation of second messenger molecules, interferon gamma signaling, and translocation of ZAP-70 to the immunological synapse were at the core of the regulatory relationships in KICH, KIRC, and KIRP (Fig. S3a–c). Furthermore, these four pathways were significantly upregulated in KIRC and KIRP but not in KICH from normal tissues (Fig. S3d–g).

### Correlation Between Core Genes and the Tumor Microenvironment

**Fig. 4:**
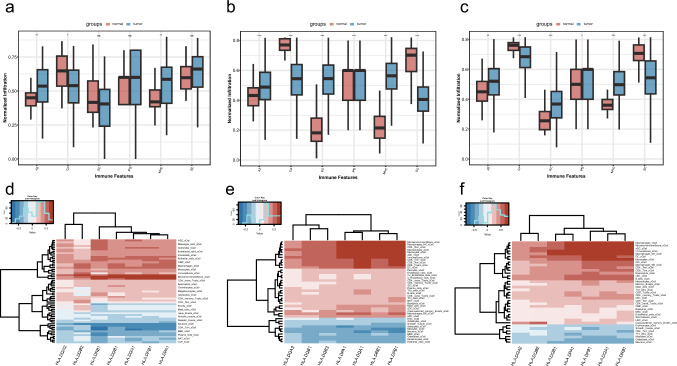
Tumor microenvironment characteristics of the three tumor types. Major histocompatibility complex molecules were significantly upregulated in all three tumor types. Kidney chromophobe had a different immune profile compared to kidney renal clear cell carcinoma and kidney renal papillary cell carcinoma. **a**–**c** Scoring of major histocompatibility complex molecules, effector cells, immune checkpoints, and immunosuppressive cells in kidney chromophobe (**a**), kidney renal clear cell carcinoma (**b**), and kidney renal papillary cell carcinoma (**c**). **d**–**f** The relationship between the seven core genes (*HLA-DQA1*, *HLA-DQA2*, *HLA-DQB1*, *HLA-DQB2*, *HLA-DPA1*, *HLA-DPB1*, and *HLA-DRB1*) and the tumor microenvironment in kidney chromophobe (**d**), kidney renal clear cell carcinoma (**e**), and kidney renal papillary cell carcinoma (**f**)

Using the algorithm to quantify the tumor microenvironment, MHC molecules, effector cells, immune checkpoints, and immunosuppressive cells were significantly correlated with KIRC and KIRP (Fig. 4a–c). Among them, MHC molecules were significantly upregulated in all three tumor types.

We further analyzed the molecular regulatory relationships of the four core pathways in the tumor and normal tissues. Gene clusters involved in the process of progressing normal tissue to RCC were obtained through time series analysis (Fig. S4). Seven regulatory genes shared by the four pathways were all enriched in the same cluster in the time series analysis (Table S11).

The relationship between the seven core genes (*HLA-DQA1*, *HLA-DQA2*, *HLA-DQB1*, *HLA-DQB2*, *HLA-DPA1*, *HLA-DPB1*, and *HLA-DRB1*) and the tumor microenvironment was analyzed. The seven genes were positively correlated with T helper 2 (Th2) cells, dendritic cells, and macrophages in KIRC and KIRP, whereas the results for KICH were different from the other two tumor types (Fig. 4d-f).

### Approved or Clinical-Phase Drug Candidates for the Core Pathways

To repurpose known drug compounds, we used the Therapeutic Target Database to find drugs that target the four core pathways. Approved or clinical-phase drug candidates were obtained through further screening of drugs that could target the four pathways (**Table S12**). Among them, the approved drug glatiramer acetate (GA) targets one of the core genes, *HLA-DRB1*. The candidate drugs have the potential to treat RCC or provide a reference for new drug development based on the protective mechanisms of allergic diseases on RCC.

### IgE and HLA-DRB1 Expression in Kidney Tissue Samples

**Fig. 5:**
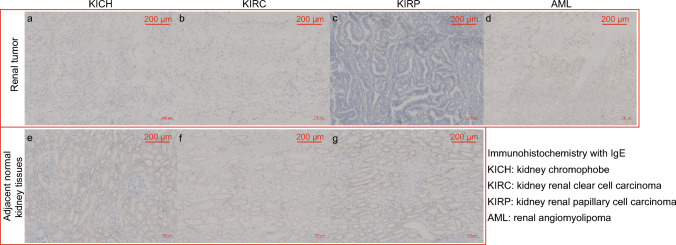
Immunohistochemical analysis of the expression of immunoglobulin E in kidney sections. Immunoglobulin E was expressed in renal angiomyolipoma and normal kidney tissues but not in the three types of renal cancer tissues. **a** Kidney chromophobe; **b** Kidney renal clear cell carcinoma; **c** Kidney renal papillary cell carcinoma; **d** Renal angiomyolipoma; Adjacent normal kidney tissues from kidney chromophobe (**e**), kidney renal clear cell carcinoma (**f**), and kidney renal papillary cell carcinoma (**g**)

**Fig. 6:**
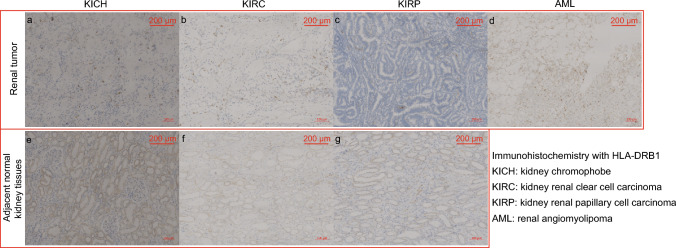
Immunohistochemical analysis of the expression of HLA-DRB1 in kidney sections. HLA-DRB1 was expressed in renal angiomyolipoma and normal kidney tissues but not in the three types of renal cancer tissues. **a** Kidney chromophobe; **b** Kidney renal clear cell carcinoma; **c** Kidney renal papillary cell carcinoma; **d** Renal angiomyolipoma; Adjacent normal kidney tissues from kidney chromophobe (**e**), kidney renal clear cell carcinoma (**f**), and kidney renal papillary cell carcinoma (**g**)

Kidney specimens from renal angiomyolipoma and tumor adjacent tissues were classified as the control group. IHC staining showed that IgE (Fig. 5) and HLA-DRB1 (Fig. 6) were expressed in renal angiomyolipoma and normal kidney tissues but not in the three types of renal cancer tissues. In addition, IgE and HLA-DRB1 were primarily expressed in the renal tubules of normal renal tissue and not in the glomerulus.

## Discussion

MR is an important method to study the genetic basis of complex diseases and can identify genetic risk factors and genomic variations of complex diseases. Using this information, we are able to identify new targets and drugs for the treatment of complex diseases. IVW analysis revealed that allergic diseases, such as allergic rhinitis or hay fever (Table S13), specifically reduced the risk of renal malignancy. The MR and QTL analysis identified 134 related genes. The immune-related pathways represented by MHC class II antigen presentation were highly relevant in allergic disease-mediated RCC suppression. In addition, seven core genes in the HLA class II family mediated the protective mechanism by regulating Th2 cells, macrophages, dendritic cells, and other immune cells in the immune microenvironment (Fig. S5). IgE and *HLA-DRB1* may be potential biomarkers for kidney cancer.

AllergoOncology is an emerging discipline that studies the relationship between allergic diseases and tumors. The field is committed to further improving the clinical treatment efficacy of tumors (Jensen-Jarolim et al. 2017). Previous epidemiological studies have shown that the relationship between allergic diseases and tumors is multifactorial. It is hypothesized that chronic stimulation of the immune system will cause random cancer-promoting mutations in actively dividing cells (Simpson et al. 2002). The immune surveillance hypothesis, as well as the prevention hypothesis, explain allergic diseases as protective factors against tumors. The immune surveillance hypothesis states that increased immune surveillance following a hyperreactive immune response can hinder cancer development. The prevention hypothesis states that allergic diseases can prevent cancer by removing potential carcinogens (Rittmeyer and Lorentz 2012; Söderberg et al. 2004).

Previous studies have revealed that eosinophils (Simon et al. 2019), group 2 innate lymphoid cells (Wu et al. 2022), IgE (Luiten et al. 1996; Schmidt et al. 1976; Vecchi and Lissoni 1995), and signal transducer and activator of transcription 6 (Karpathiou et al. 2021) were associated with both allergic reactions and tumors. These factors may be involved in the mechanism of the allergy-tumor link. However, there have been contradictory findings, particularly when previous studies have focused on a single cancer type (Kim et al. 2023; Yu et al. 2022) without an in-depth exploration of the relationship between allergic diseases and cancers, especially renal cancers, in terms of causality and the potential mechanisms. In this study, we demonstrated the relationship between allergic diseases and renal cancer and explored potential mechanisms and drugs.

IgE plays an important role in tumor suppression in allergic diseases. IgE targeting tumor antigens can play a direct role by recognizing target antigens, interfering with signal transduction, inhibiting tumor growth, etc. (Jensen-Jarolim et al. 2017). IgE can also produce effector functions against tumor cells by binding to specific effector cell receptors (Pellizzari et al. 2019). The relationship and mechanism between allergic diseases and tumors is still controversial. Environmental factors, such as allergen exposure, pollution, and lifestyle behaviors, may confound the association between allergic diseases and RCC. Individuals with allergies may adopt healthier lifestyles or have different environmental exposures than individuals without allergies. Individuals with allergic diseases may undergo more frequent medical evaluations or have a higher likelihood of receiving certain medications that could influence RCC risk or progression.

The majority of the previous studies examining the relationship between allergic diseases and tumors were observational studies, and the results were subject to various confounding factors. In this study, the relationship between allergic diseases and tumors was studied at the genome and transcriptome level using methods such as MR. Therefore, we avoided the confounding factors as much as possible. However, allergic diseases such as asthma and allergic rhinitis typically manifest in childhood or early adulthood, whereas RCC incidence increases with age, peaking in the sixth to eighth decades of life. Age-related changes in immune function may confound the association between allergic diseases and RCC risk. Long-term cohort studies tracking individuals with allergic diseases from childhood to adulthood and assessing their RCC risk over time will provide valuable insights into the causal relationship and potential mechanisms underlying the observed association.

This study found that allergic diseases may inhibit RCC through MHC class II antigen presentation, interferon gamma signaling, generation of secondary messenger molecules, and translocation of ZAP-70 to immunological synapses (the four core pathways). The MHC class II antigen presentation pathway is involved in the activation of CD4^+^ T cells, thereby regulating tumor immunity. Studies have shown that CD4^+^ T cells mediate the continuous activation of cytotoxic T lymphocytes, which enhances the response to immunotherapy. CD8^+^ T cells also require CD4^+^ T cells to function (Haabeth et al. 2014; Martin-Orozco et al. 2009). Research focusing on the MHC class II antigen presentation pathway is a new direction for enhancing tumor immunotherapy and tumor vaccines. The interferon gamma signaling pathway coordinates the balance of protumor and antitumor immunity in the tumor microenvironment. In addition, interferon gamma can induce MHC class II expression through the canonical Janus kinase/signal transducer and activator of transcription pathway (Forero et al. 2016).

We identified seven core genes shared by the four pathways. The seven genes are all MHC class II molecules. MHC class II molecules participate in the processing and presentation of foreign antigens to CD4^+^ T cells, mediate the activation of CD4^+^ T cells, and participate in the regulation of the tumor immune microenvironment. CD4^+^ T cells include multiple subtypes such as T helper 1 cells, Th2 cells, and regulatory T cells. Th2 cells may play a key role in allergy-mediated RCC suppression. Mice deficient in Th2 cytokines, interleukin (IL)−4, and IL-5 showed reduced tumor clearance, whereas injection of IL-4 enhanced tumor clearance, which was associated with increased infiltration of eosinophils, macrophages, neutrophils, and some lymphocytes (Hung et al. 1998). In addition, tumor growth can be restored by the administration of an IL-5 monoclonal antibody (Modesti et al. 1993). Adoptive cell therapy using Th2 cells has also been shown to be effective in melanoma animal models. Notably, some studies have found that Th2 cells promote tumor growth (Tokumaru et al. 2020). This suggests that the tumor type may dictate the regulation of tumor immunity by Th2 cells.

Interestingly, when analyzing the association of core genes with the tumor microenvironment, we found that KICH was significantly different from KIRC and KIRP. In KICH, there was no significant difference in immune-related indicators between the tumor tissue and the normal tissue. At the same time, the seven genes in the HLA family were more closely related to cells involved in cell adhesion and extracellular matrix remodeling, while CD4^+^ T cells or CD8^+^ T cells were not highly correlated, which is consistent with previous findings (Lu et al. 2020; Zhang et al. 2021). This phenomenon may be related to the differences in the original mutation of each RCC subtype, but further research is still needed.

Furthermore, MHC class II molecules and associated core genes could serve as biomarkers to stratify individuals based on their susceptibility to RCC. Alterations in MHC class II molecule expression or function may indicate a predisposition to RCC or a potential protective effect conferred by allergic diseases. Monitoring the levels of CD4 + T cells, particularly Th2 cells, in peripheral blood or the tumor microenvironment are additional potential biomarkers for kidney cancer risk. Additional studies are needed to confirm that imbalances in the Th2 cell population or a dysfunctional Th2 cell response leads to an increased risk of RCC development. Moreover, identifying specific SNPs or genetic loci linked to both allergic diseases and RCC risk will enable the development of genetic risk scores to stratify individuals based on their susceptibility to RCC.

MR and bioinformatics analyses rely on statistical associations and computational predictions that can suggest but not confirm relationships. The causal relationships between the identified genetic variants, gene expression levels, and RCC risk require further studies for confirmation. Locally sustained tumor immunity involves complex interactions of immune cells, cytokines, and regulatory molecules in the tumor microenvironment. Identifying the precise mechanism by which local sustained tumor immunity mediates the upregulation of MHC class II molecules on local antigen-presenting cells was beyond the scope of this study. The biological mechanisms underlying the association between allergic diseases and RCC are multifaceted. Disentangling the complex interplay of genetic, environmental, and immunological factors requires the integration of genomics, immunology, epidemiology, and bioinformatics. Future studies could develop transgenic or knockout mouse models to study the in vivo effects of the identified genes on RCC progression and immune response. Studies utilizing plasmids or viral vectors to overexpress specific genes in RCC cell lines and assessing the impact on cell proliferation, apoptosis, and immune response should be conducted.

We explored drugs that target the key pathways identified in this study. GA is a cationic polypeptide approved for the treatment of relapsing–remitting multiple sclerosis. The mechanism of action of GA in the treatment of multiple sclerosis is unknown. However, it may interact with *HLA-DRB1* to antagonize the activation of myelin basic protein-specific T cell clones (Prod'homme and Zamvil 2019). In recent years, GA has attracted attention in the tumor therapy field because of its cationic polypeptide properties. A study demonstrated that GA enhanced the cytotoxicity of natural killer cells to target tumor cells (Maghazachi et al. 2016). Furthermore, tumors most responsive to anti-PD-1 combined with GA therapy showed increased CD4^+^ T cell markers and natural killer cell infiltration (Huang et al. 2022). An intratumoral delivery modality combined with an immunostimulant may enhance the local immune response while attenuating systemic immune-related adverse events (Pressnall et al. 2021). In addition, other targets of the pathway and the identified SNP sites provide meaningful future research directions for the treatment of RCC.

Our research provided further evidence to understand the mechanism of action between allergic diseases and RCC, which may have new implications for the prevention of RCC and the development of more effective treatment methods. This study also revealed that local persistent tumor immunity could be mediated by specifically stimulating the upregulation of MHC class II molecules on local antigen-presenting cells. Furthermore, this study demonstrated the potential for early prevention of RCC by inducing local allergies in those at high risk of RCC or RCC recurrence. Finally, we hypothesize that it is possible to reduce the burden on medical resources in RCC screening by focusing on patients without allergic diseases.

The current study has several limitations. First, the MR analysis only included study participants of European ethnicity, and our findings may not be generalizable to other ethnicities. Future research should include diverse racial or ethnic groups, socioeconomic backgrounds, and geographic regions to ensure the applicability across diverse populations and healthcare settings. Second, the sample size of this study was small, and there may be individual extreme values or deviations that affect the overall results. Further validation of the significance of our findings in larger, more diverse groups is needed to increase the generalizability and credibility of the results. Finally, we performed the analysis without accounting for multiple testing burden or other corrections and used a loose p-value cutoff when querying genes associated with the 70 SNPs used as genetic tools, which would have increased the probability of false positive results, making the results less reliable.

## Conclusion

Allergic diseases have the potential for specific inhibitory effects on RCC, which may be achieved through a variety of immune pathways. The MHC II antigen presentation pathway is the most critical. CD4^+^ T cells, especially Th2 cells, have an inhibitory effect on RCC. This study revealed new insights on the mechanism related to allergic diseases and RCC and provided potential future research directions for the prevention and treatment of RCC.

## Supplementary Information

Below is the link to the electronic supplementary material.

Supplementary file1 (PDF 1144 KB)

Supplementary file2 (PDF 1083 KB)

Supplementary file3 (XLSX 9583 KB)

## Data Availability

The datasets analysed during the current study are available in the IEU OpenGWAS and TCGA repository, https://gwas.mrcieu.ac.uk/ & https://portal.gdc.cancer.gov/.

## Abbreviations

GWAS: Genome-wide association study
QTL: Quantitative trait locus
RCC: Renal cell carcinoma
SNP: Single nucleotide polymorphism
MHC: Major histocompatibility complex
ccRCC: Clear cell renal cell carcinoma
PRCC: Papillary renal cell carcinoma
ChRCC: Chromophobe cell carcinoma
IgE: Immunoglobulin E
LD: Linkage disequilibrium
IVW: Inverse-variance weighted
MR: Mendelian randomization
TCGA: The Cancer Genome Atlas
eQTL: Expression quantitative trait loci
KICH: Kidney chromophobe
KIRC: Kidney renal clear cell carcinoma
KIRP: Kidney renal papillary cell carcinoma
OS: Overall survival
GO: Gene Ontology
KEGG: Kyoto Encyclopedia of Genes and Genomes
EC: Effector cells
CP: Immune checkpoints
SC: Immunsuppressive cells
IPS: Immunophenotype score
TTD: Therapeutic Target Database
OR: Odd ratio
CI: Confidence interval
meQTL: Methylation quantitative trait loci
Th2 cell: T helper 2 cell
DC: Dendritic cells
HLA: Human leukocyte antigen
GA: Glatiramer Acetate
APC: Antigen-presenting cell
