# Supplementary material for: Mendelian Randomization Combined with Bioinformatics Revealed Specific Allergy-Mediated Protective Mechanisms Against Renal Cell Carcinoma
Source: Phenomics. 2025 Nov 3;5(5):577–89. doi: 10.1007/s43657-025-00229-6 (PMC12881226; doi:10.1007/s43657-025-00229-6)
Supplement: Supplementary file 1 — Supplementary file1 (PDF 1144 KB) [file 43657_2025_229_MOESM1_ESM.pdf]

Additional file 1

Figure S1: Scatter plot of the causal effect of allergic disease on Acute myeloid leukaemia (all cancers excluded)

Figure S2: Scatter plot of the causal effect of allergic disease on Acute myeloid leukaemia

Figure S3: Scatter plot of the causal effect of allergic disease on Brain glioblastoma (all cancers excluded)

Figure S4: Scatter plot of the causal effect of allergic disease on Brain glioblastoma

Figure S5: Scatter plot of the causal effect of allergic disease on Diffuse large B-cell lymphoma (all cancers excluded)

Figure S6: Scatter plot of the causal effect of allergic disease on Diffuse large B-cell lymphoma

Figure S7: Scatter plot of the causal effect of allergic disease on Follicular lymphoma (all cancers excluded)

Figure S8: Scatter plot of the causal effect of allergic disease on Follicular lymphoma

Figure S9: Scatter plot of the causal effect of allergic disease on Hodgkin lymphoma (all cancers excluded)

Figure S10: Scatter plot of the causal effect of allergic disease on Hodgkin lymphoma

Figure S11: Scatter plot of the causal effect of allergic disease on Lymphoid leukaemia (all cancers excluded)

Figure S12: Scatter plot of the causal effect of allergic disease on Lymphoid leukaemia

Figure S13: Scatter plot of the causal effect of allergic disease on Malignant melanoma of skin (all cancers excluded)

Figure S14: Scatter plot of the causal effect of allergic disease on Malignant melanoma of skin

Figure S15: Scatter plot of the causal effect of allergic disease on Malignant neoplasm of anus and anal canal (all cancers excluded)

Figure S16: Scatter plot of the causal effect of allergic disease on Malignant neoplasm of anus and anal canal

Figure S17: Scatter plot of the causal effect of allergic disease on Malignant neoplasm of bladder (all cancers excluded)

Figure S18: Scatter plot of the causal effect of allergic disease on Malignant neoplasm of bladder

Figure S19: Scatter plot of the causal effect of allergic disease on Malignant neoplasm of bone and articular cartilage (all cancers excluded)

Figure S20: Scatter plot of the causal effect of allergic disease on Malignant neoplasm of bone and articular cartilage

Figure S21: Scatter plot of the causal effect of allergic disease on Malignant neoplasm of bronchus and lung (all cancers excluded)

Figure S22: Scatter plot of the causal effect of allergic disease on Malignant neoplasm of bronchus and lung

Figure S23: Scatter plot of the causal effect of allergic disease on Malignant neoplasm of colon (all cancers excluded)

Figure S24: Scatter plot of the causal effect of allergic disease on Malignant neoplasm of colon

Figure S25: Scatter plot of the causal effect of allergic disease on Malignant neoplasm of eye and adnexa (all cancers excluded)

Figure S26: Scatter plot of the causal effect of allergic disease on Malignant neoplasm of eye and adnexa

Figure S27: Scatter plot of the causal effect of allergic disease on Malignant neoplasm of heart, mediastinum and pleura (all cancers excluded)
Figure S28: Scatter plot of the causal effect of allergic disease on Malignant neoplasm of heart, mediastinum and pleura
Figure S29: Scatter plot of the causal effect of allergic disease on Malignant neoplasm of kidney, except renal pelvis (all cancers excluded)
Figure S30: Scatter plot of the causal effect of allergic disease on Malignant neoplasm of kidney, except renal pelvis
Figure S31: Scatter plot of the causal effect of allergic disease on Malignant neoplasm of larynx (all cancers excluded)
Figure S32: Scatter plot of the causal effect of allergic disease on Malignant neoplasm of larynx
Figure S33: Scatter plot of the causal effect of allergic disease on Malignant neoplasm of lip, oral cavity and pharynx (all cancers excluded)
Figure S34: Scatter plot of the causal effect of allergic disease on Malignant neoplasm of lip, oral cavity and pharynx
Figure S35: Scatter plot of the causal effect of allergic disease on Malignant neoplasm of liver and intrahepatic bile ducts (all cancers excluded)
Figure S36: Scatter plot of the causal effect of allergic disease on Malignant neoplasm of liver and intrahepatic bile ducts
Figure S37: Scatter plot of the causal effect of allergic disease on Malignant neoplasm of meninges (all cancers excluded)
Figure S38: Scatter plot of the causal effect of allergic disease on Malignant neoplasm of meninges
Figure S39: Scatter plot of the causal effect of allergic disease on Malignant neoplasm of oesophagus (all cancers excluded)
Figure S40: Scatter plot of the causal effect of allergic disease on Malignant neoplasm of oesophagus
Figure S41: Scatter plot of the causal effect of allergic disease on Malignant neoplasm of pancreas (all cancers excluded)
Figure S42: Scatter plot of the causal effect of allergic disease on Malignant neoplasm of pancreas
Figure S43: Scatter plot of the causal effect of allergic disease on Malignant neoplasm of rectum (all cancers excluded)
Figure S44: Scatter plot of the causal effect of allergic disease on Malignant neoplasm of rectum
Figure S45: Scatter plot of the causal effect of allergic disease on Malignant neoplasm of small intestine (all cancers excluded)
Figure S46: Scatter plot of the causal effect of allergic disease on Malignant neoplasm of small intestine
Figure S47: Scatter plot of the causal effect of allergic disease on Malignant neoplasm of stomach (all cancers excluded)
Figure S48: Scatter plot of the causal effect of allergic disease on Malignant neoplasm of stomach
Figure S49: Scatter plot of the causal effect of allergic disease on Malignant neoplasm of thyroid gland (all cancers excluded)
Figure S50: Scatter plot of the causal effect of allergic disease on Malignant neoplasm of thyroid gland
Figure S51: Scatter plot of the causal effect of allergic disease on Mature T/NK-cell lymphomas (all cancers excluded)

Figure S52: Scatter plot of the causal effect of allergic disease on Mature T/NK-cell lymphomas

Figure S53: Scatter plot of the causal effect of allergic disease on Mesothelioma (all cancers excluded)

Figure S54: Scatter plot of the causal effect of allergic disease on Mesothelioma

Figure S55: Scatter plot of the causal effect of allergic disease on Non-small cell lung cancer (all cancers excluded)

Figure S56: Scatter plot of the causal effect of allergic disease on Non-small cell lung cancer

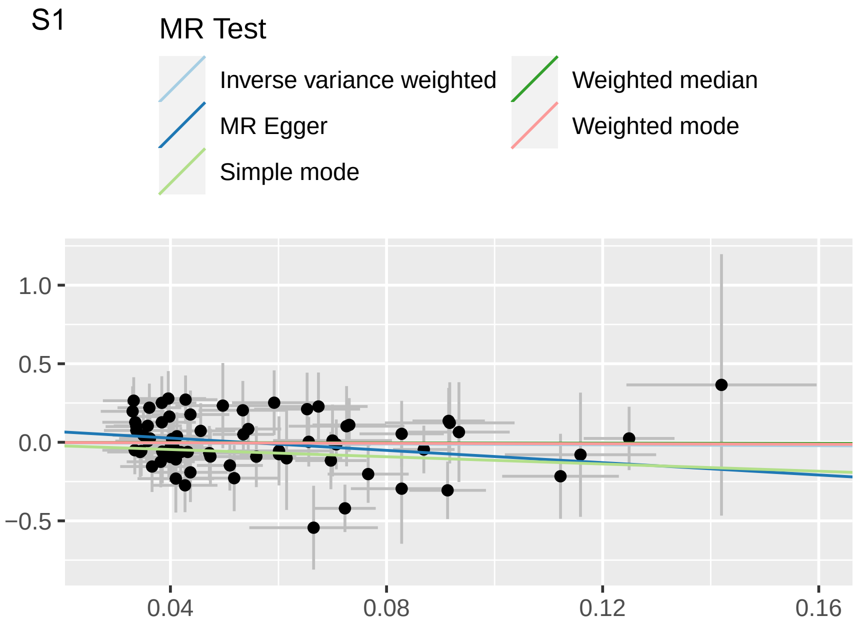
S1
MR Test
Inverse variance weighted
MR Egger
Simple mode
Weighted median
Weighted mode
1.0
0.5
0.0
−0.5
0.04
0.08
0.12
0.16

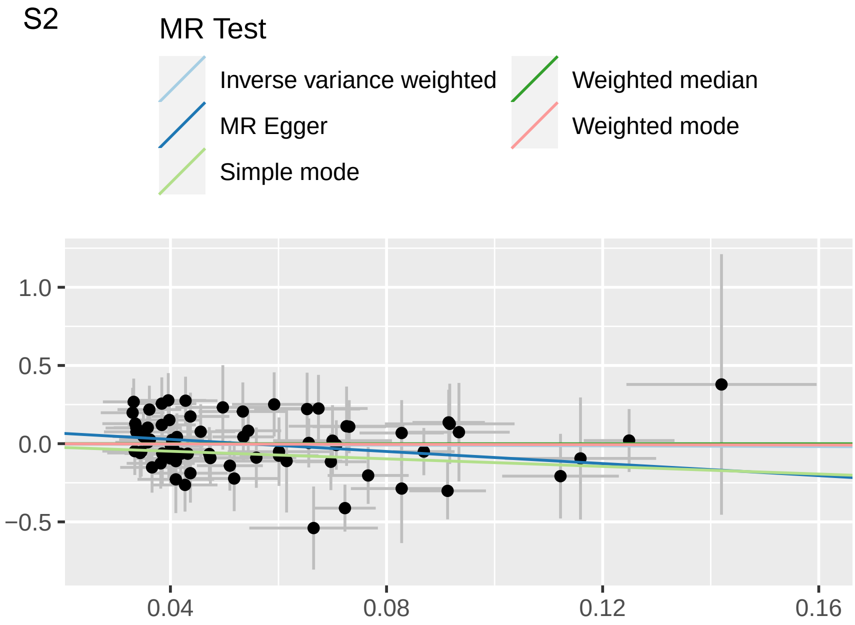
S2
MR Test
Inverse variance weighted
MR Egger
Simple mode
Weighted median
Weighted mode
1.0
0.5
0.0
−0.5
0.04
0.08
0.12
0.16

S3

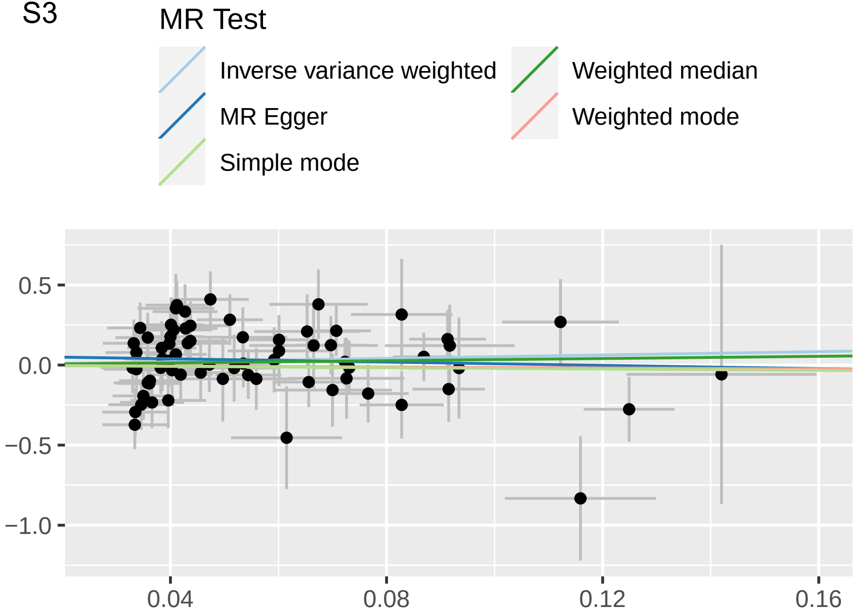
MR Test
Inverse variance weighted
MR Egger
Simple mode
Weighted median
Weighted mode
0.5
0.0
−0.5
−1.0
0.04
0.08
0.12
0.16

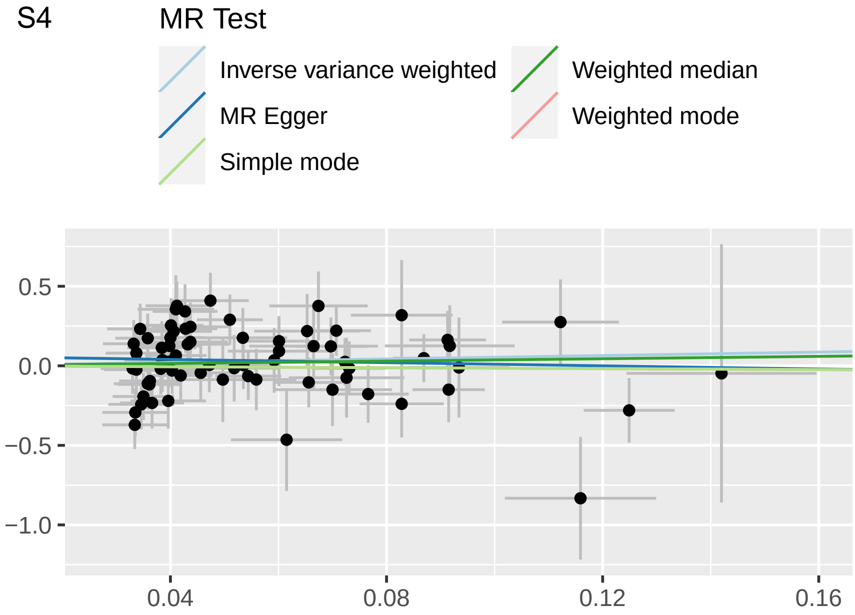
S4
MR Test
Inverse variance weighted
Weighted median
MR Egger
Weighted mode
Simple mode
0.5
0.0
−0.5
−1.0
0.04
0.08
0.12
0.16

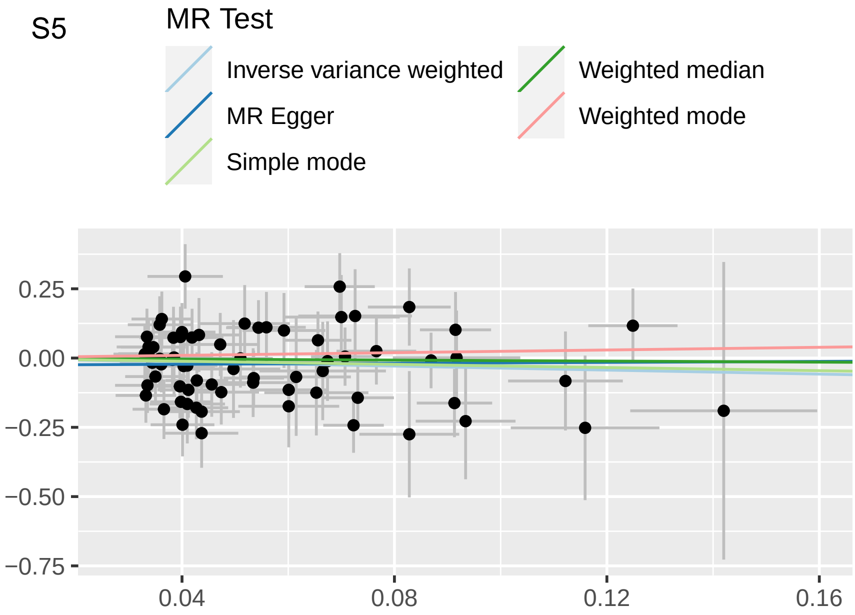
S5
MR Test
Inverse variance weighted
MR Egger
Simple mode
Weighted median
Weighted mode
0.25
0.00
−0.25
−0.50
−0.75
0.04
0.08
0.12
0.16

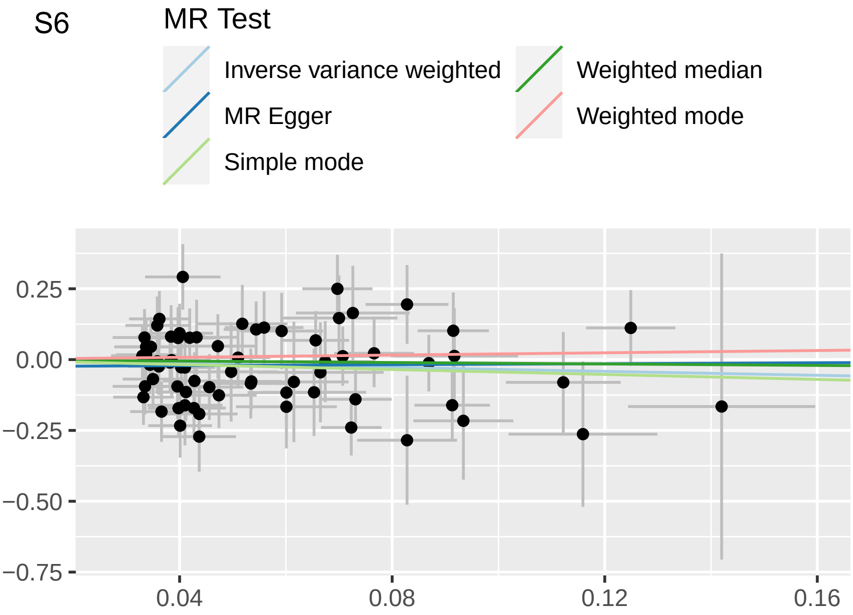
S6
MR Test
Inverse variance weighted
MR Egger
Simple mode
Weighted median
Weighted mode
0.25
0.00
−0.25
−0.50
−0.75
0.04
0.08
0.12
0.16

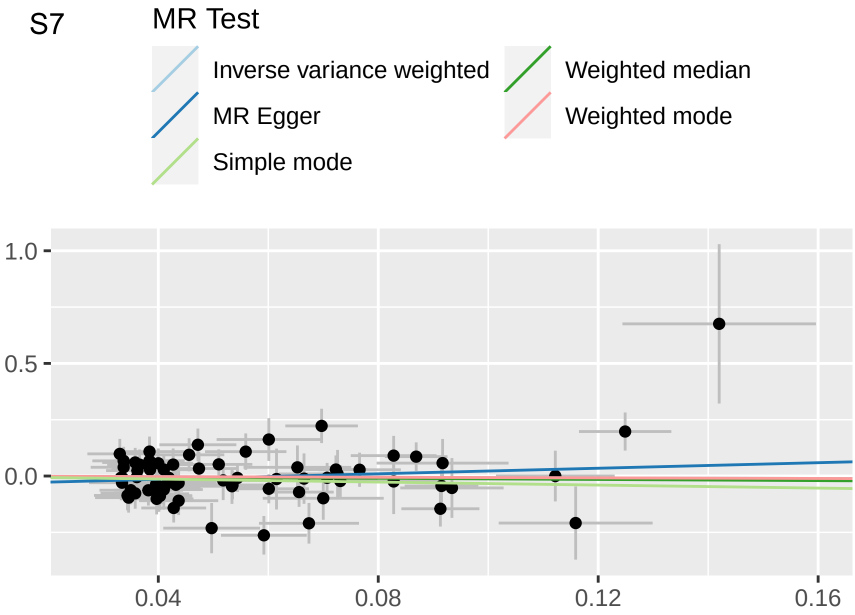
S7
MR Test
Inverse variance weighted
MR Egger
Simple mode
Weighted median
Weighted mode
1.0
0.5
0.0
0.04
0.08
0.12
0.16

S8

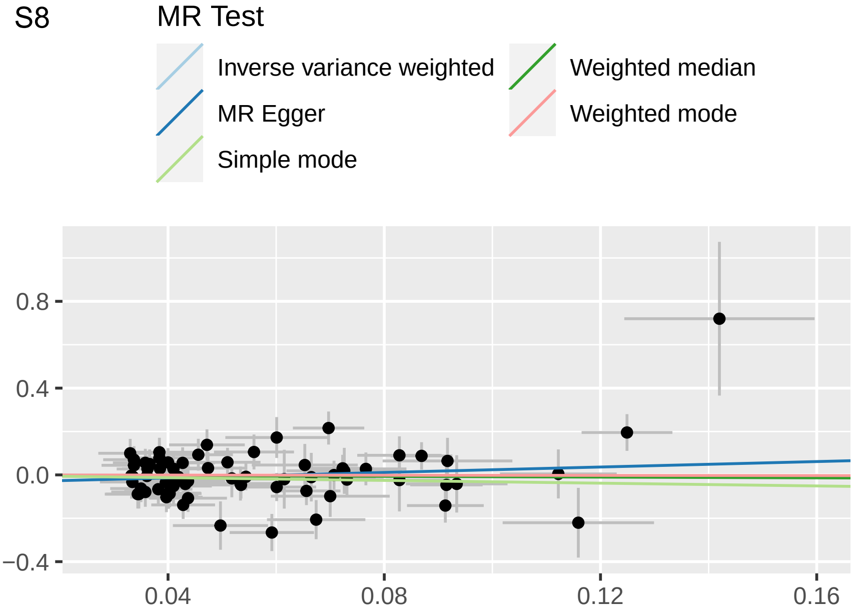
MR Test
Inverse variance weighted
MR Egger
Simple mode
Weighted median
Weighted mode
0.8
0.4
0.0
−0.4
0.04
0.08
0.12
0.16

S9

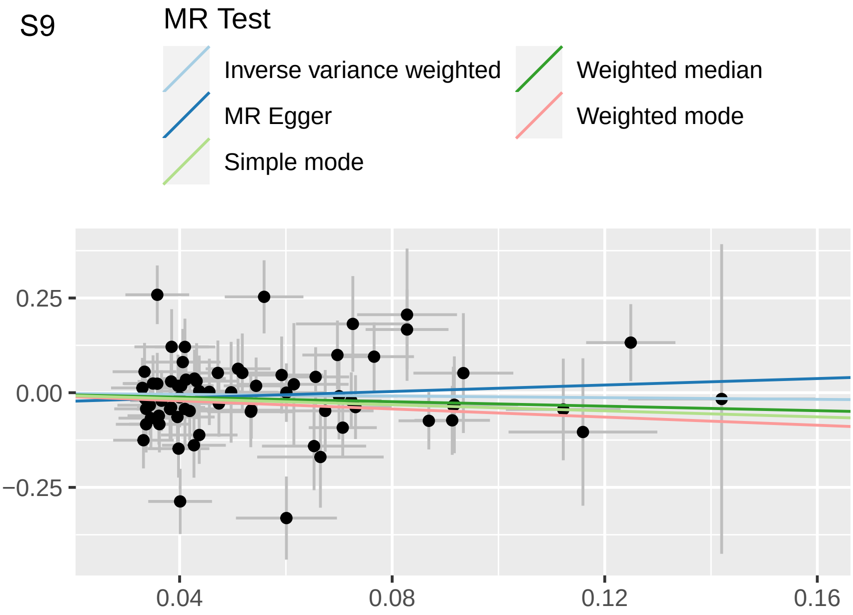
MR Test
Inverse variance weighted
MR Egger
Simple mode
Weighted median
Weighted mode
0.25
0.00
−0.25
0.04
0.08
0.12
0.16

S10

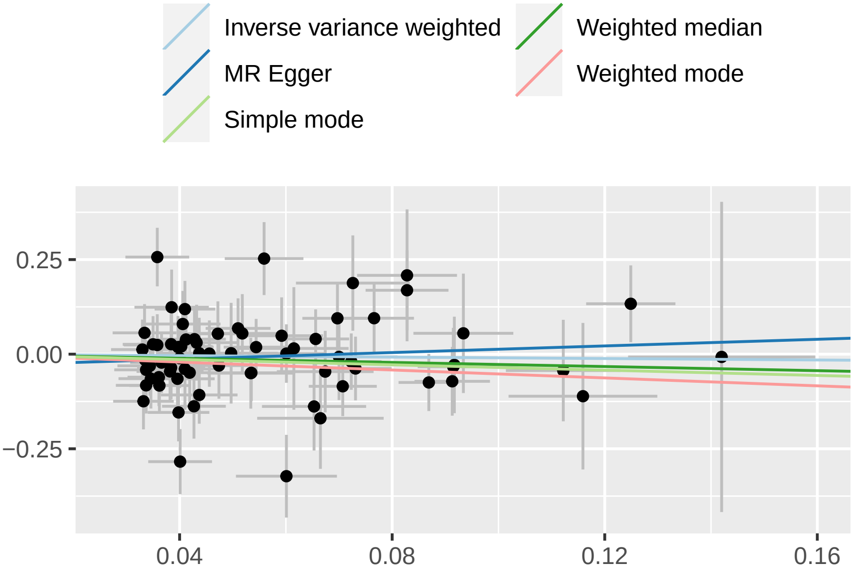
MR Test
Inverse variance weighted
MR Egger
Simple mode
Weighted median
Weighted mode
0.25
0.00
−0.25
0.04
0.08
0.12
0.16

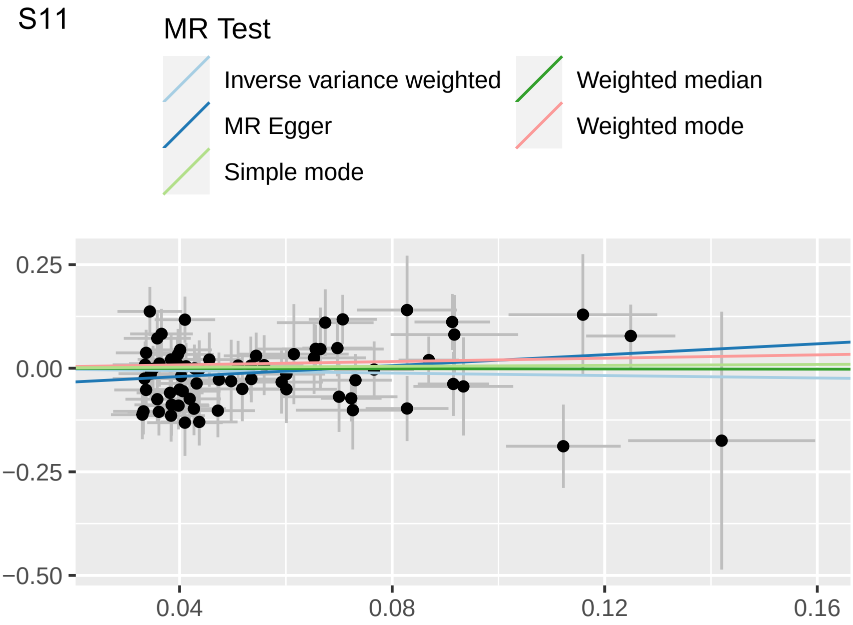
S11
MR Test
Inverse variance weighted
MR Egger
Simple mode
Weighted median
Weighted mode
0.25
0.00
−0.25
−0.50
0.04
0.08
0.12
0.16

S12

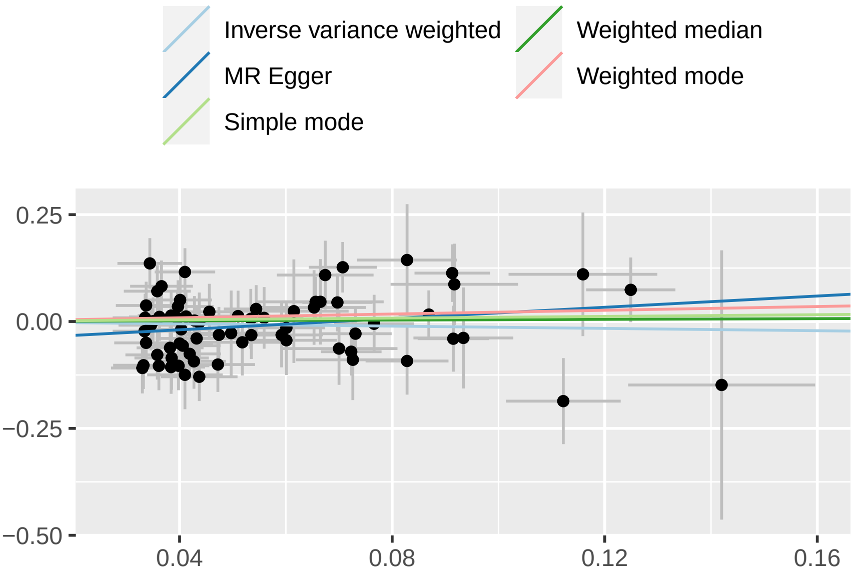
MR Test
Inverse variance weighted
MR Egger
Simple mode
Weighted median
Weighted mode
0.25
0.00
−0.25
−0.50
0.04
0.08
0.12
0.16

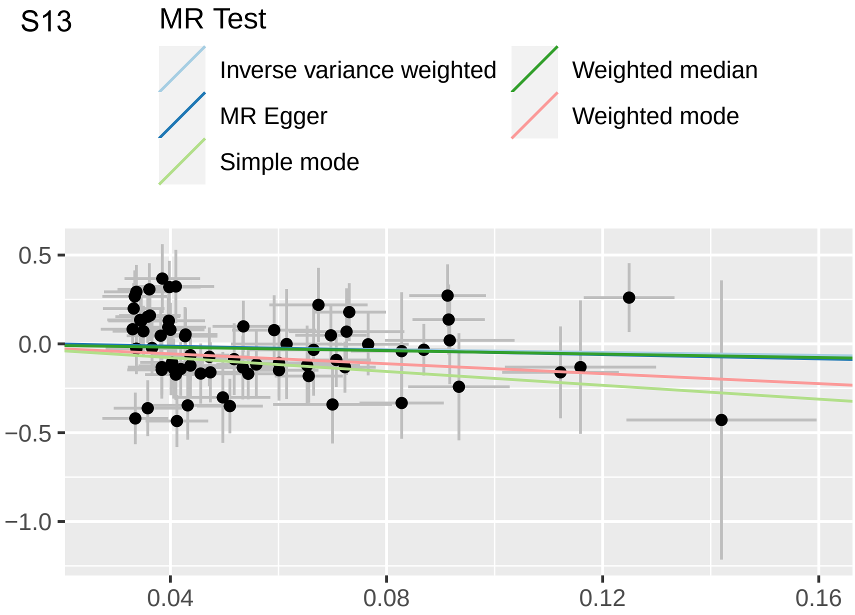
S13
MR Test
Inverse variance weighted
MR Egger
Simple mode
Weighted median
Weighted mode
0.5
0.0
−0.5
−1.0
0.04
0.08
0.12
0.16

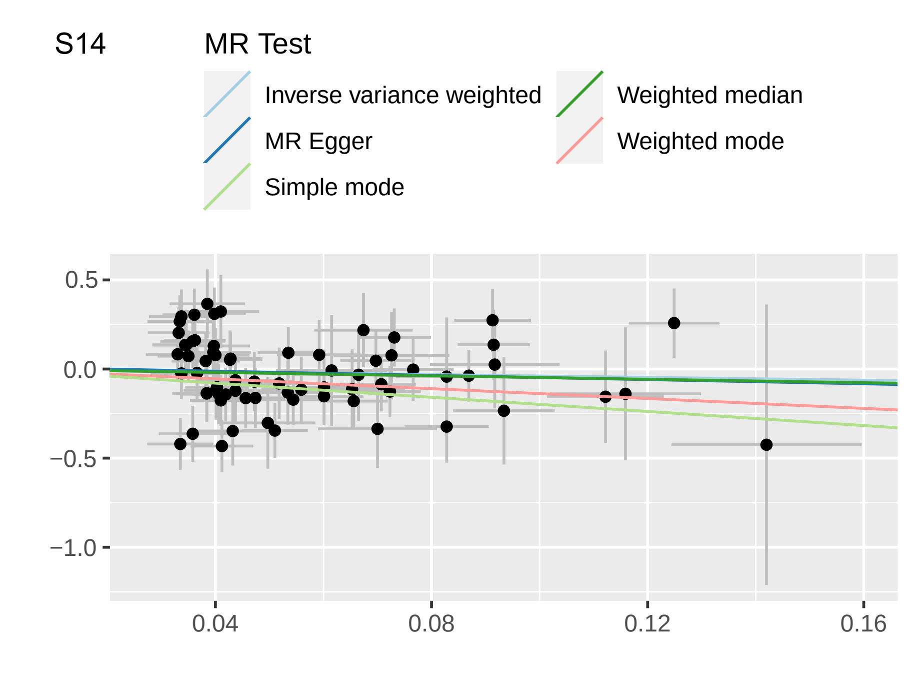
S14
MR Test
Inverse variance weighted
MR Egger
Simple mode
Weighted median
Weighted mode
0.5
0.0
−0.5
−1.0
0.04
0.08
0.12
0.16

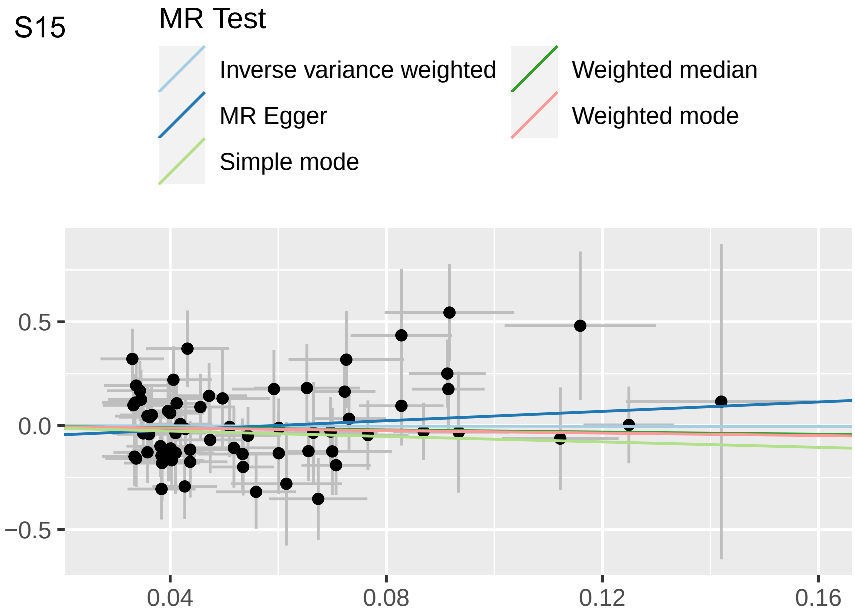
S15
MR Test
Inverse variance weighted
MR Egger
Simple mode
Weighted median
Weighted mode
0.5
0.0
−0.5
0.04
0.08
0.12
0.16

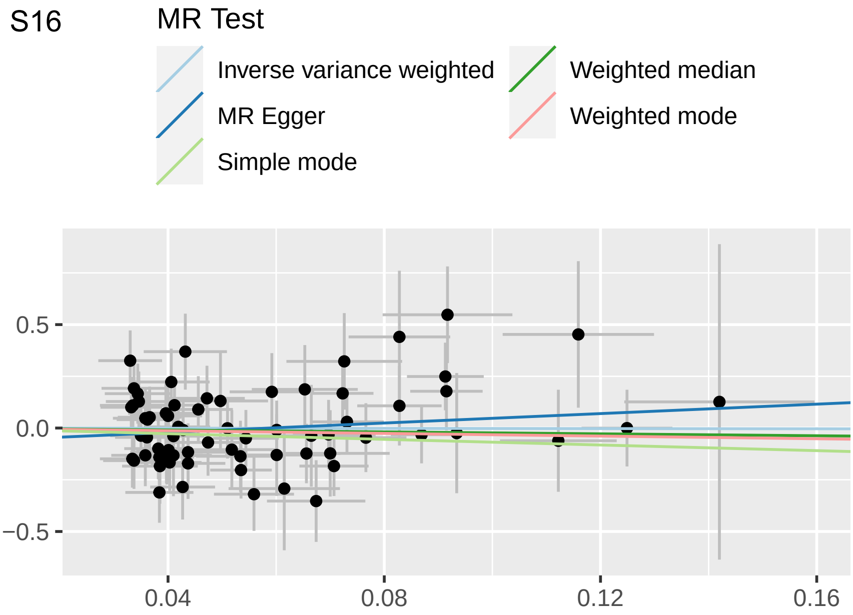
S16
MR Test
Inverse variance weighted
MR Egger
Simple mode
Weighted median
Weighted mode
0.5
0.0
−0.5
0.04
0.08
0.12
0.16

S17

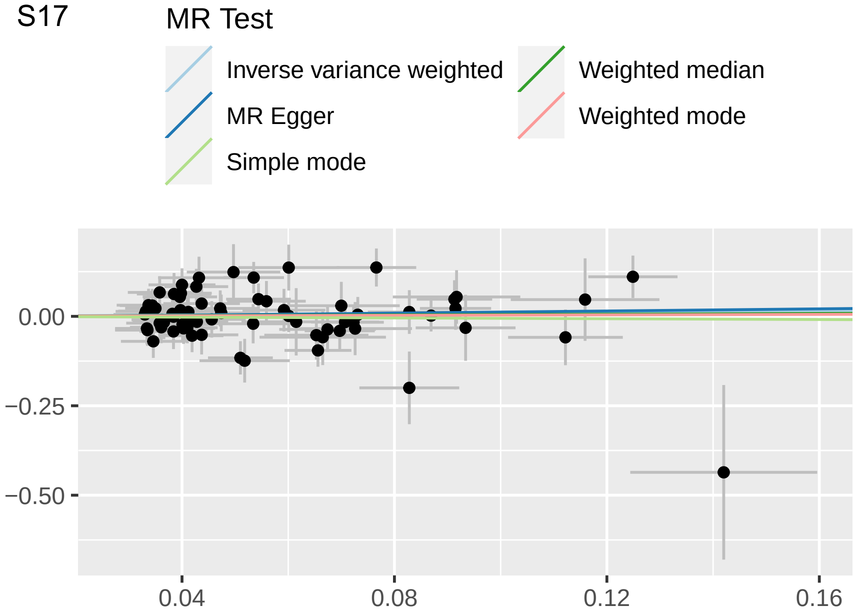
MR Test
Inverse variance weighted
MR Egger
Simple mode
Weighted median
Weighted mode
0.00
−0.25
−0.50
0.04
0.08
0.12
0.16

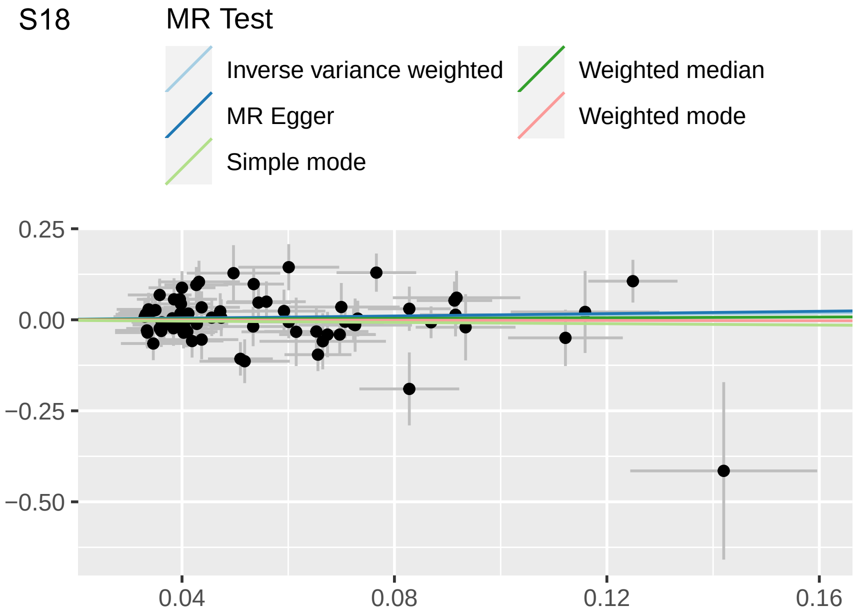
S18
MR Test
Inverse variance weighted
MR Egger
Simple mode
Weighted median
Weighted mode
0.25
0.00
−0.25
−0.50
0.04
0.08
0.12
0.16

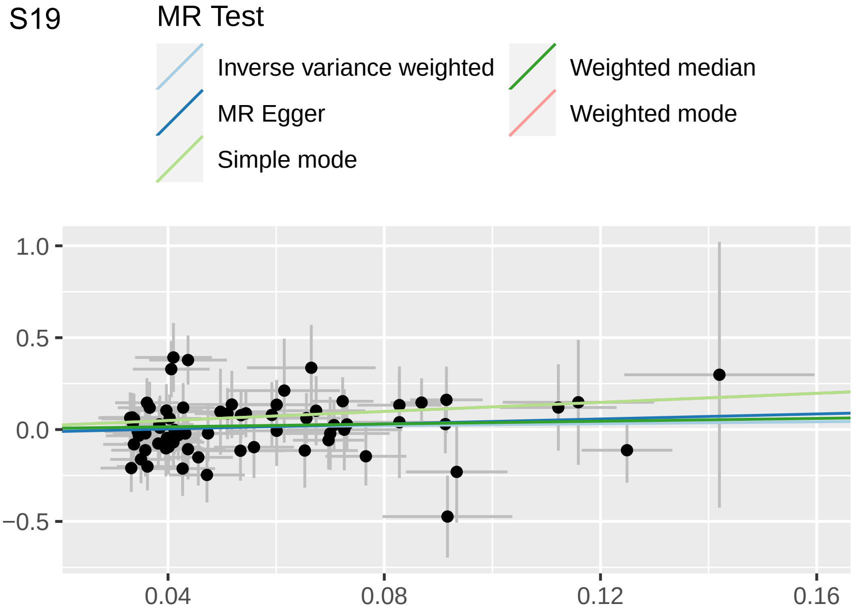
S19
MR Test
Inverse variance weighted
MR Egger
Simple mode
Weighted median
Weighted mode
1.0
0.5
0.0
−0.5
0.04
0.08
0.12
0.16

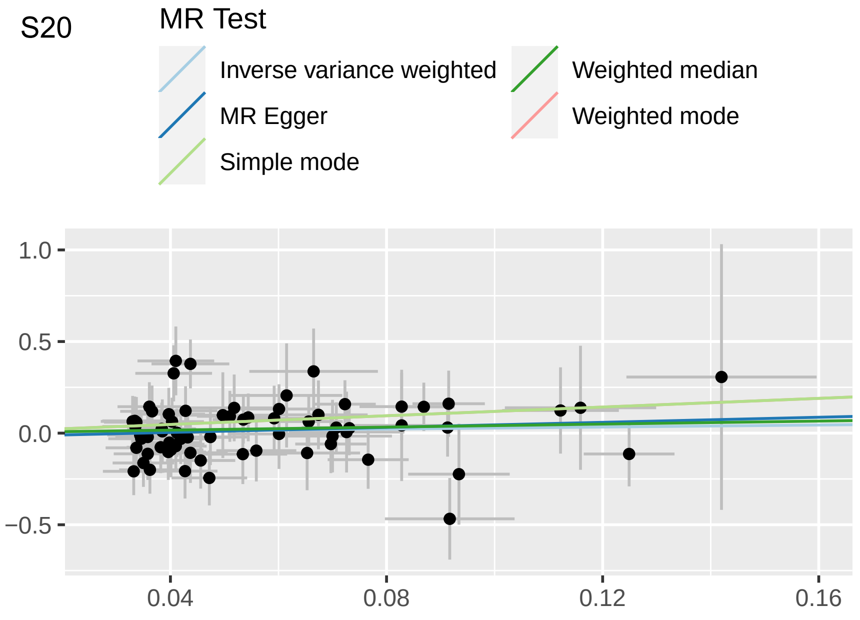
S20
MR Test
Inverse variance weighted
MR Egger
Simple mode
Weighted median
Weighted mode
1.0
0.5
0.0
−0.5
0.04
0.08
0.12
0.16

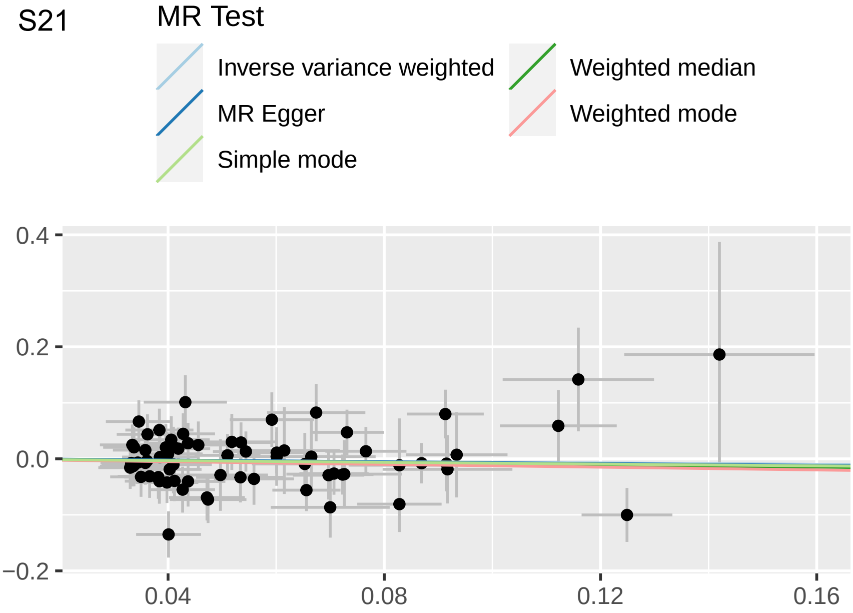

S21
MR Test
Inverse variance weighted
MR Egger
Simple mode
Weighted median
Weighted mode
0.4
0.2
0.0
−0.2
0.04
0.08
0.12
0.16

S22

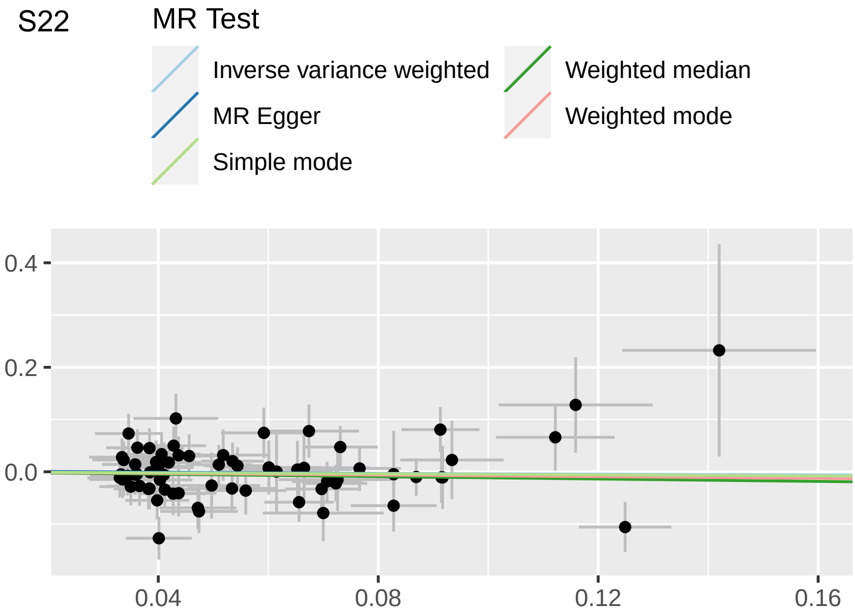
MR Test
Inverse variance weighted
MR Egger
Simple mode
Weighted median
Weighted mode
0.4
0.2
0.0
0.04
0.08
0.12
0.16

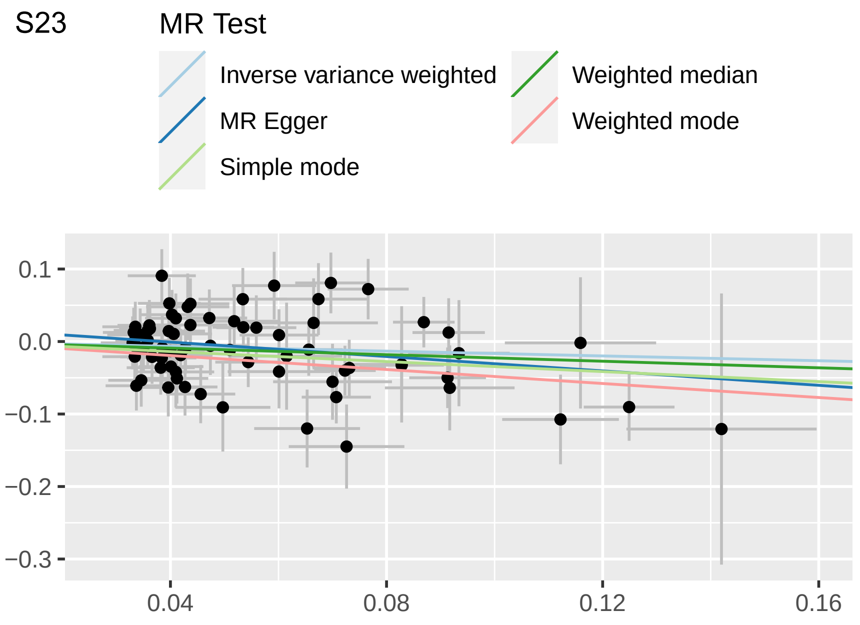

S23
MR Test
Inverse variance weighted
MR Egger
Simple mode
Weighted median
Weighted mode
0.1
0.0
−0.1
−0.2
−0.3
0.04
0.08
0.12
0.16

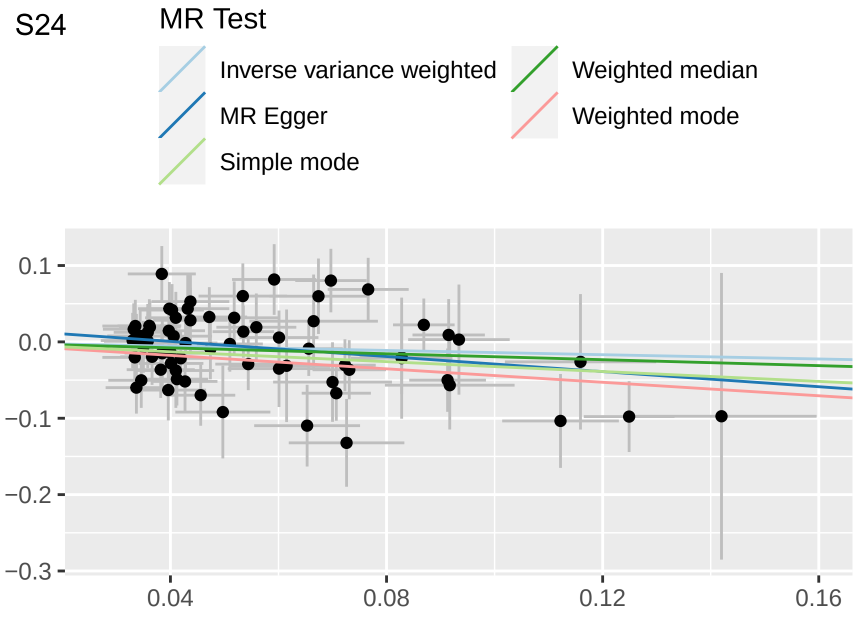
S24
MR Test
Inverse variance weighted
MR Egger
Simple mode
Weighted median
Weighted mode
0.1
0.0
−0.1
−0.2
−0.3
0.04
0.08
0.12
0.16

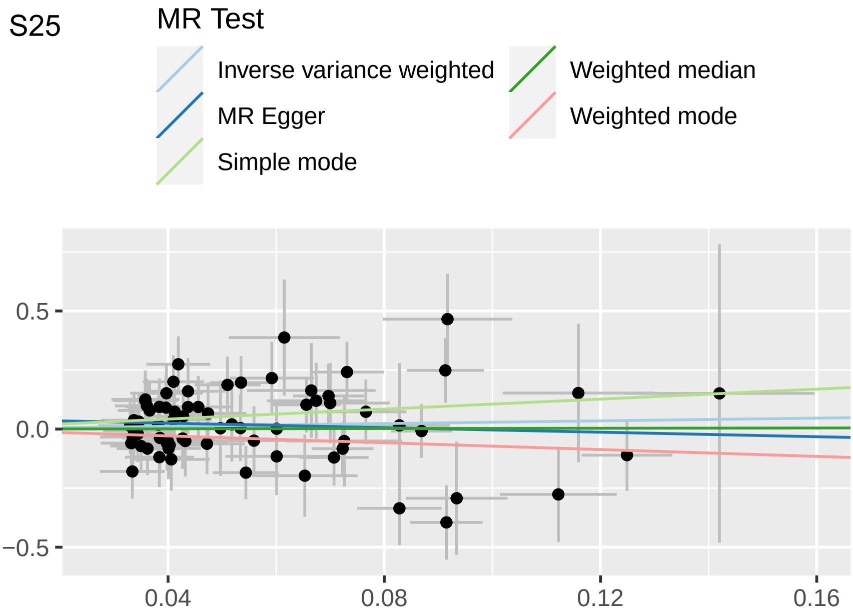
S25
MR Test
Inverse variance weighted
MR Egger
Simple mode
Weighted median
Weighted mode
0.5
0.0
−0.5
0.04
0.08
0.12
0.16

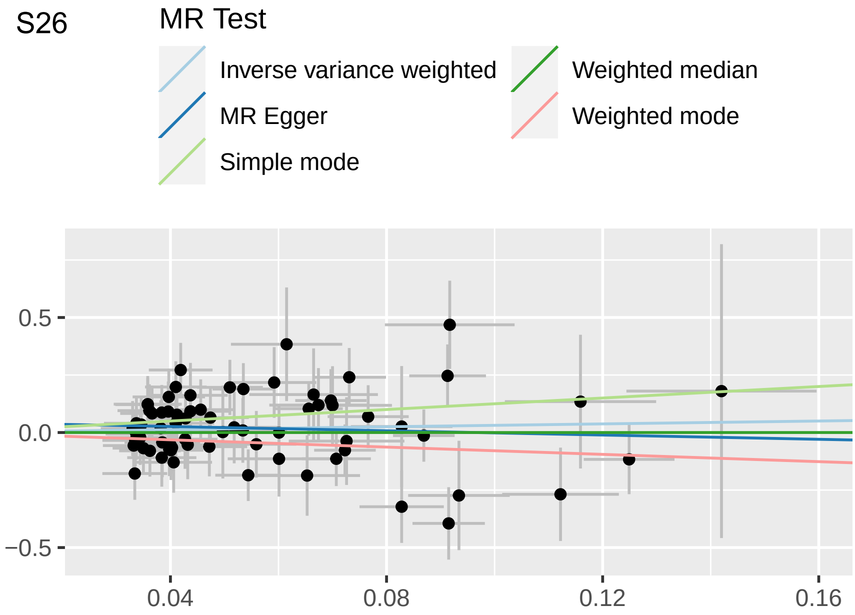
S26
MR Test
Inverse variance weighted
MR Egger
Simple mode
Weighted median
Weighted mode
0.5
0.0
−0.5
0.04
0.08
0.12
0.16

S27

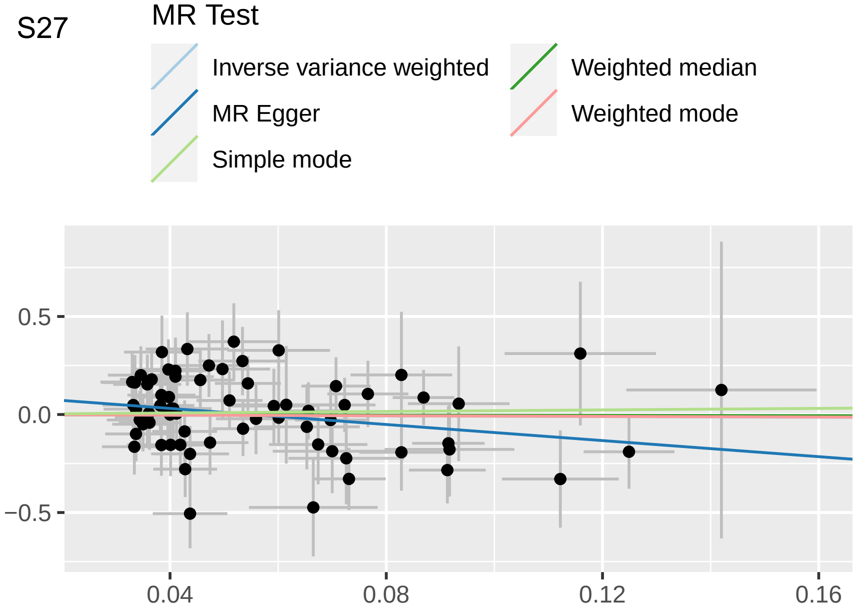
MR Test
Inverse variance weighted
MR Egger
Simple mode
Weighted median
Weighted mode
0.5
0.0
−0.5
0.04
0.08
0.12
0.16

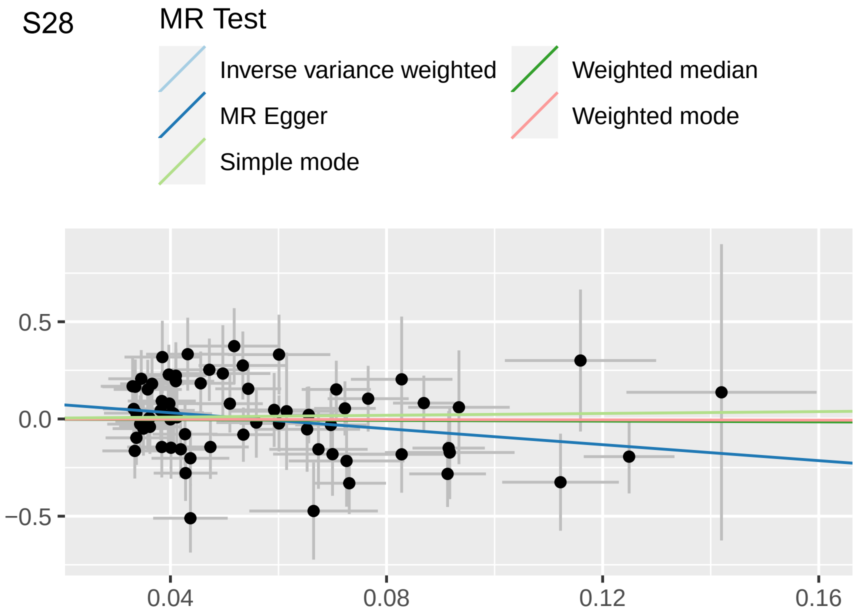

S28
MR Test
Inverse variance weighted
Weighted median
MR Egger
Weighted mode
Simple mode
0.5
0.0
−0.5
0.04
0.08
0.12
0.16

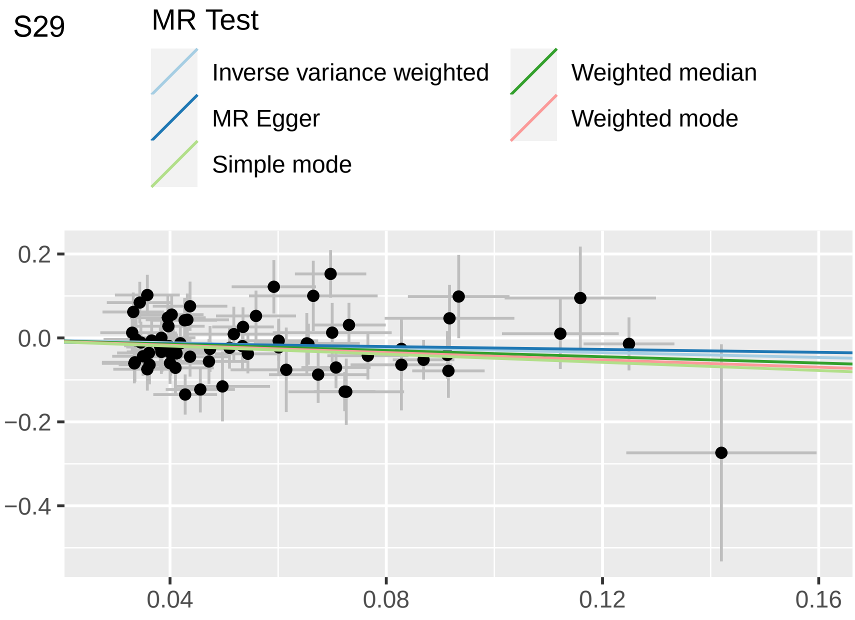
S29
MR Test
Inverse variance weighted
MR Egger
Simple mode
Weighted median
Weighted mode
0.2
0.0
−0.2
−0.4
0.04
0.08
0.12
0.16

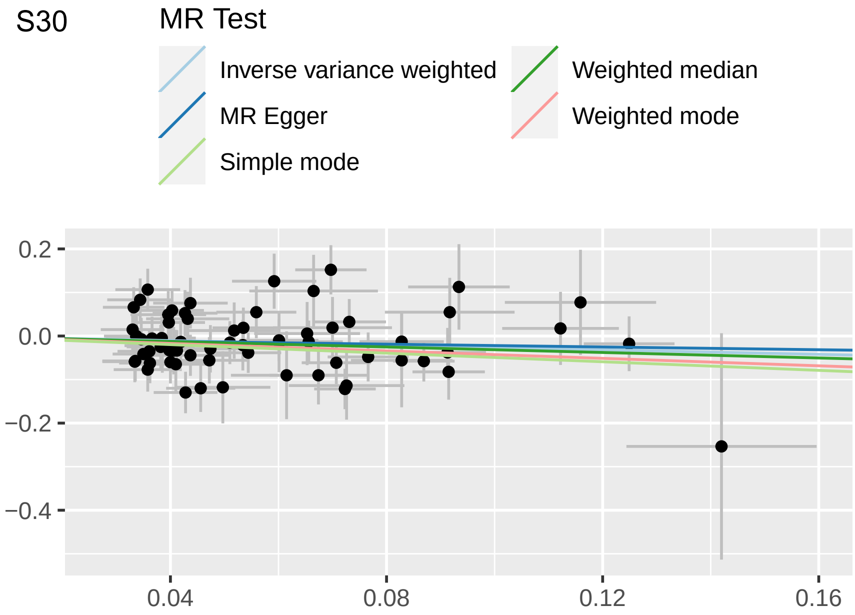
S30
MR Test
Inverse variance weighted
MR Egger
Simple mode
Weighted median
Weighted mode
0.2
0.0
−0.2
−0.4
0.04
0.08
0.12
0.16

S31

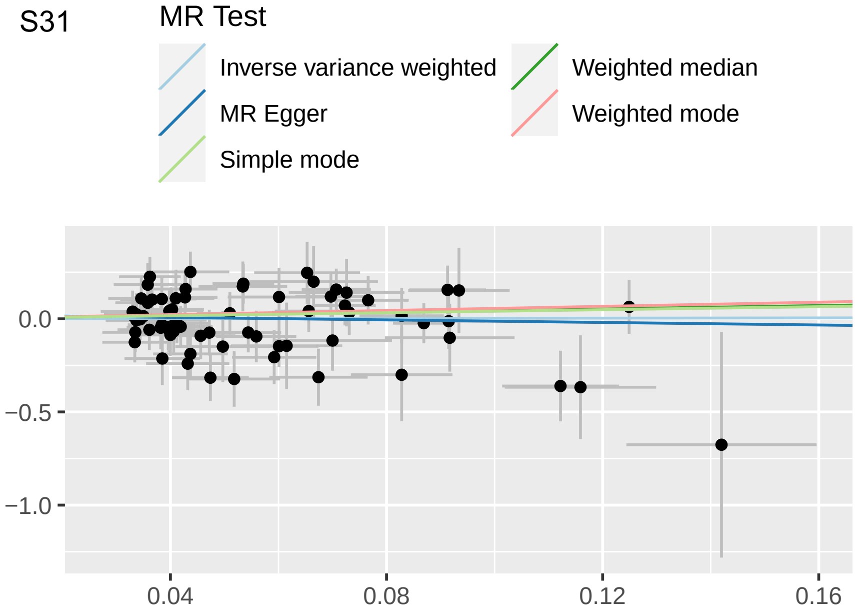
MR Test
Inverse variance weighted
MR Egger
Simple mode
Weighted median
Weighted mode
0.0
−0.5
−1.0
0.04
0.08
0.12
0.16

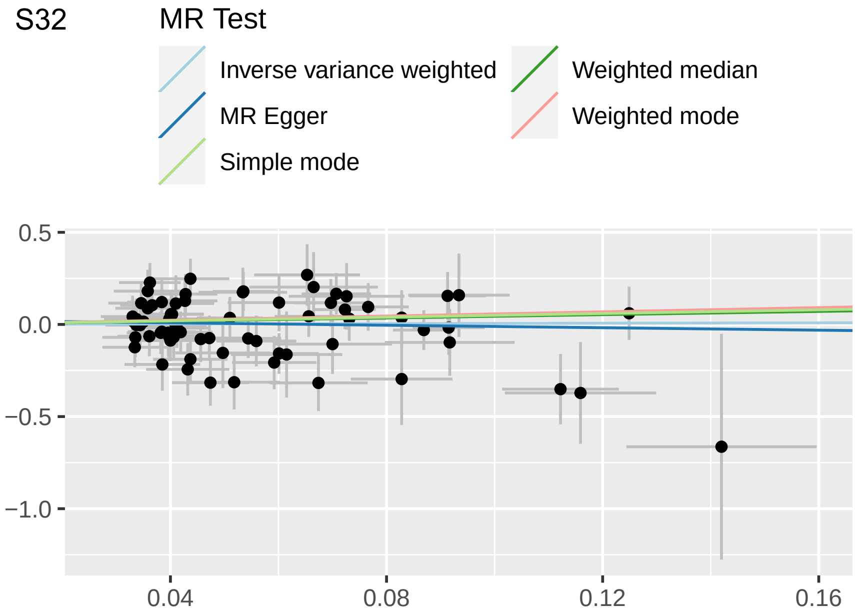

S32
MR Test
Inverse variance weighted
MR Egger
Simple mode
Weighted median
Weighted mode
0.5
0.0
−0.5
−1.0
0.04
0.08
0.12
0.16

S33

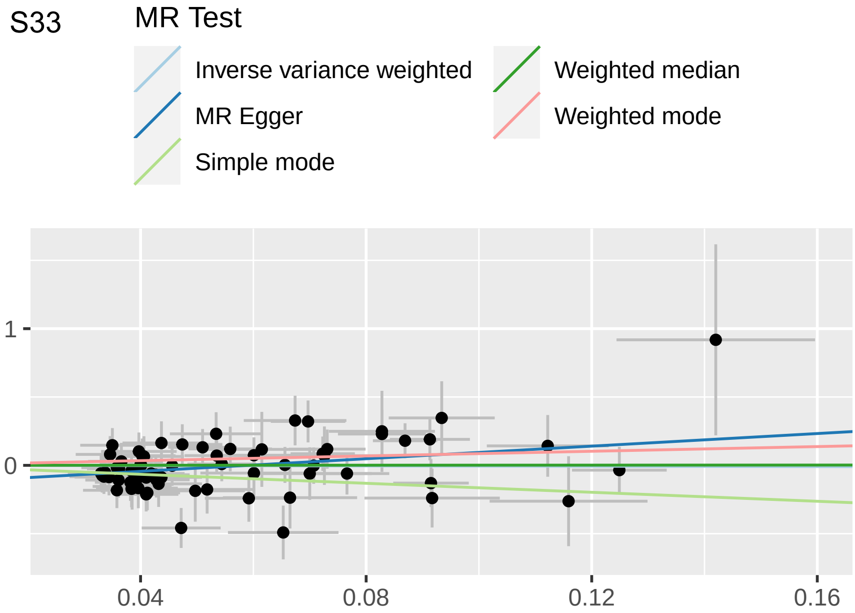
MR Test
Inverse variance weighted
MR Egger
Simple mode
Weighted median
Weighted mode
1
0
0.04
0.08
0.12
0.16

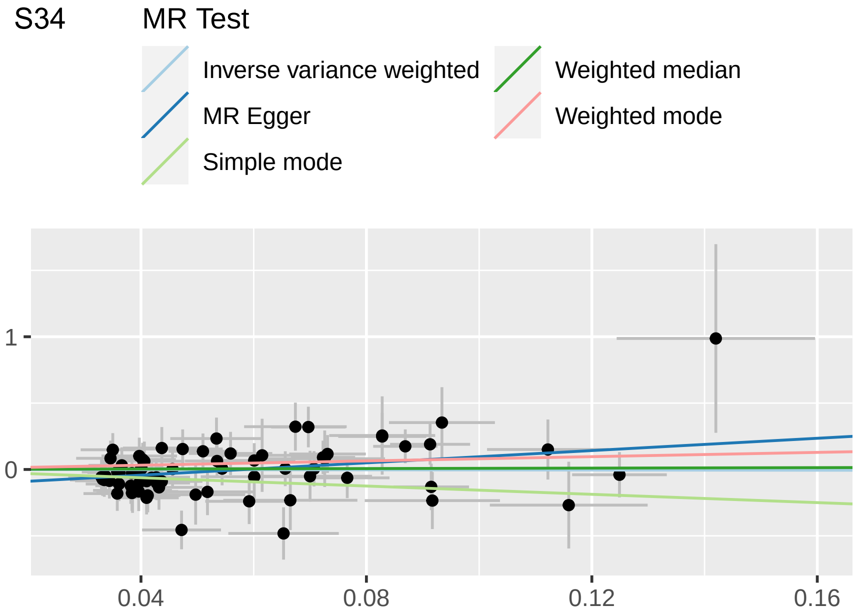

S34
MR Test
Inverse variance weighted
MR Egger
Simple mode
Weighted median
Weighted mode
1
0
0.04
0.08
0.12
0.16

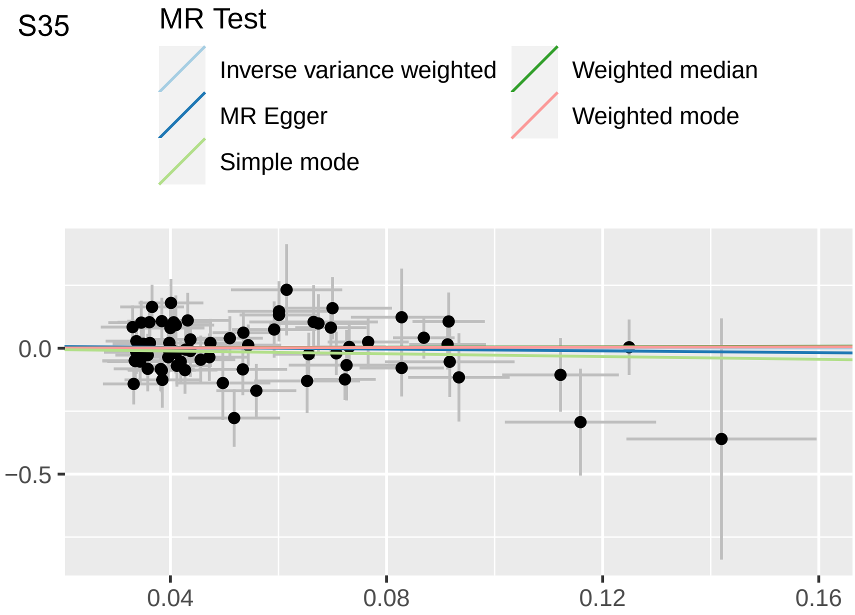
S35
MR Test
Inverse variance weighted
MR Egger
Simple mode
Weighted median
Weighted mode
0.0
−0.5
0.04
0.08
0.12
0.16

S36

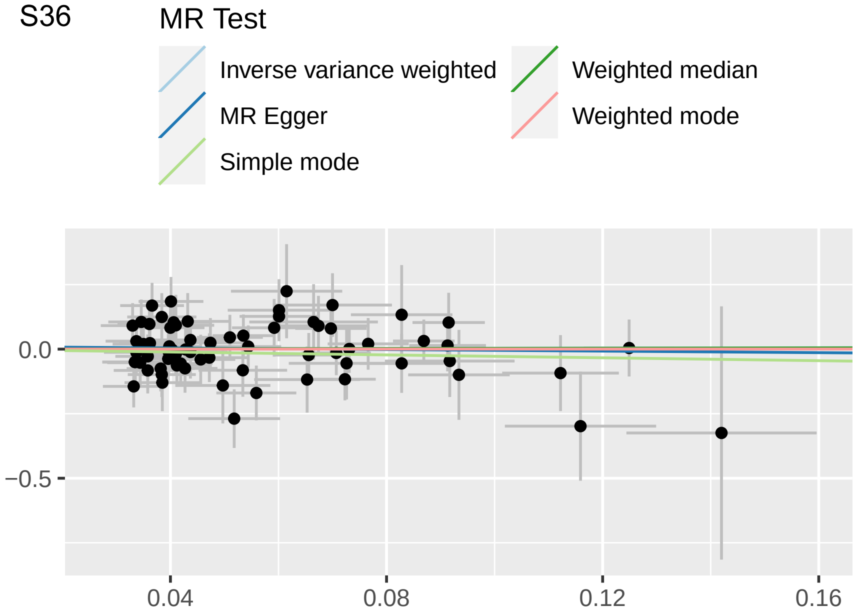

MR Test
Inverse variance weighted
MR Egger
Simple mode
Weighted median
Weighted mode
0.0
−0.5
0.04
0.08
0.12
0.16

S37

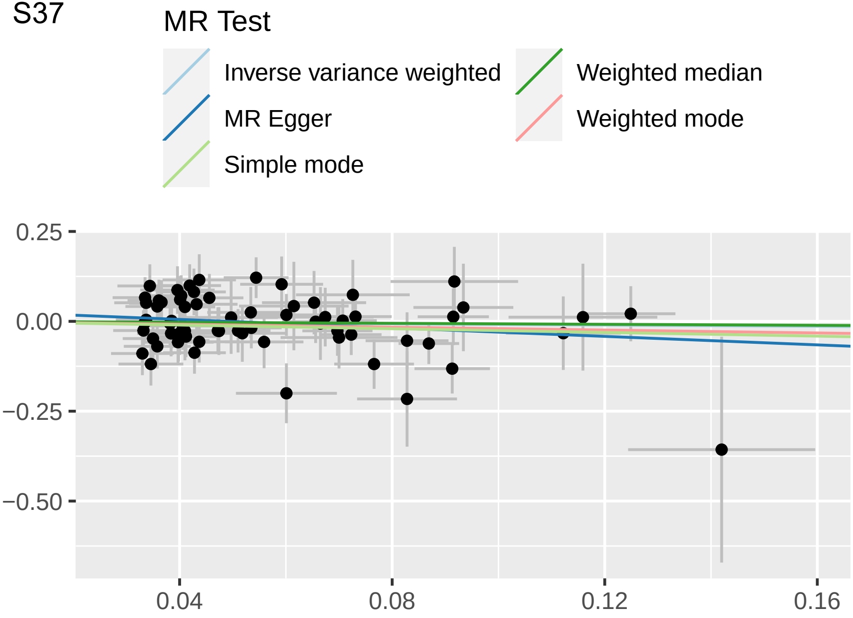
MR Test
Inverse variance weighted
MR Egger
Simple mode
Weighted median
Weighted mode
0.25
0.00
−0.25
−0.50
0.04
0.08
0.12
0.16

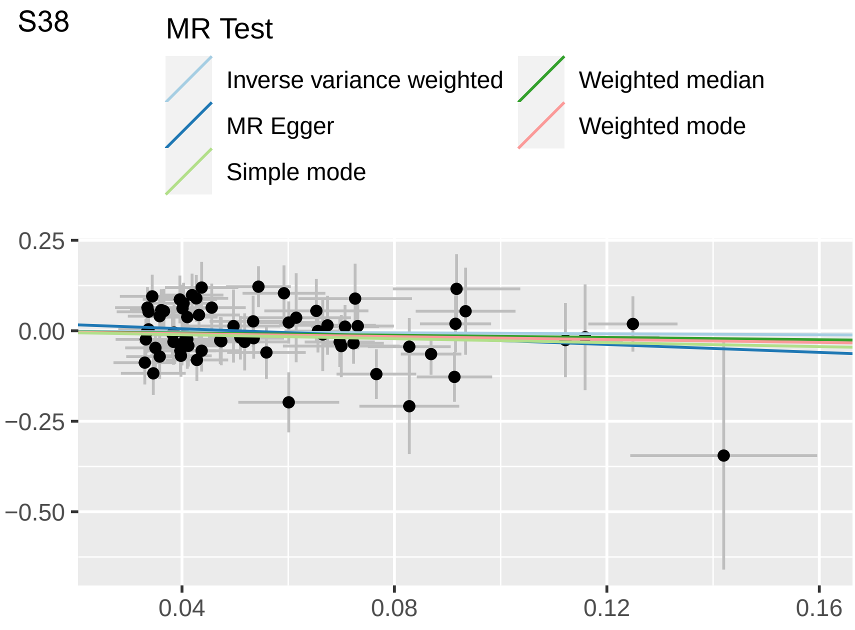
S38
MR Test
Inverse variance weighted
MR Egger
Simple mode
Weighted median
Weighted mode
0.25
0.00
−0.25
−0.50
0.04
0.08
0.12
0.16

S39

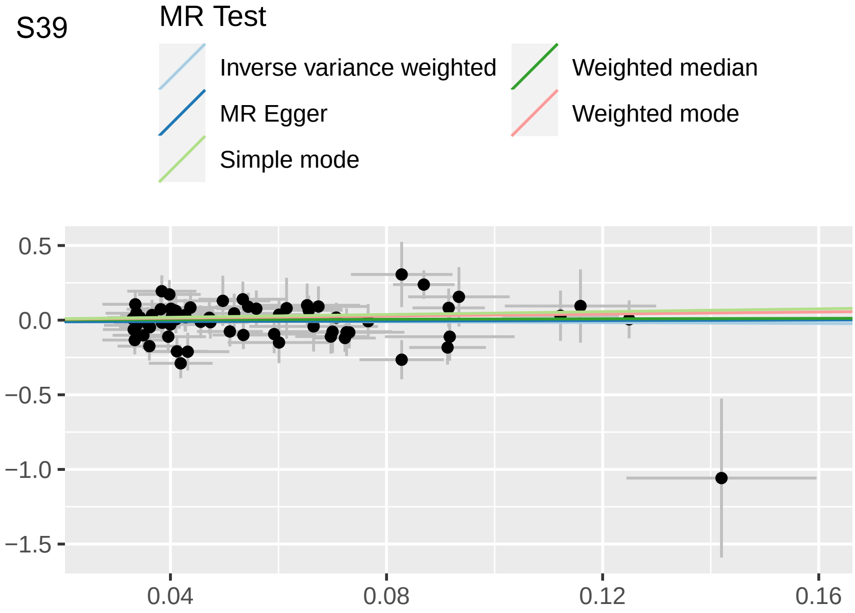

MR Test
Inverse variance weighted
MR Egger
Simple mode
Weighted median
Weighted mode
0.5
0.0
−0.5
−1.0
−1.5
0.04
0.08
0.12
0.16

S40

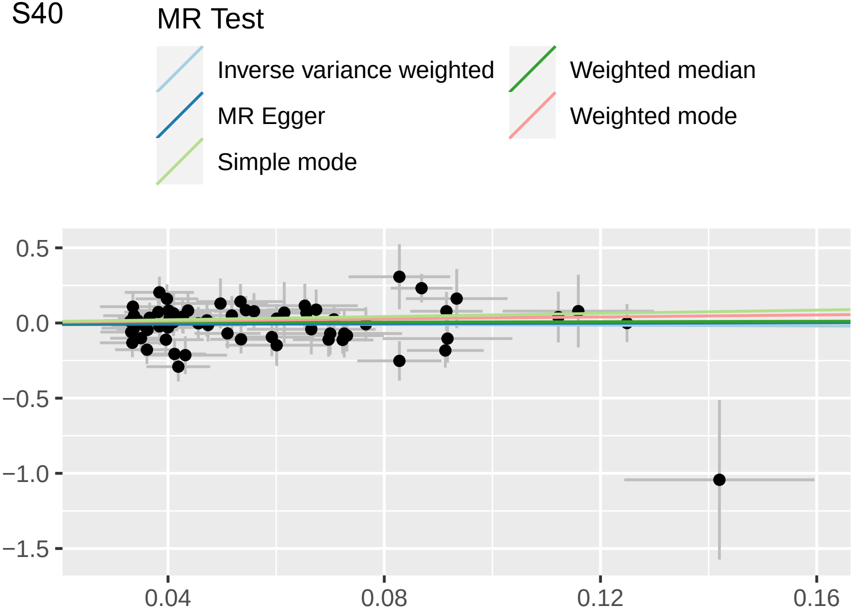
MR Test
Inverse variance weighted
MR Egger
Simple mode
Weighted median
Weighted mode
0.5
0.0
−0.5
−1.0
−1.5
0.04
0.08
0.12
0.16

S41

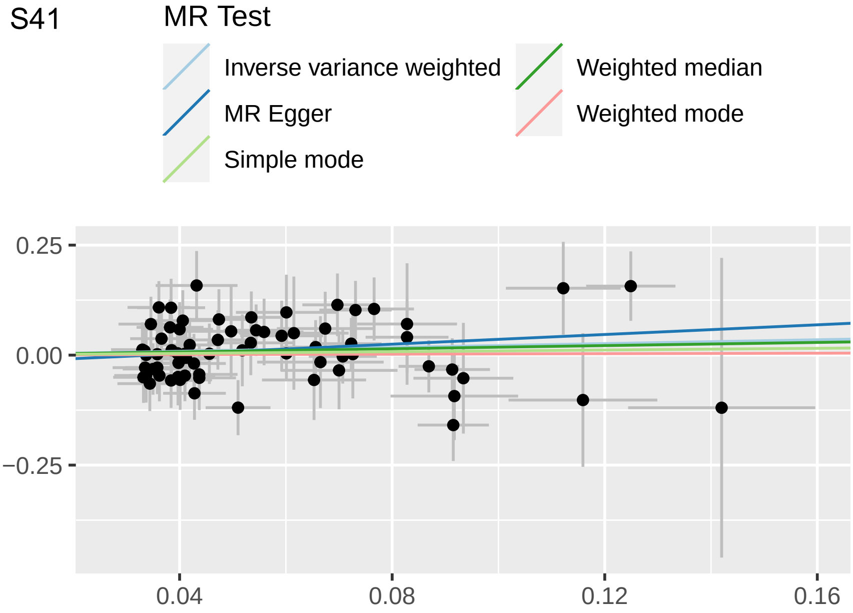
MR Test
Inverse variance weighted
MR Egger
Simple mode
Weighted median
Weighted mode
0.25
0.00
−0.25
0.04
0.08
0.12
0.16

S42

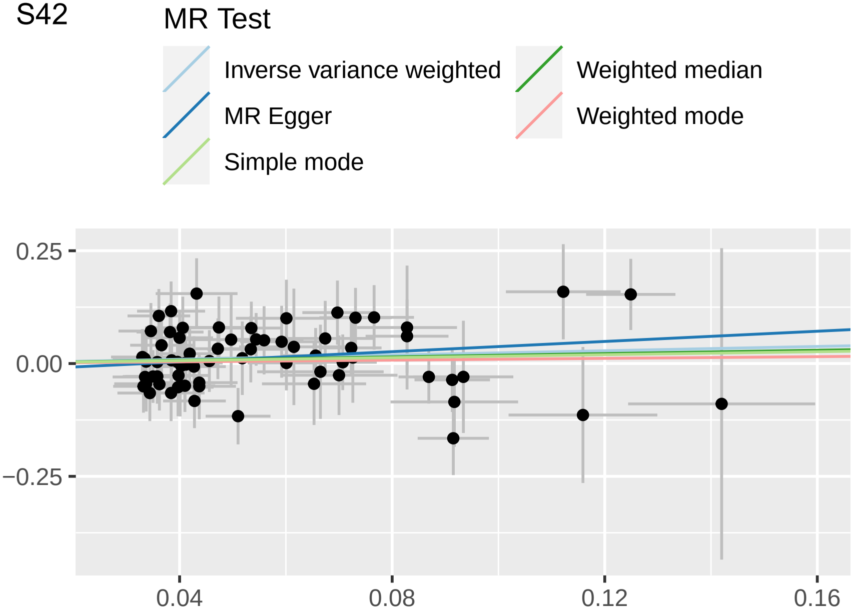
MR Test
Inverse variance weighted
MR Egger
Simple mode
Weighted median
Weighted mode
0.25
0.00
−0.25
0.04
0.08
0.12
0.16

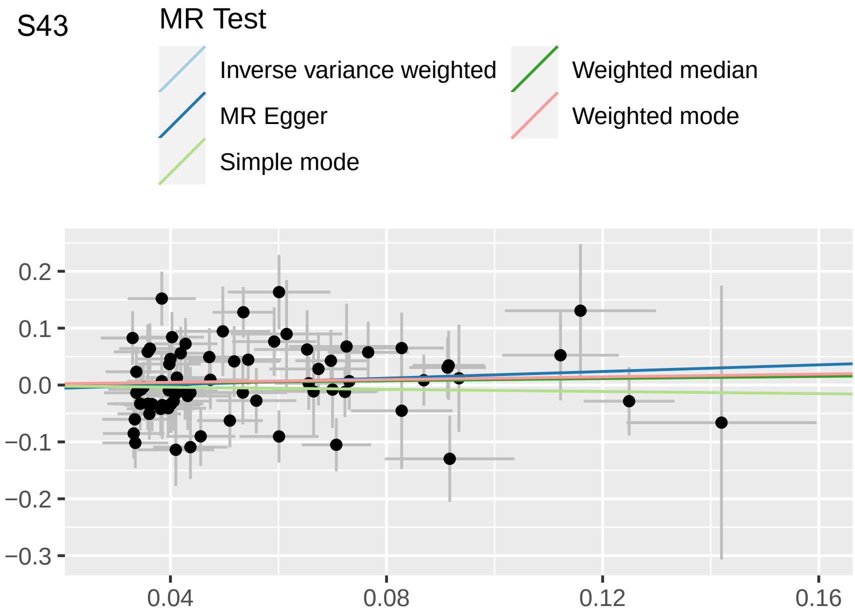

S43
MR Test
Inverse variance weighted
MR Egger
Simple mode
Weighted median
Weighted mode
0.2
0.1
0.0
−0.1
−0.2
−0.3
0.04
0.08
0.12
0.16

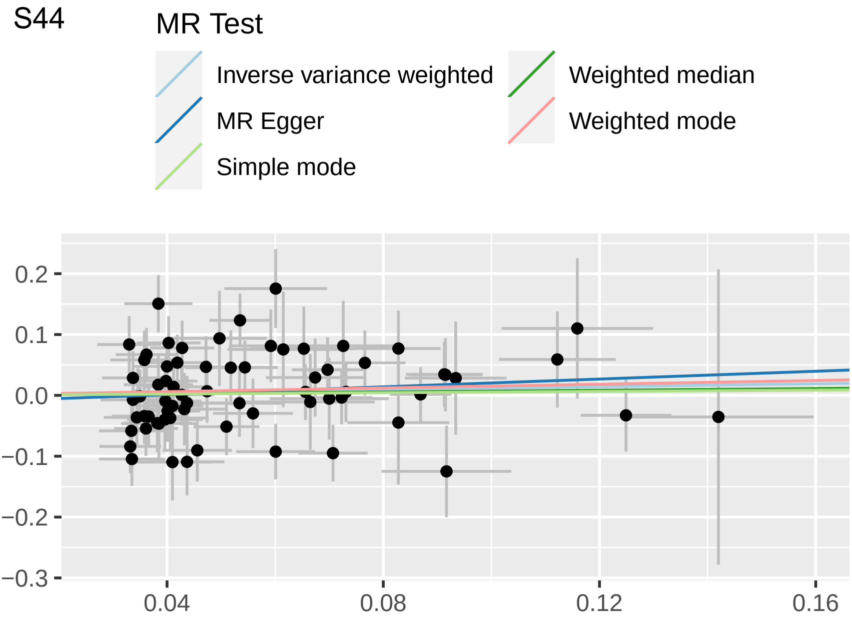
S44
MR Test
Inverse variance weighted
MR Egger
Simple mode
Weighted median
Weighted mode
0.2
0.1
0.0
−0.1
−0.2
−0.3
0.04
0.08
0.12
0.16

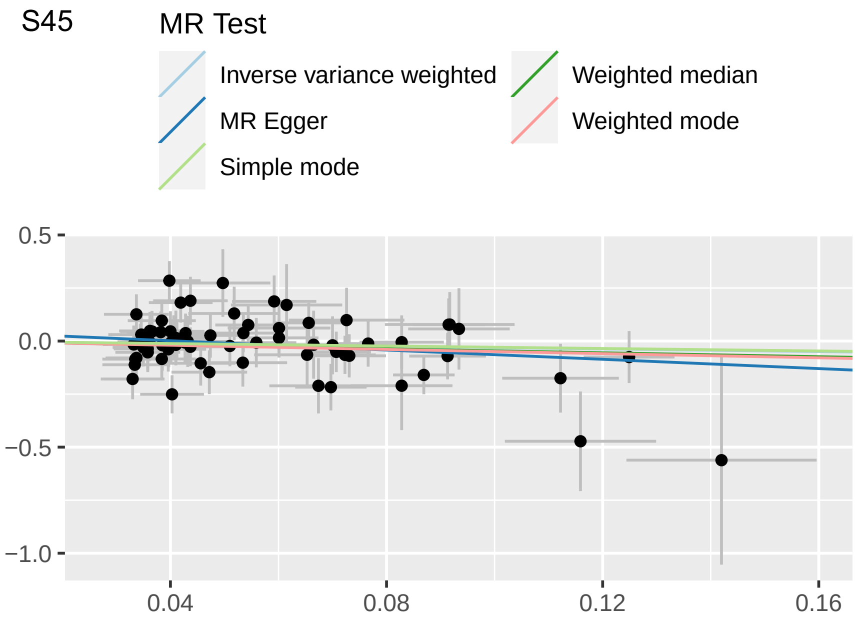
S45
MR Test
Inverse variance weighted
MR Egger
Simple mode
Weighted median
Weighted mode
0.5
0.0
−0.5
−1.0
0.04
0.08
0.12
0.16

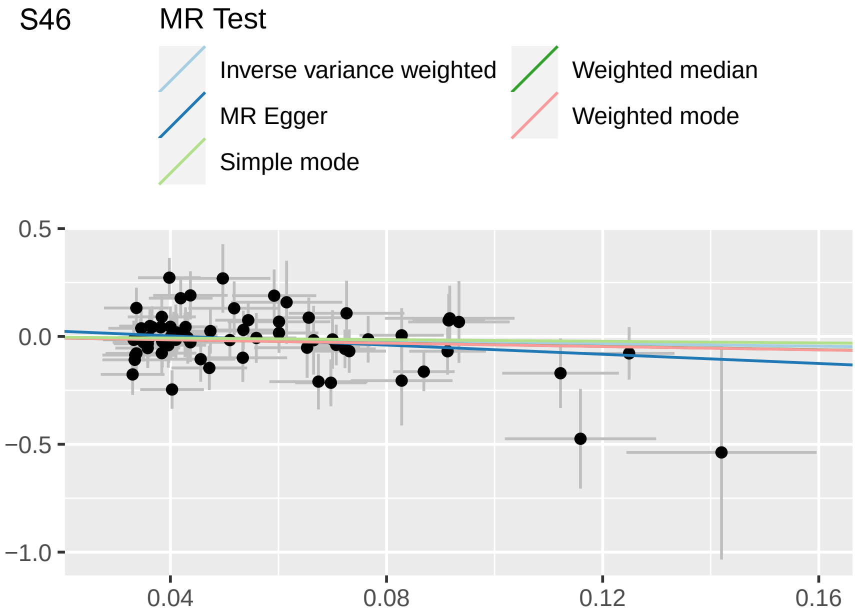
S46
MR Test
Inverse variance weighted
MR Egger
Simple mode
Weighted median
Weighted mode
0.5
0.0
−0.5
−1.0
0.04
0.08
0.12
0.16

S47

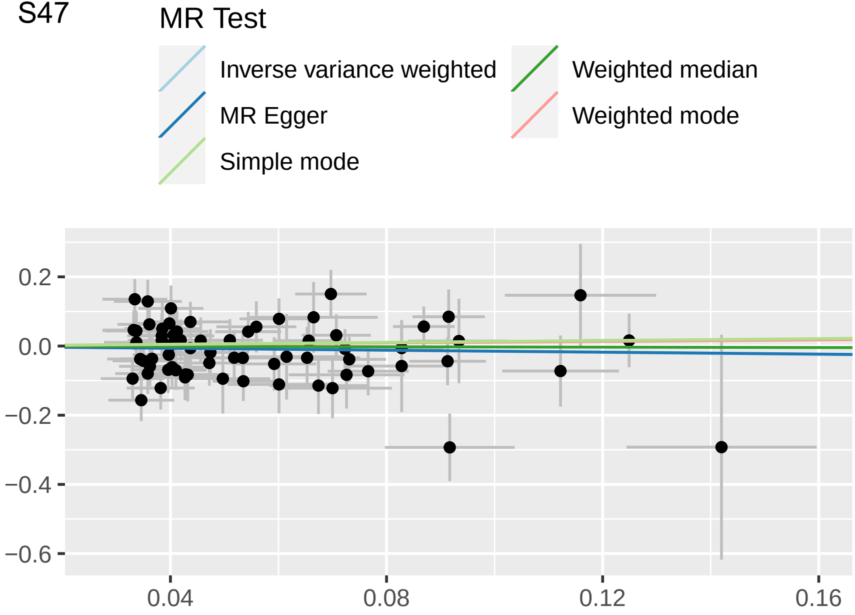

MR Test
Inverse variance weighted
Weighted median
MR Egger
Weighted mode
Simple mode
0.2
0.0
−0.2
−0.4
−0.6
0.04
0.08
0.12
0.16

S48

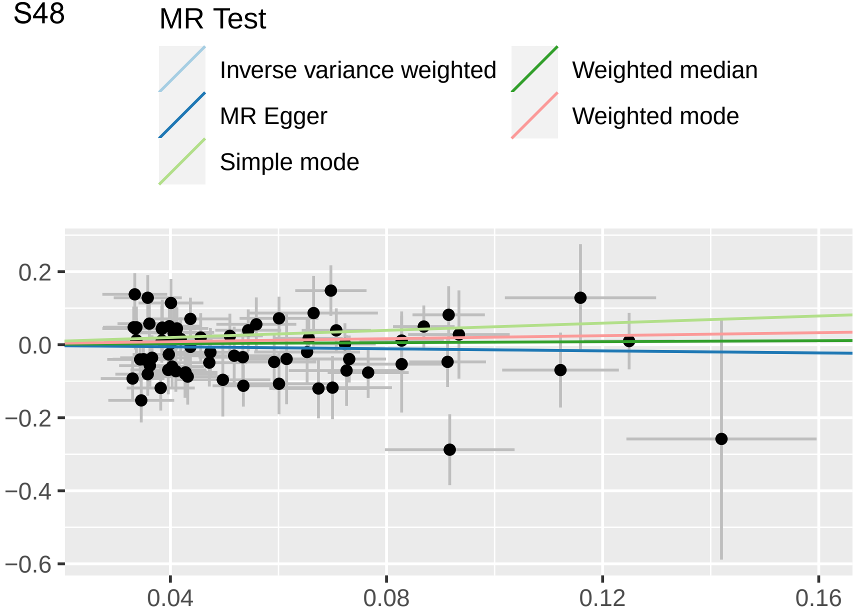
MR Test
Inverse variance weighted
MR Egger
Simple mode
Weighted median
Weighted mode
0.2
0.0
−0.2
−0.4
−0.6
0.04
0.08
0.12
0.16

S49

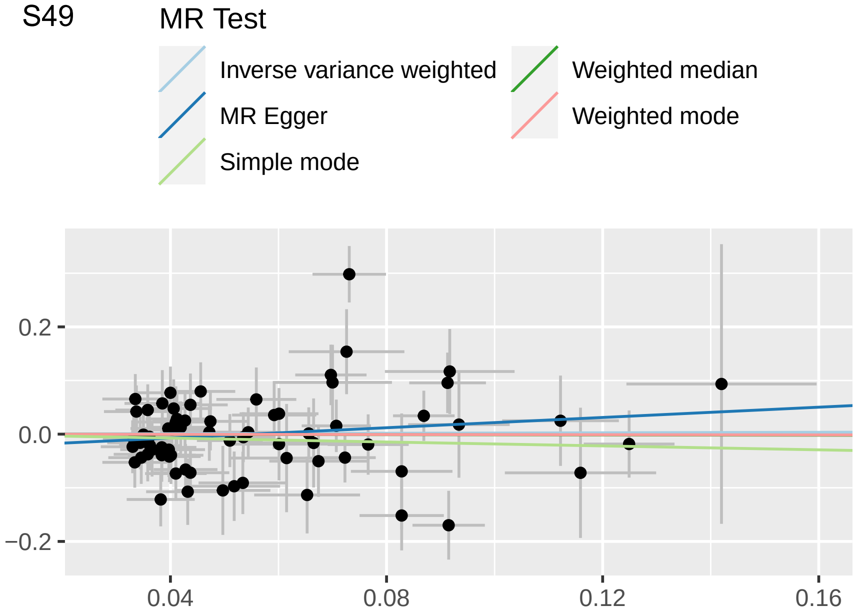
MR Test
Inverse variance weighted
MR Egger
Simple mode
Weighted median
Weighted mode
0.2
0.0
−0.2
0.04
0.08
0.12
0.16

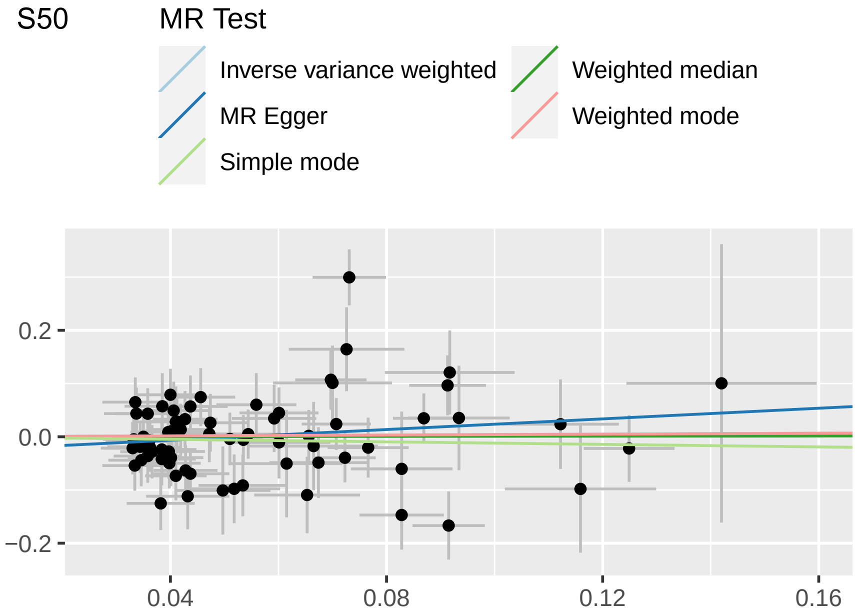

S50
MR Test
Inverse variance weighted
MR Egger
Simple mode
Weighted median
Weighted mode
0.2
0.0
−0.2
0.04
0.08
0.12
0.16

S51

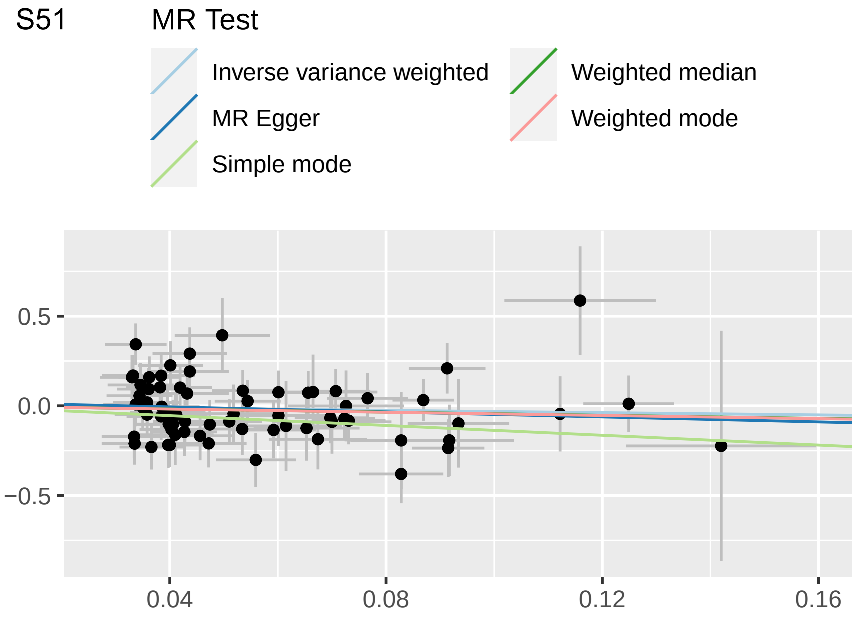
MR Test
Inverse variance weighted
MR Egger
Simple mode
Weighted median
Weighted mode
0.5
0.0
−0.5
0.04
0.08
0.12
0.16

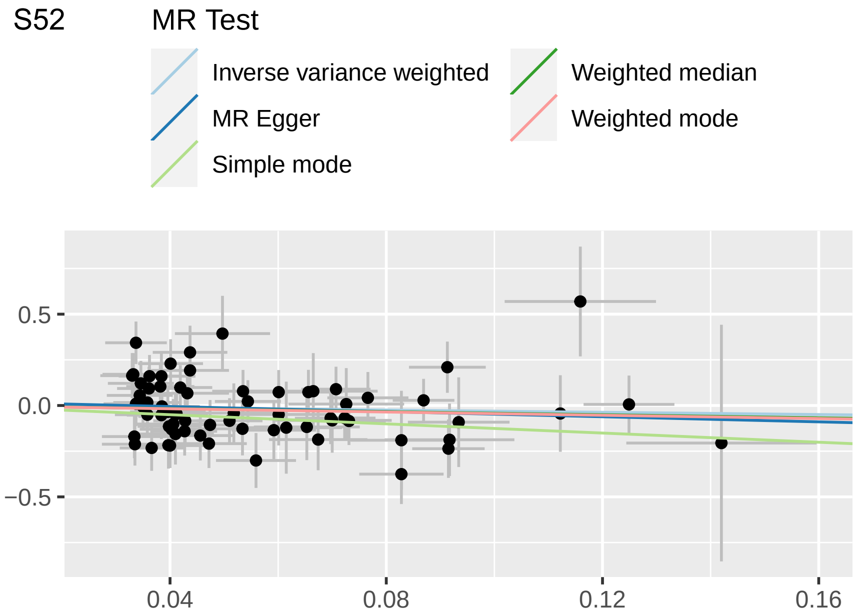
S52
MR Test
Inverse variance weighted
MR Egger
Simple mode
Weighted median
Weighted mode
0.5
0.0
−0.5
0.04
0.08
0.12
0.16

S53

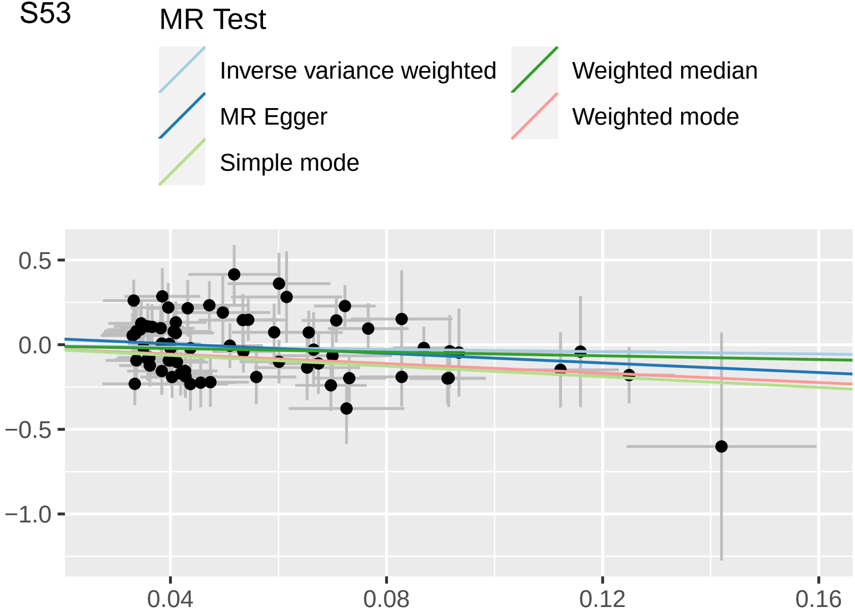
MR Test
Inverse variance weighted
MR Egger
Simple mode
Weighted median
Weighted mode
0.5
0.0
−0.5
−1.0
0.04
0.08
0.12
0.16

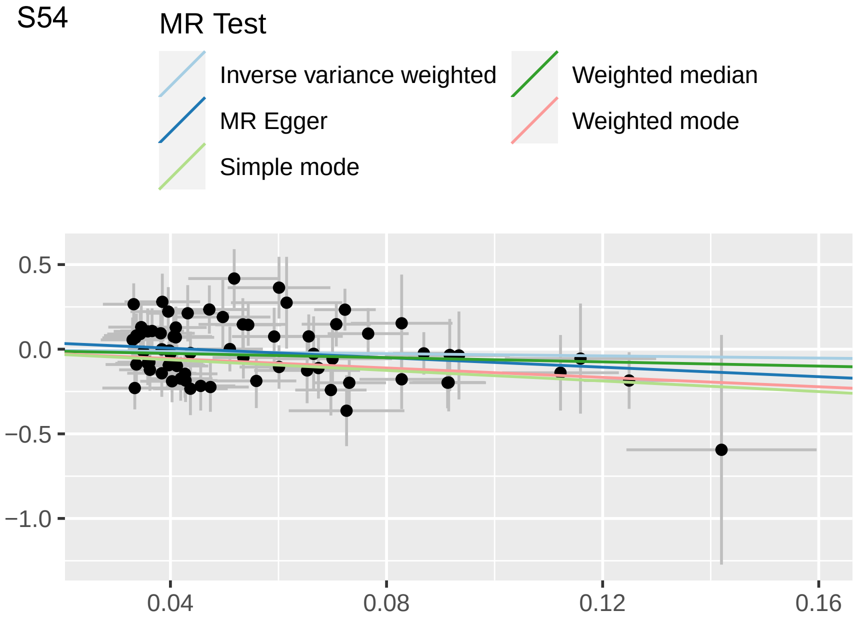
S54
MR Test
Inverse variance weighted
MR Egger
Simple mode
Weighted median
Weighted mode
0.5
0.0
−0.5
−1.0
0.04
0.08
0.12
0.16

S55

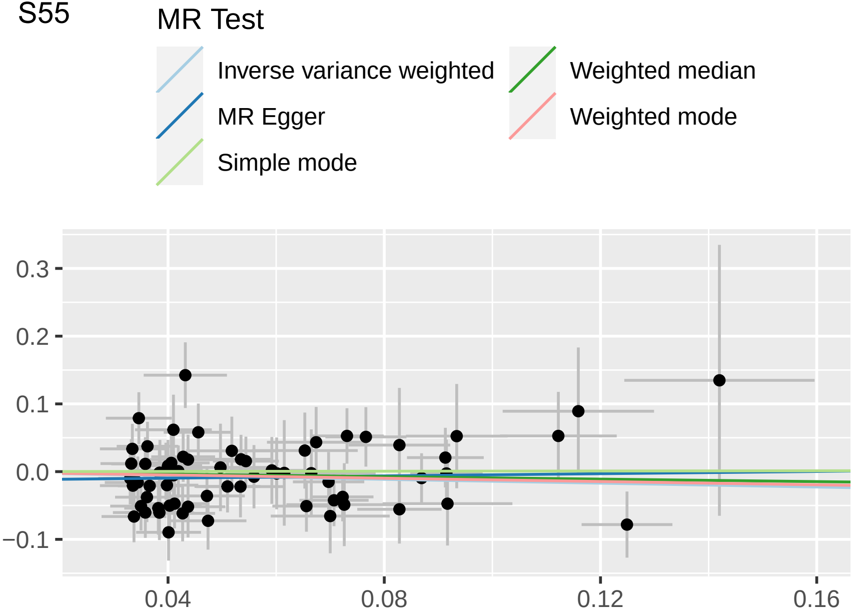

MR Test
Inverse variance weighted
Weighted median
MR Egger
Weighted mode
Simple mode
0.3
0.2
0.1
0.0
−0.1
0.04
0.08
0.12
0.16

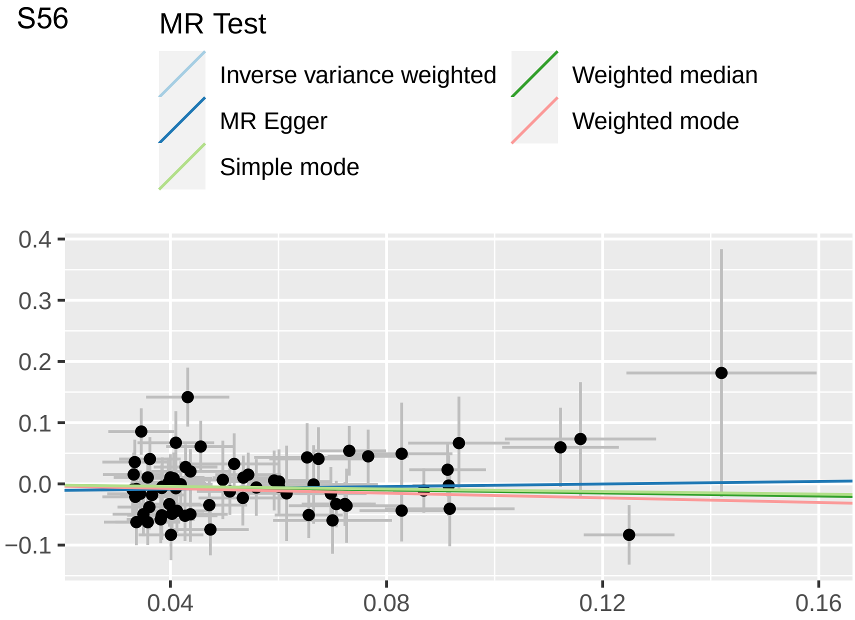
S56
MR Test
Inverse variance weighted
MR Egger
Simple mode
Weighted median
Weighted mode
0.4
0.3
0.2
0.1
0.0
−0.1
0.04
0.08
0.12
0.16
