# Supplementary material for: Mendelian Randomization Combined with Bioinformatics Revealed Specific Allergy-Mediated Protective Mechanisms Against Renal Cell Carcinoma
Source: Phenomics. 2025 Nov 3;5(5):577–89. doi: 10.1007/s43657-025-00229-6 (PMC12881226; doi:10.1007/s43657-025-00229-6)
Supplement: Supplementary file 2 — Supplementary file2 (PDF 1083 KB) [file 43657_2025_229_MOESM2_ESM.pdf]

Additional file 2

Figure S1: Leave-one-out analyses of allergic disease on Acute myeloid leukaemia (all cancers excluded)

Figure S2: Leave-one-out analyses of allergic disease on Acute myeloid leukaemia

Figure S3: Leave-one-out analyses of allergic disease on Brain glioblastoma (all cancers excluded)

Figure S4: Leave-one-out analyses of allergic disease on Brain glioblastoma

Figure S5: Leave-one-out analyses of allergic disease on Diffuse large B-cell lymphoma (all cancers excluded)

Figure S6: Leave-one-out analyses of allergic disease on Diffuse large B-cell lymphoma

Figure S7: Leave-one-out analyses of allergic disease on Follicular lymphoma (all cancers excluded)

Figure S8: Leave-one-out analyses of allergic disease on Follicular lymphoma

Figure S9: Leave-one-out analyses of allergic disease on Hodgkin lymphoma (all cancers excluded)

Figure S10: Leave-one-out analyses of allergic disease on Hodgkin lymphoma

Figure S11: Leave-one-out analyses of allergic disease on Lymphoid leukaemia (all cancers excluded)

Figure S12: Leave-one-out analyses of allergic disease on Lymphoid leukaemia

Figure S13: Leave-one-out analyses of allergic disease on Malignant melanoma of skin (all cancers excluded)

Figure S14: Leave-one-out analyses of allergic disease on Malignant melanoma of skin

Figure S15: Leave-one-out analyses of allergic disease on Malignant neoplasm of anus and anal canal (all cancers excluded)

Figure S16: Leave-one-out analyses of allergic disease on Malignant neoplasm of anus and anal canal

Figure S17: Leave-one-out analyses of allergic disease on Malignant neoplasm of bladder (all cancers excluded)

Figure S18: Leave-one-out analyses of allergic disease on Malignant neoplasm of bladder

Figure S19: Leave-one-out analyses of allergic disease on Malignant neoplasm of bone and articular cartilage (all cancers excluded)

Figure S20: Leave-one-out analyses of allergic disease on Malignant neoplasm of bone and articular cartilage

Figure S21: Leave-one-out analyses of allergic disease on Malignant neoplasm of bronchus and lung (all cancers excluded)

Figure S22: Leave-one-out analyses of allergic disease on Malignant neoplasm of bronchus and lung

Figure S23: Leave-one-out analyses of allergic disease on Malignant neoplasm of colon (all cancers excluded)

Figure S24: Leave-one-out analyses of allergic disease on Malignant neoplasm of colon

Figure S25: Leave-one-out analyses of allergic disease on Malignant neoplasm of eye and adnexa (all cancers excluded)

Figure S26: Leave-one-out analyses of allergic disease on Malignant neoplasm of eye and adnexa

Figure S27: Leave-one-out analyses of allergic disease on Malignant neoplasm of heart, mediastinum and pleura (all cancers excluded)

Figure S28: Leave-one-out analyses of allergic disease on Malignant neoplasm of heart, mediastinum and pleura

Figure S29: Leave-one-out analyses of allergic disease on Malignant neoplasm of kidney, except renal pelvis (all cancers excluded)
Figure S30: Leave-one-out analyses of allergic disease on Malignant neoplasm of kidney, except renal pelvis
Figure S31: Leave-one-out analyses of allergic disease on Malignant neoplasm of larynx (all cancers excluded)
Figure S32: Leave-one-out analyses of allergic disease on Malignant neoplasm of larynx
Figure S33: Leave-one-out analyses of allergic disease on Malignant neoplasm of lip, oral cavity and pharynx (all cancers excluded)
Figure S34: Leave-one-out analyses of allergic disease on Malignant neoplasm of lip, oral cavity and pharynx
Figure S35: Leave-one-out analyses of allergic disease on Malignant neoplasm of liver and intrahepatic bile ducts (all cancers excluded)
Figure S36: Leave-one-out analyses of allergic disease on Malignant neoplasm of liver and intrahepatic bile ducts
Figure S37: Leave-one-out analyses of allergic disease on Malignant neoplasm of meninges (all cancers excluded)
Figure S38: Leave-one-out analyses of allergic disease on Malignant neoplasm of meninges
Figure S39: Leave-one-out analyses of allergic disease on Malignant neoplasm of oesophagus (all cancers excluded)
Figure S40: Leave-one-out analyses of allergic disease on Malignant neoplasm of oesophagus
Figure S41: Leave-one-out analyses of allergic disease on Malignant neoplasm of pancreas (all cancers excluded)
Figure S42: Leave-one-out analyses of allergic disease on Malignant neoplasm of pancreas
Figure S43: Leave-one-out analyses of allergic disease on Malignant neoplasm of rectum (all cancers excluded)
Figure S44: Leave-one-out analyses of allergic disease on Malignant neoplasm of rectum
Figure S45: Leave-one-out analyses of allergic disease on Malignant neoplasm of small intestine (all cancers excluded)
Figure S46: Leave-one-out analyses of allergic disease on Malignant neoplasm of small intestine
Figure S47: Leave-one-out analyses of allergic disease on Malignant neoplasm of stomach (all cancers excluded)
Figure S48: Leave-one-out analyses of allergic disease on Malignant neoplasm of stomach
Figure S49: Leave-one-out analyses of allergic disease on Malignant neoplasm of thyroid gland (all cancers excluded)
Figure S50: Leave-one-out analyses of allergic disease on Malignant neoplasm of thyroid gland
Figure S51: Leave-one-out analyses of allergic disease on Mature T/NK-cell lymphomas (all cancers excluded)
Figure S52: Leave-one-out analyses of allergic disease on Mature T/NK-cell lymphomas
Figure S53: Leave-one-out analyses of allergic disease on Mesothelioma (all cancers excluded)
Figure S54: Leave-one-out analyses of allergic disease on Mesothelioma
Figure S55: Leave-one-out analyses of allergic disease on Non-small cell lung cancer (all cancers excluded)
Figure S56: Leave-one-out analyses of allergic disease on Non-small cell lung cancer

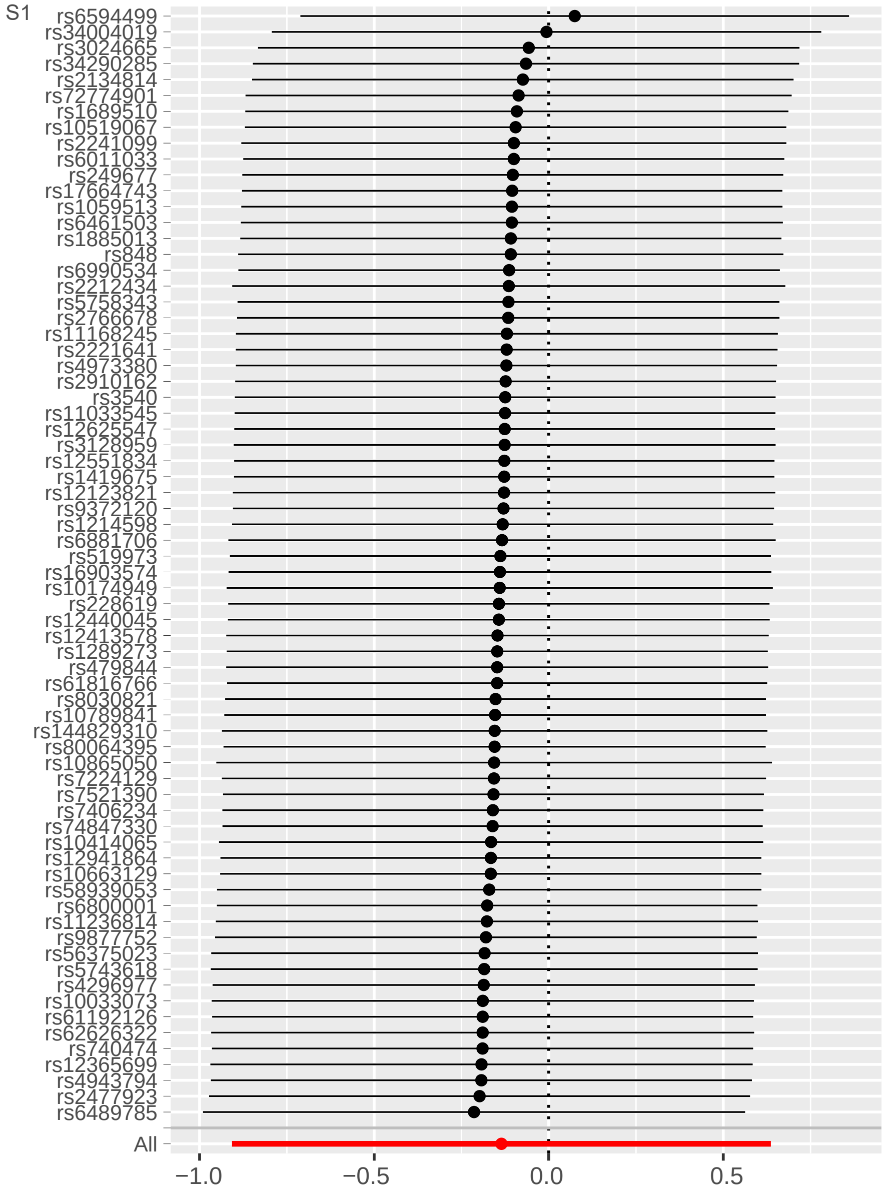

S1
rs6594499
rs34004019
rs3024665
rs34290285
rs2134814
rs72774901
rs1689510
rs10519067
rs2241099
rs6011033
rs249677
rs17664743
rs1059513
rs6461503
rs1885013
rs848
rs6990534
rs2212434
rs5758343
rs2766678
rs11168245
rs2221641
rs4973380
rs2910162
rs3540
rs11033545
rs12625547
rs3128959
rs12551834
rs1419675
rs12123821
rs9372120
rs1214598
rs6881706
rs519973
rs16903574
rs10174949
rs228619
rs12440045
rs12413578
rs1289273
rs479844
rs61816766
rs8030821
rs10789841
rs144829310
rs80064395
rs10865050
rs7224129
rs7521390
rs7406234
rs74847330
rs10414065
rs12941864
rs10663129
rs58939053
rs6800001
rs11236814
rs9877752
rs56375023
rs5743618
rs4296977
rs10033073
rs61192126
rs62626322
rs740474
rs12365699
rs4943794
rs2477923
rs6489785
All
−1.0
−0.5
0.0
0.5

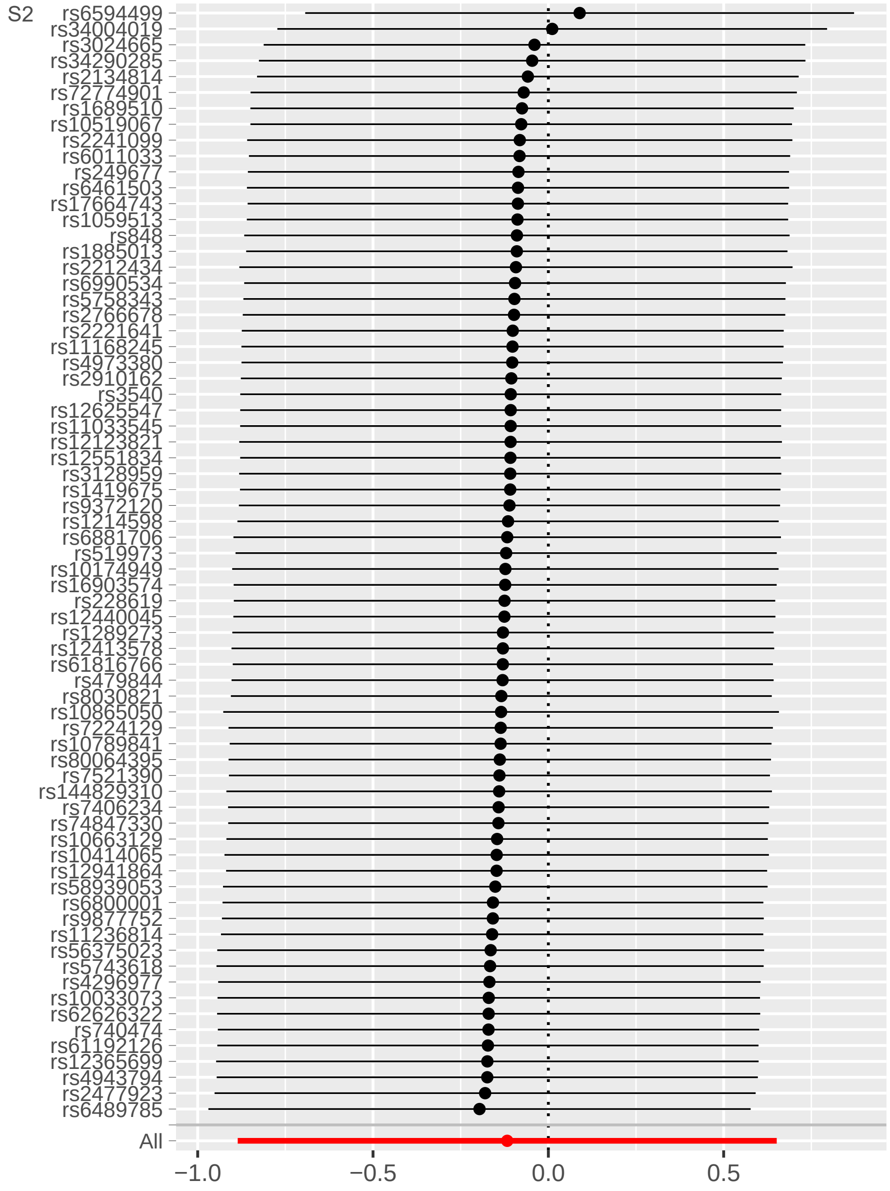

S2
rs6594499
rs34004019
rs3024665
rs34290285
rs2134814
rs72774901
rs1689510
rs10519067
rs2241099
rs6011033
rs249677
rs6461503
rs17664743
rs1059513
rs848
rs1885013
rs2212434
rs6990534
rs5758343
rs2766678
rs2221641
rs11168245
rs4973380
rs2910162
rs3540
rs12625547
rs11033545
rs12123821
rs12551834
rs3128959
rs1419675
rs9372120
rs1214598
rs6881706
rs519973
rs10174949
rs16903574
rs228619
rs12440045
rs1289273
rs12413578
rs61816766
rs479844
rs8030821
rs10865050
rs7224129
rs10789841
rs80064395
rs7521390
rs144829310
rs7406234
rs74847330
rs10663129
rs10414065
rs12941864
rs58939053
rs6800001
rs9877752
rs11236814
rs56375023
rs5743618
rs4296977
rs10033073
rs62626322
rs740474
rs61192126
rs12365699
rs4943794
rs2477923
rs6489785
All
−1.0
−0.5
0.0
0.5

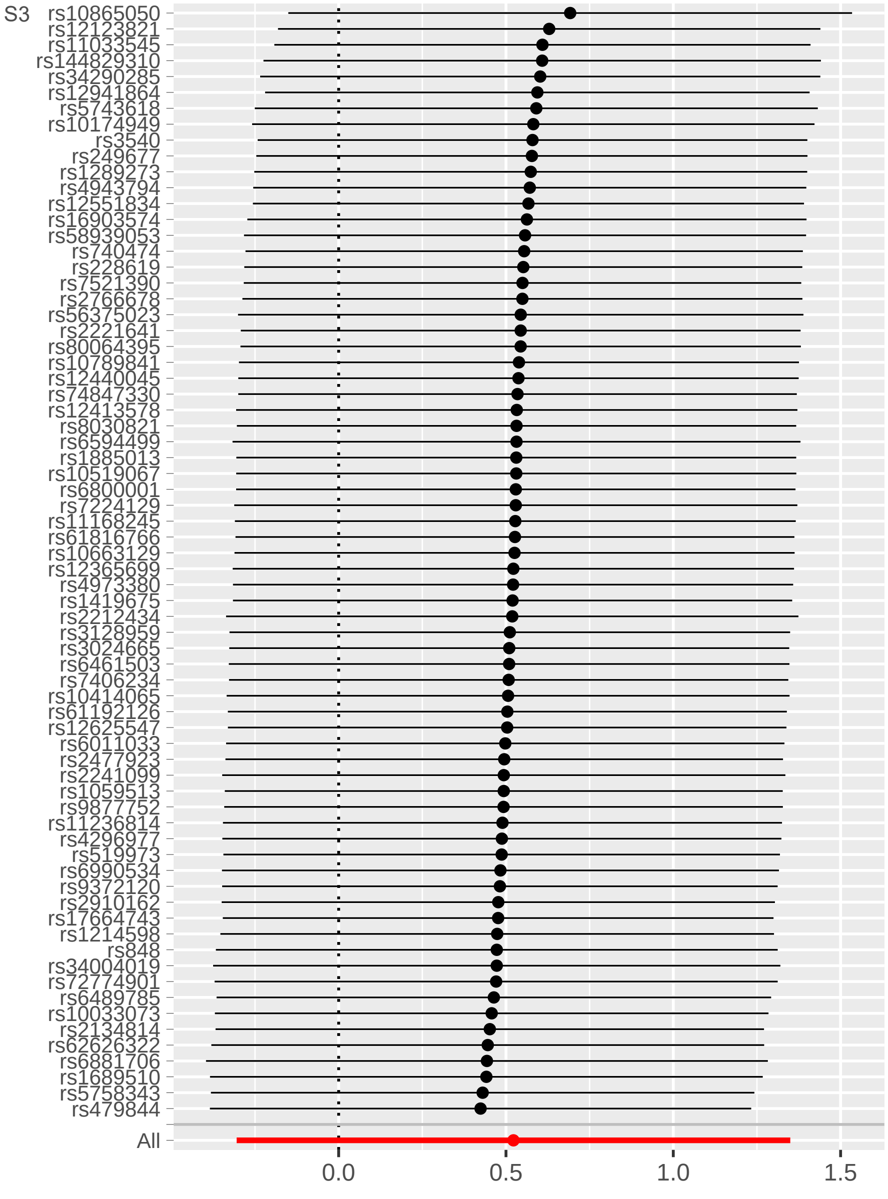
S3
rs10865050
rs12123821
rs11033545
rs144829310
rs34290285
rs12941864
rs5743618
rs10174949
rs3540
rs249677
rs1289273
rs4943794
rs12551834
rs16903574
rs58939053
rs740474
rs228619
rs7521390
rs2766678
rs56375023
rs2221641
rs80064395
rs10789841
rs12440045
rs74847330
rs12413578
rs8030821
rs6594499
rs1885013
rs10519067
rs6800001
rs7224129
rs11168245
rs61816766
rs10663129
rs12365699
rs4973380
rs1419675
rs2212434
rs3128959
rs3024665
rs6461503
rs7406234
rs10414065
rs61192126
rs12625547
rs6011033
rs2477923
rs2241099
rs1059513
rs9877752
rs11236814
rs4296977
rs519973
rs6990534
rs9372120
rs2910162
rs17664743
rs1214598
rs848
rs34004019
rs72774901
rs6489785
rs10033073
rs2134814
rs62626322
rs6881706
rs1689510
rs5758343
rs479844
All
0.0
0.5
1.0
1.5

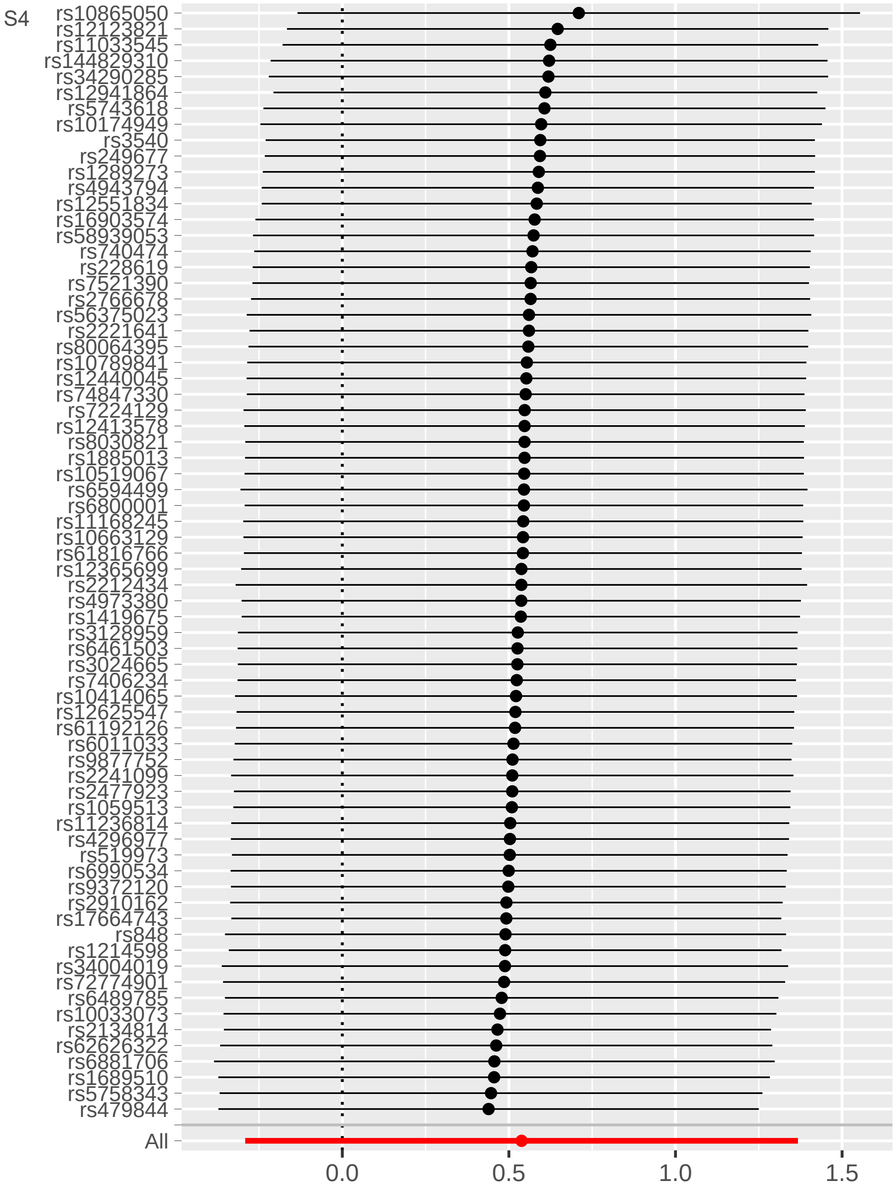
S4
rs10865050
rs12123821
rs11033545
rs144829310
rs34290285
rs12941864
rs5743618
rs10174949
rs3540
rs249677
rs1289273
rs4943794
rs12551834
rs16903574
rs58939053
rs740474
rs228619
rs7521390
rs2766678
rs56375023
rs2221641
rs80064395
rs10789841
rs12440045
rs74847330
rs7224129
rs12413578
rs8030821
rs1885013
rs10519067
rs6594499
rs6800001
rs11168245
rs10663129
rs61816766
rs12365699
rs2212434
rs4973380
rs1419675
rs3128959
rs6461503
rs3024665
rs7406234
rs10414065
rs12625547
rs61192126
rs6011033
rs9877752
rs2241099
rs2477923
rs1059513
rs11236814
rs4296977
rs519973
rs6990534
rs9372120
rs2910162
rs17664743
rs848
rs1214598
rs34004019
rs72774901
rs6489785
rs10033073
rs2134814
rs62626322
rs6881706
rs1689510
rs5758343
rs479844
All
0.0
0.5
1.0
1.5

S5

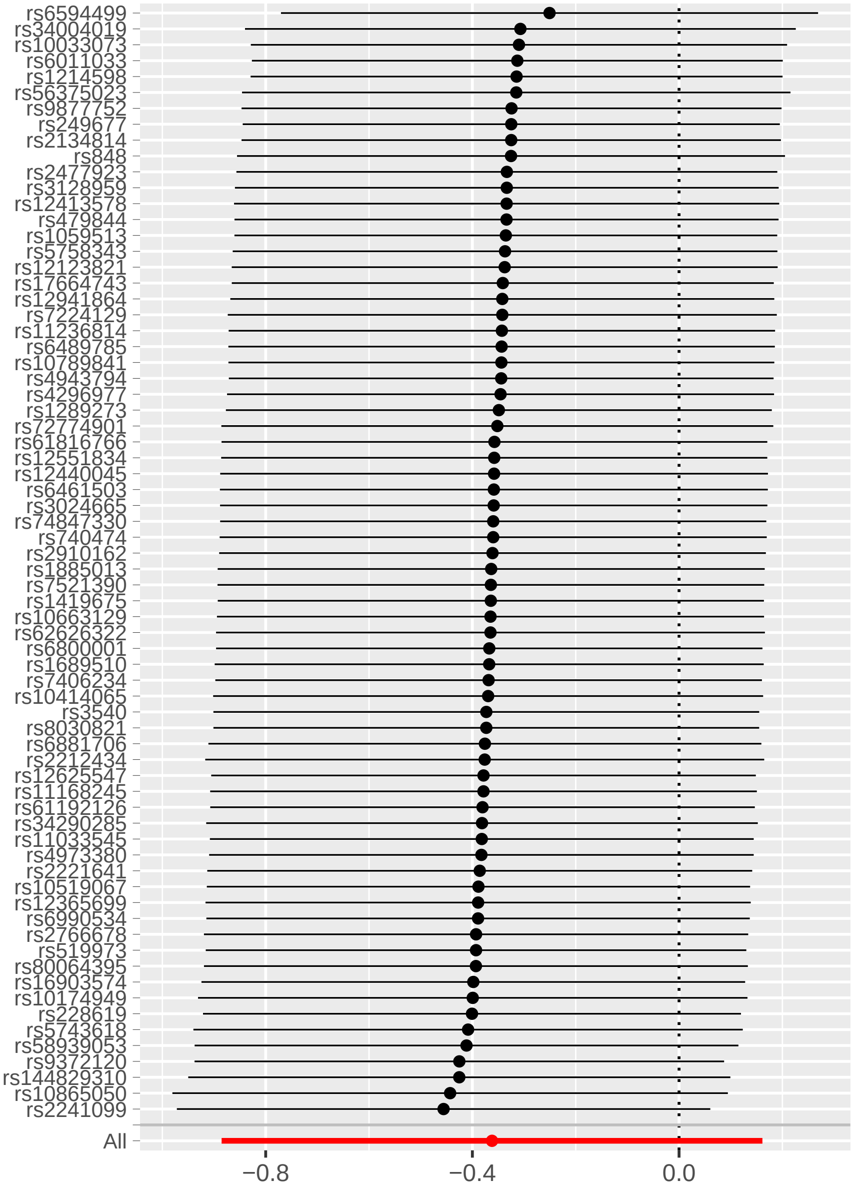

rs6594499
rs34004019
rs10033073
rs6011033
rs1214598
rs56375023
rs9877752
rs249677
rs2134814
rs848
rs2477923
rs3128959
rs12413578
rs479844
rs1059513
rs5758343
rs12123821
rs17664743
rs12941864
rs7224129
rs11236814
rs6489785
rs10789841
rs4943794
rs4296977
rs1289273
rs72774901
rs61816766
rs12551834
rs12440045
rs6461503
rs3024665
rs74847330
rs740474
rs2910162
rs1885013
rs7521390
rs1419675
rs10663129
rs62626322
rs6800001
rs1689510
rs7406234
rs10414065
rs3540
rs8030821
rs6881706
rs2212434
rs12625547
rs11168245
rs61192126
rs34290285
rs11033545
rs4973380
rs2221641
rs10519067
rs12365699
rs6990534
rs2766678
rs519973
rs80064395
rs16903574
rs10174949
rs228619
rs5743618
rs58939053
rs9372120
rs144829310
rs10865050
rs2241099
All
−0.8
−0.4
0.0

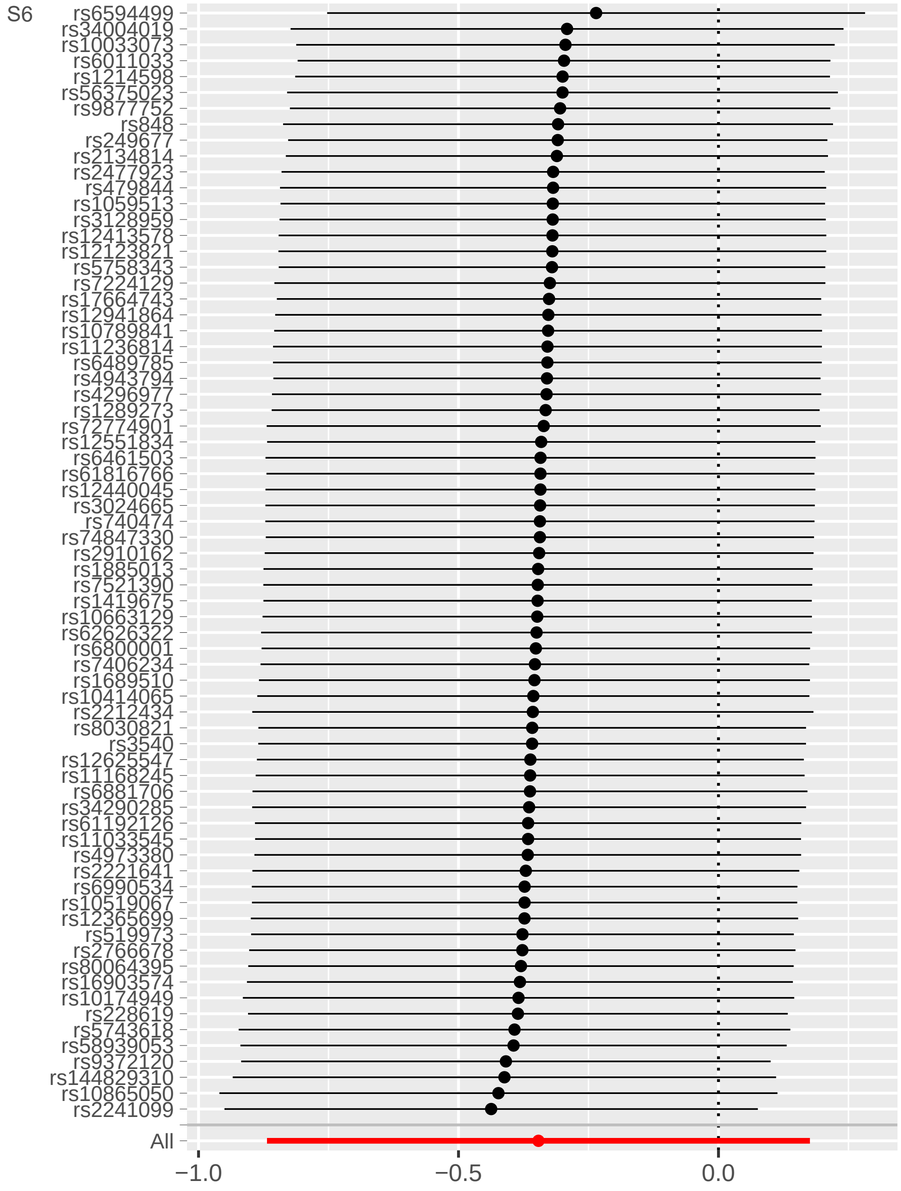

S6
rs6594499
rs34004019
rs10033073
rs6011033
rs1214598
rs56375023
rs9877752
rs848
rs249677
rs2134814
rs2477923
rs479844
rs1059513
rs3128959
rs12413578
rs12123821
rs5758343
rs7224129
rs17664743
rs12941864
rs10789841
rs11236814
rs6489785
rs4943794
rs4296977
rs1289273
rs72774901
rs12551834
rs6461503
rs61816766
rs12440045
rs3024665
rs740474
rs74847330
rs2910162
rs1885013
rs7521390
rs1419675
rs10663129
rs62626322
rs6800001
rs7406234
rs1689510
rs10414065
rs2212434
rs8030821
rs3540
rs12625547
rs11168245
rs6881706
rs34290285
rs61192126
rs11033545
rs4973380
rs2221641
rs6990534
rs10519067
rs12365699
rs519973
rs2766678
rs80064395
rs16903574
rs10174949
rs228619
rs5743618
rs58939053
rs9372120
rs144829310
rs10865050
rs2241099
All
−1.0
−0.5
0.0

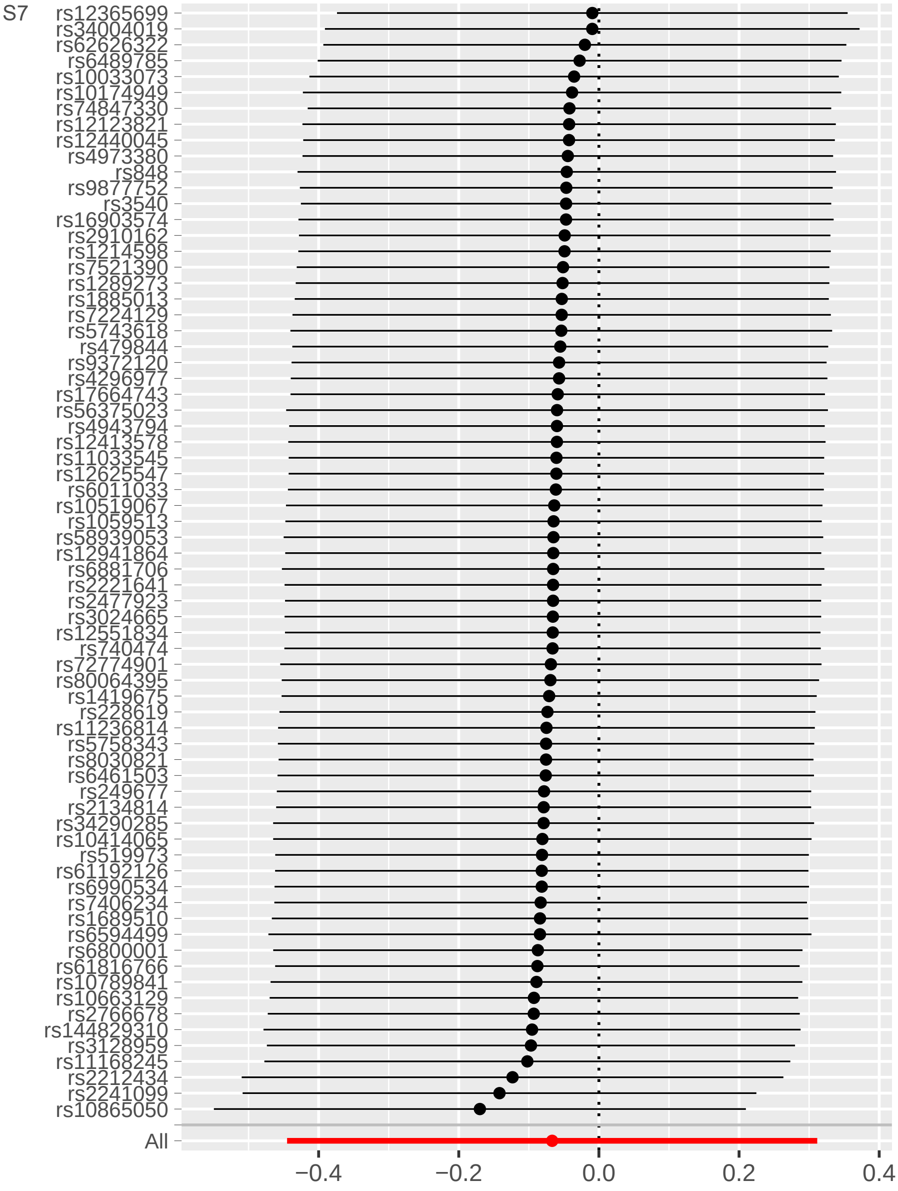
S7
rs12365699
rs34004019
rs62626322
rs6489785
rs10033073
rs10174949
rs74847330
rs12123821
rs12440045
rs4973380
rs848
rs9877752
rs3540
rs16903574
rs2910162
rs1214598
rs7521390
rs1289273
rs1885013
rs7224129
rs5743618
rs479844
rs9372120
rs4296977
rs17664743
rs56375023
rs4943794
rs12413578
rs11033545
rs12625547
rs6011033
rs10519067
rs1059513
rs58939053
rs12941864
rs6881706
rs2221641
rs2477923
rs3024665
rs12551834
rs740474
rs72774901
rs80064395
rs1419675
rs228619
rs11236814
rs5758343
rs8030821
rs6461503
rs249677
rs2134814
rs34290285
rs10414065
rs519973
rs61192126
rs6990534
rs7406234
rs1689510
rs6594499
rs6800001
rs61816766
rs10789841
rs10663129
rs2766678
rs144829310
rs3128959
rs11168245
rs2212434
rs2241099
rs10865050
All
−0.4
−0.2
0.0
0.2
0.4

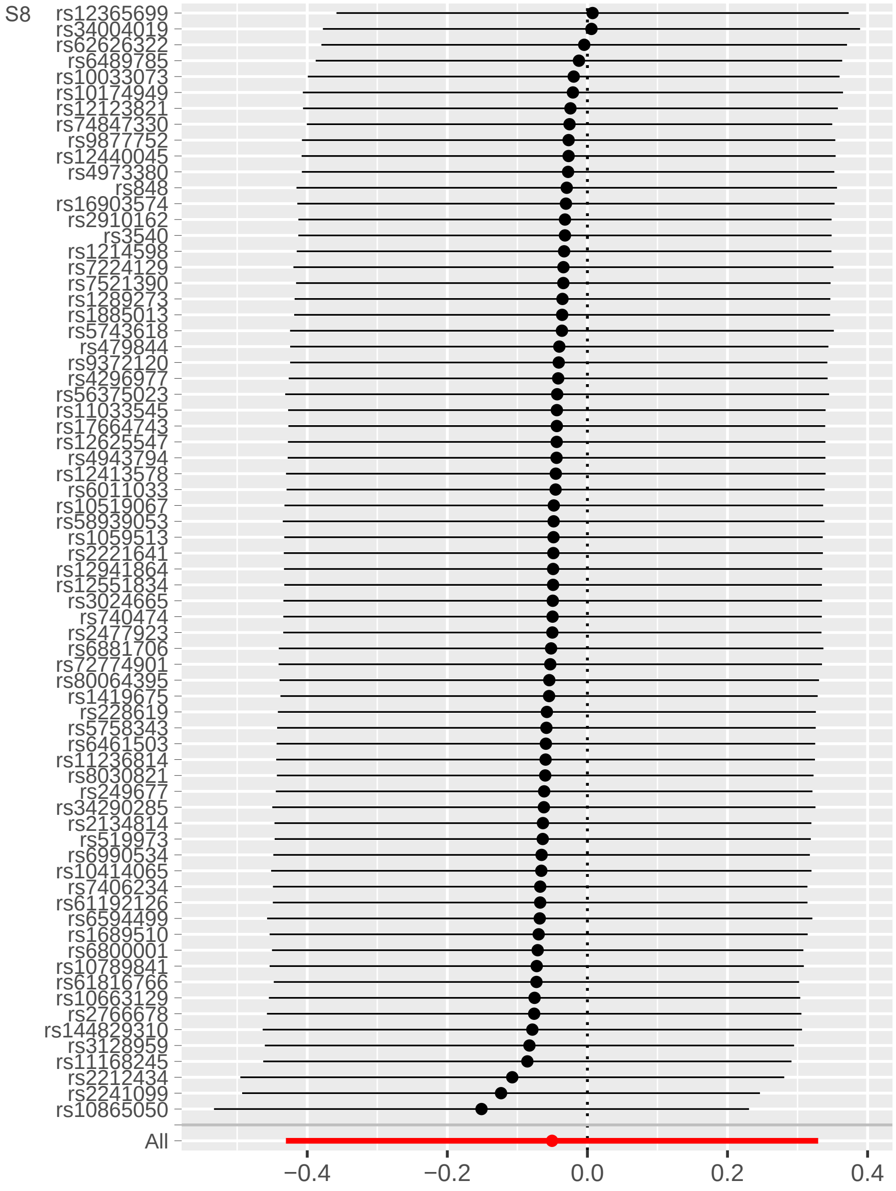

S8
rs12365699
rs34004019
rs62626322
rs6489785
rs10033073
rs10174949
rs12123821
rs74847330
rs9877752
rs12440045
rs4973380
rs848
rs16903574
rs2910162
rs3540
rs1214598
rs7224129
rs7521390
rs1289273
rs1885013
rs5743618
rs479844
rs9372120
rs4296977
rs56375023
rs11033545
rs17664743
rs12625547
rs4943794
rs12413578
rs6011033
rs10519067
rs58939053
rs1059513
rs2221641
rs12941864
rs12551834
rs3024665
rs740474
rs2477923
rs6881706
rs72774901
rs80064395
rs1419675
rs228619
rs5758343
rs6461503
rs11236814
rs8030821
rs249677
rs34290285
rs2134814
rs519973
rs6990534
rs10414065
rs7406234
rs61192126
rs6594499
rs1689510
rs6800001
rs10789841
rs61816766
rs10663129
rs2766678
rs144829310
rs3128959
rs11168245
rs2212434
rs2241099
rs10865050
All
−0.4
−0.2
0.0
0.2
0.4

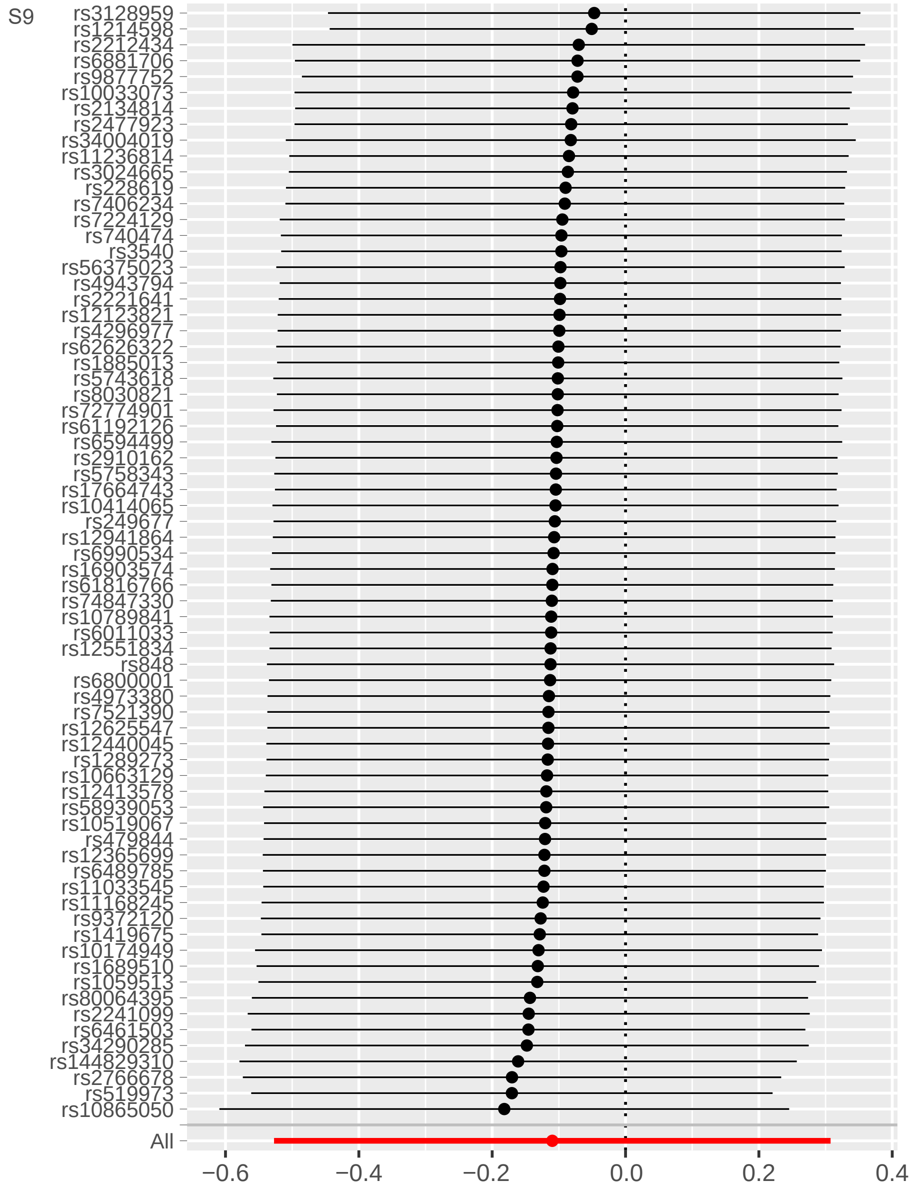
S9
rs3128959
rs1214598
rs2212434
rs6881706
rs9877752
rs10033073
rs2134814
rs2477923
rs34004019
rs11236814
rs3024665
rs228619
rs7406234
rs7224129
rs740474
rs3540
rs56375023
rs4943794
rs2221641
rs12123821
rs4296977
rs62626322
rs1885013
rs5743618
rs8030821
rs72774901
rs61192126
rs6594499
rs2910162
rs5758343
rs17664743
rs10414065
rs249677
rs12941864
rs6990534
rs16903574
rs61816766
rs74847330
rs10789841
rs6011033
rs12551834
rs848
rs6800001
rs4973380
rs7521390
rs12625547
rs12440045
rs1289273
rs10663129
rs12413578
rs58939053
rs10519067
rs479844
rs12365699
rs6489785
rs11033545
rs11168245
rs9372120
rs1419675
rs10174949
rs1689510
rs1059513
rs80064395
rs2241099
rs6461503
rs34290285
rs144829310
rs2766678
rs519973
rs10865050
All
−0.6
−0.4
−0.2
0.0
0.2
0.4

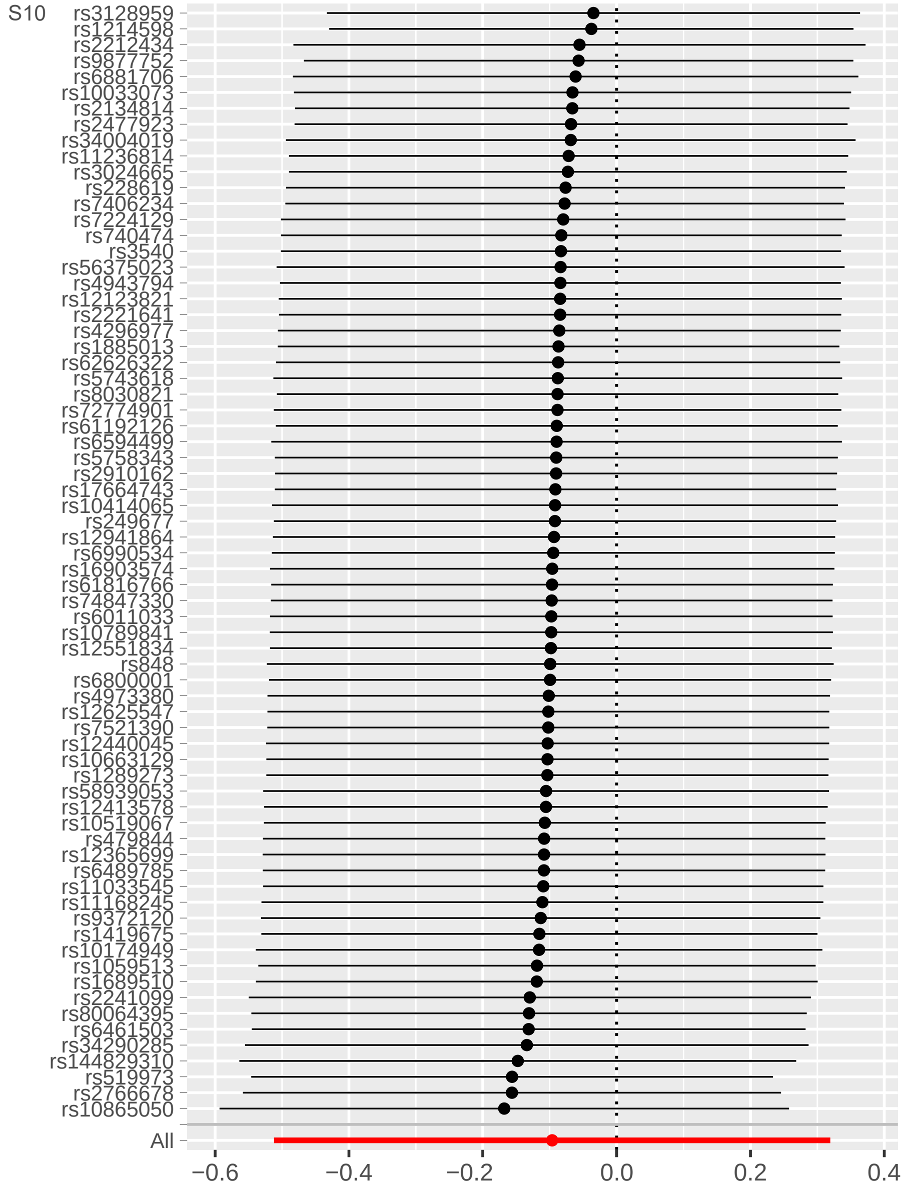

S10
rs3128959
rs1214598
rs2212434
rs9877752
rs6881706
rs10033073
rs2134814
rs2477923
rs34004019
rs11236814
rs3024665
rs228619
rs7406234
rs7224129
rs740474
rs3540
rs56375023
rs4943794
rs12123821
rs2221641
rs4296977
rs1885013
rs62626322
rs5743618
rs8030821
rs72774901
rs61192126
rs6594499
rs5758343
rs2910162
rs17664743
rs10414065
rs249677
rs12941864
rs6990534
rs16903574
rs61816766
rs74847330
rs6011033
rs10789841
rs12551834
rs848
rs6800001
rs4973380
rs12625547
rs7521390
rs12440045
rs10663129
rs1289273
rs58939053
rs12413578
rs10519067
rs479844
rs12365699
rs6489785
rs11033545
rs11168245
rs9372120
rs1419675
rs10174949
rs1059513
rs1689510
rs2241099
rs80064395
rs6461503
rs34290285
rs144829310
rs519973
rs2766678
rs10865050
All
−0.6
−0.4
−0.2
0.0
0.2
0.4

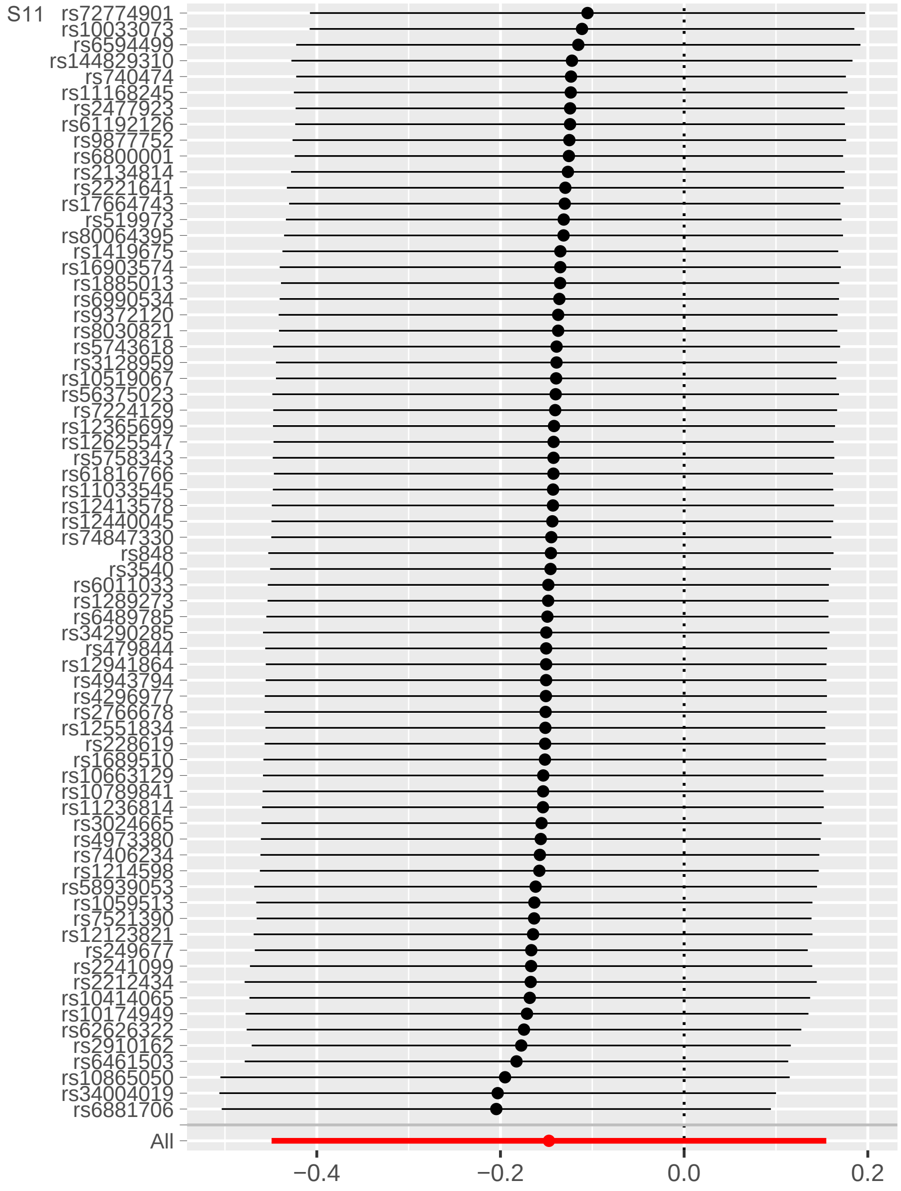

S11
rs72774901
rs10033073
rs6594499
rs144829310
rs740474
rs11168245
rs2477923
rs61192126
rs9877752
rs6800001
rs2134814
rs2221641
rs17664743
rs519973
rs80064395
rs1419675
rs16903574
rs1885013
rs6990534
rs9372120
rs8030821
rs5743618
rs3128959
rs10519067
rs56375023
rs7224129
rs12365699
rs12625547
rs5758343
rs61816766
rs11033545
rs12413578
rs12440045
rs74847330
rs848
rs3540
rs6011033
rs1289273
rs6489785
rs34290285
rs479844
rs12941864
rs4943794
rs4296977
rs2766678
rs12551834
rs228619
rs1689510
rs10663129
rs10789841
rs11236814
rs3024665
rs4973380
rs7406234
rs1214598
rs58939053
rs1059513
rs7521390
rs12123821
rs249677
rs2241099
rs2212434
rs10414065
rs10174949
rs62626322
rs2910162
rs6461503
rs10865050
rs34004019
rs6881706
All
−0.4
−0.2
0.0
0.2

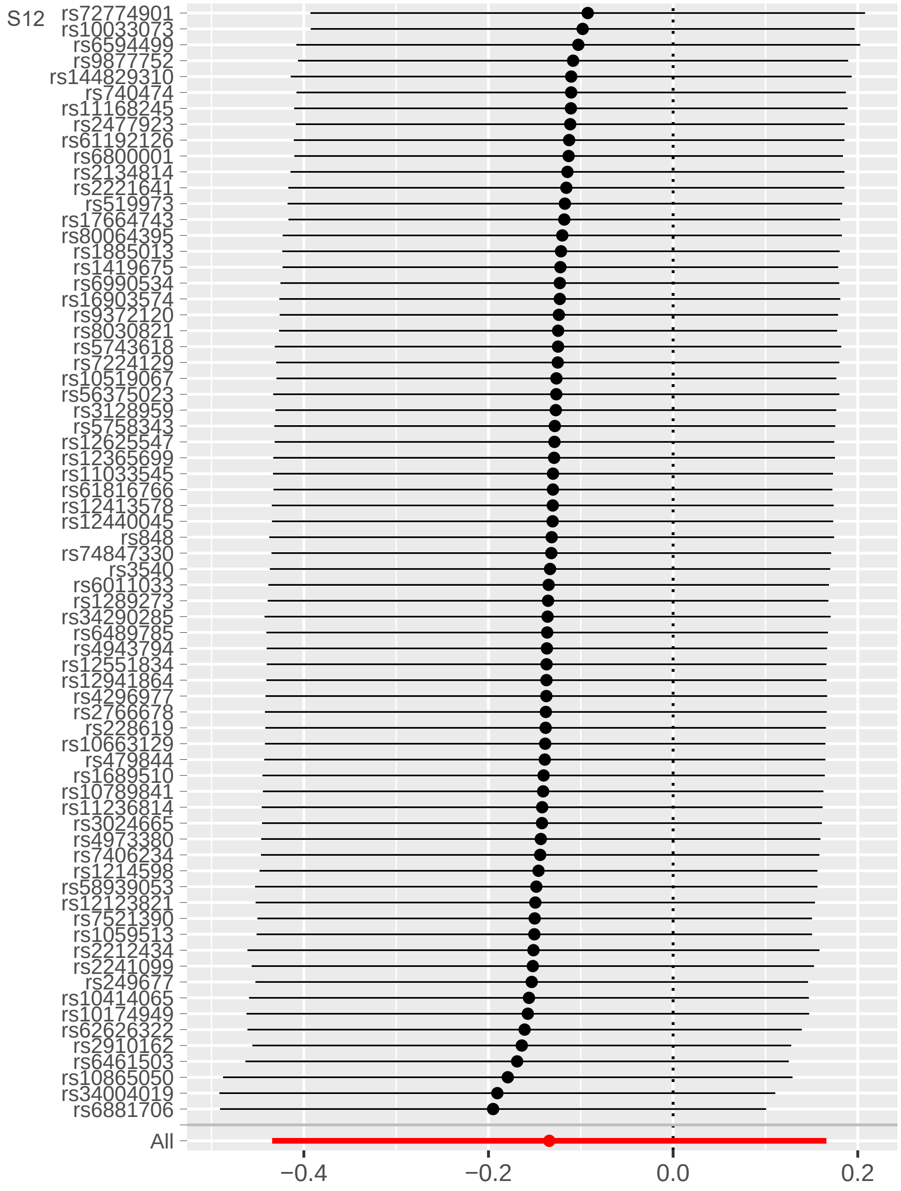
S12
rs72774901
rs10033073
rs6594499
rs9877752
rs144829310
rs740474
rs11168245
rs2477923
rs61192126
rs6800001
rs2134814
rs2221641
rs519973
rs17664743
rs80064395
rs1885013
rs1419675
rs6990534
rs16903574
rs9372120
rs8030821
rs5743618
rs7224129
rs10519067
rs56375023
rs3128959
rs5758343
rs12625547
rs12365699
rs11033545
rs61816766
rs12413578
rs12440045
rs848
rs74847330
rs3540
rs6011033
rs1289273
rs34290285
rs6489785
rs4943794
rs12551834
rs12941864
rs4296977
rs2766678
rs228619
rs10663129
rs479844
rs1689510
rs10789841
rs11236814
rs3024665
rs4973380
rs7406234
rs1214598
rs58939053
rs12123821
rs7521390
rs1059513
rs2212434
rs2241099
rs249677
rs10414065
rs10174949
rs62626322
rs2910162
rs6461503
rs10865050
rs34004019
rs6881706
All
−0.4
−0.2
0.0
0.2

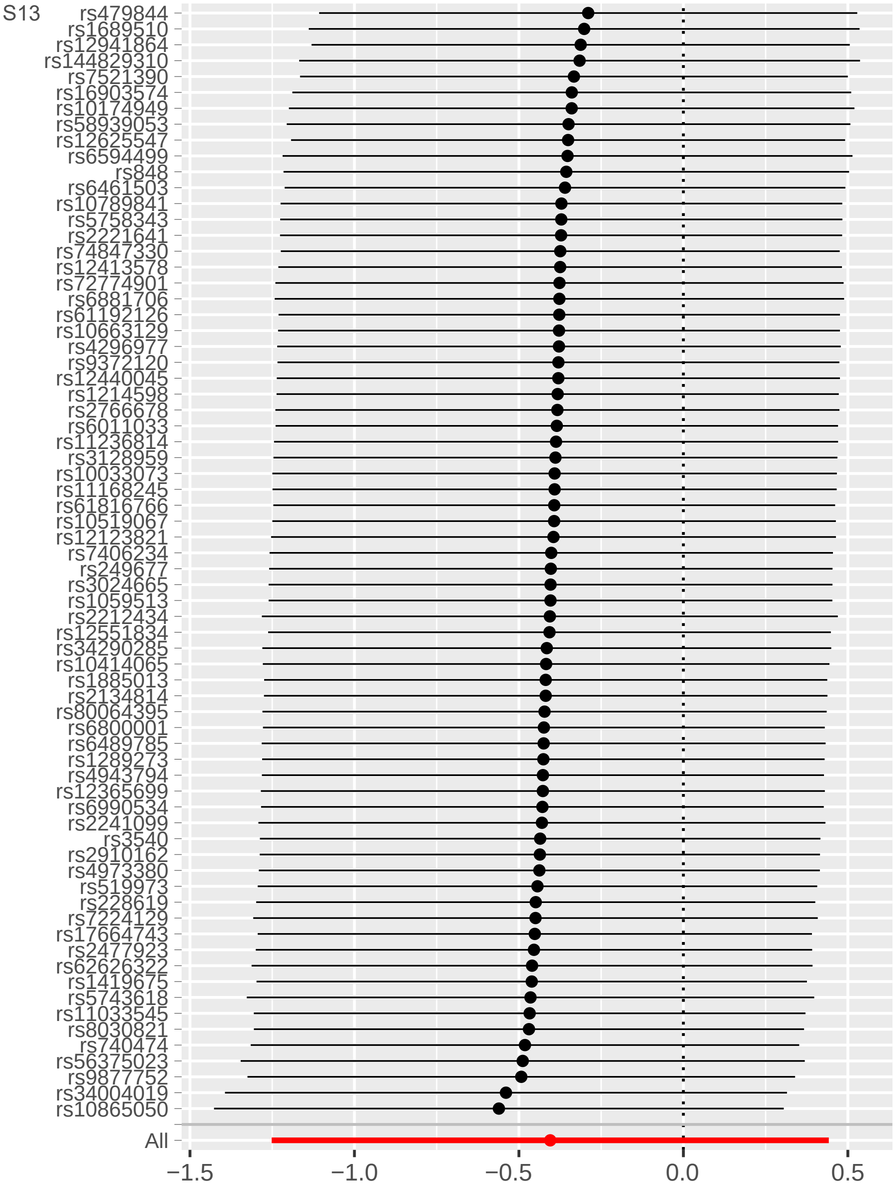

S13
rs479844
rs1689510
rs12941864
rs144829310
rs7521390
rs16903574
rs10174949
rs58939053
rs12625547
rs6594499
rs848
rs6461503
rs10789841
rs5758343
rs2221641
rs74847330
rs12413578
rs72774901
rs6881706
rs61192126
rs10663129
rs4296977
rs9372120
rs12440045
rs1214598
rs2766678
rs6011033
rs11236814
rs3128959
rs10033073
rs11168245
rs61816766
rs10519067
rs12123821
rs7406234
rs249677
rs3024665
rs1059513
rs2212434
rs12551834
rs34290285
rs10414065
rs1885013
rs2134814
rs80064395
rs6800001
rs6489785
rs1289273
rs4943794
rs12365699
rs6990534
rs2241099
rs3540
rs2910162
rs4973380
rs519973
rs228619
rs7224129
rs17664743
rs2477923
rs62626322
rs1419675
rs5743618
rs11033545
rs8030821
rs740474
rs56375023
rs9877752
rs34004019
rs10865050
All
−1.5
−1.0
−0.5
0.0
0.5

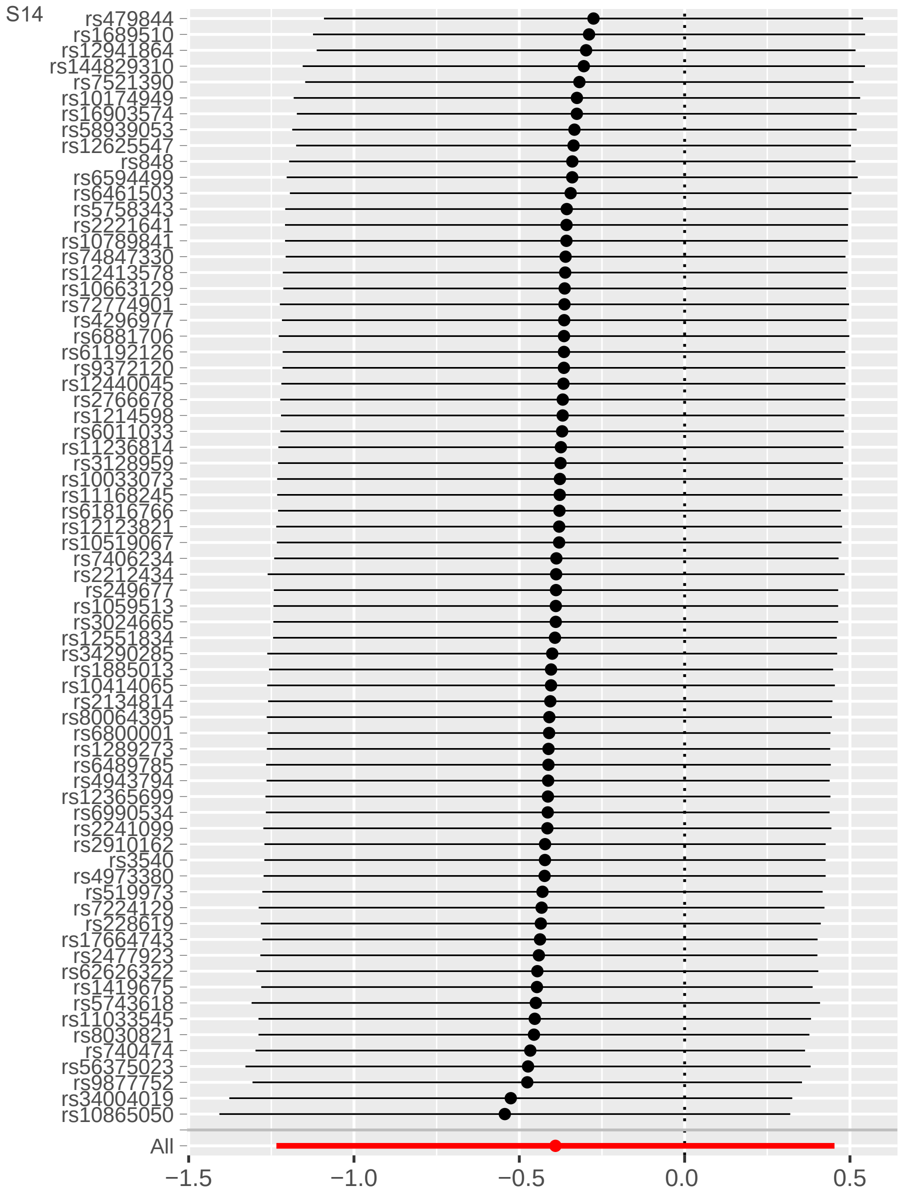

S14
rs479844
rs1689510
rs12941864
rs144829310
rs7521390
rs10174949
rs16903574
rs58939053
rs12625547
rs848
rs6594499
rs6461503
rs5758343
rs2221641
rs10789841
rs74847330
rs12413578
rs10663129
rs72774901
rs4296977
rs6881706
rs61192126
rs9372120
rs12440045
rs2766678
rs1214598
rs6011033
rs11236814
rs3128959
rs10033073
rs11168245
rs61816766
rs12123821
rs10519067
rs7406234
rs2212434
rs249677
rs1059513
rs3024665
rs12551834
rs34290285
rs1885013
rs10414065
rs2134814
rs80064395
rs6800001
rs1289273
rs6489785
rs4943794
rs12365699
rs6990534
rs2241099
rs2910162
rs3540
rs4973380
rs519973
rs7224129
rs228619
rs17664743
rs2477923
rs62626322
rs1419675
rs5743618
rs11033545
rs8030821
rs740474
rs56375023
rs9877752
rs34004019
rs10865050
All
−1.5
−1.0
−0.5
0.0
0.5

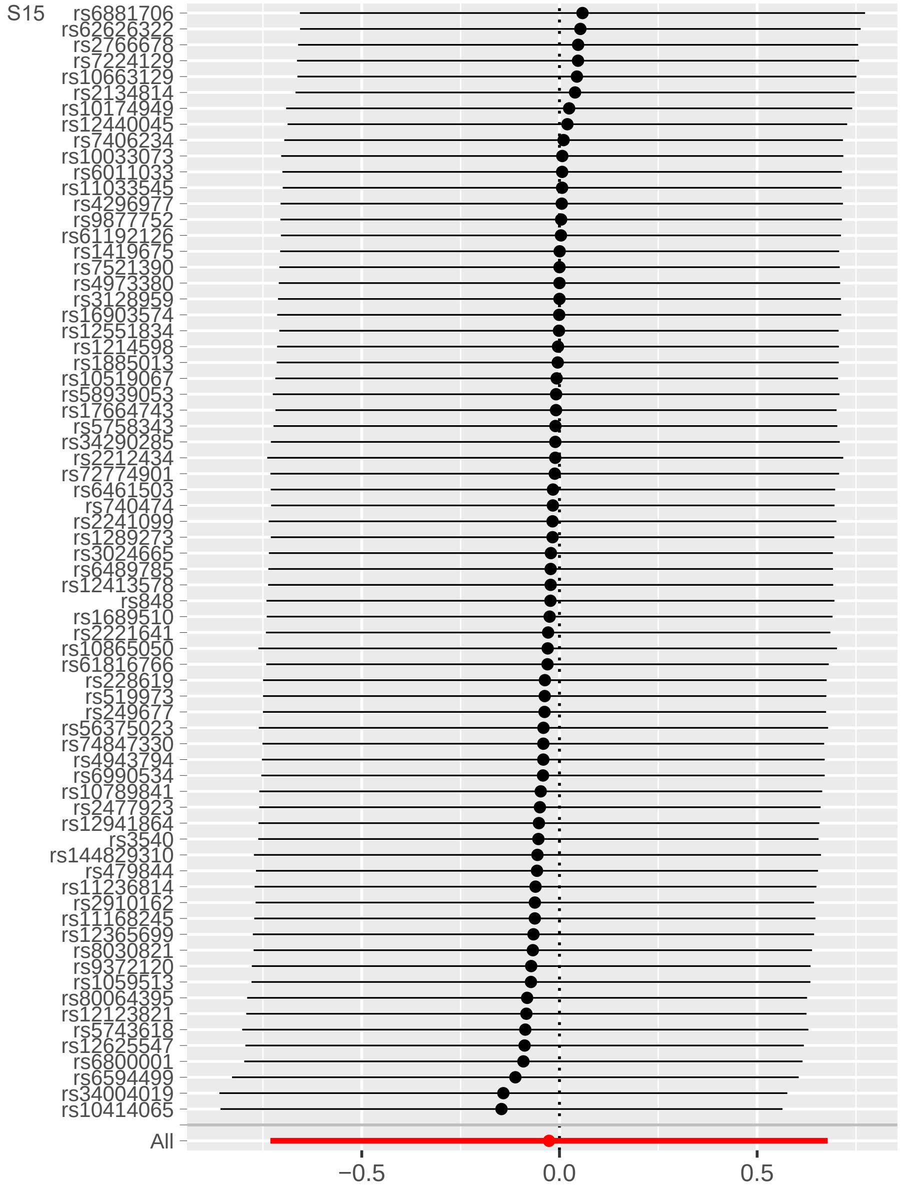
S15
rs6881706
rs62626322
rs2766678
rs7224129
rs10663129
rs2134814
rs10174949
rs12440045
rs7406234
rs10033073
rs6011033
rs11033545
rs4296977
rs9877752
rs61192126
rs1419675
rs7521390
rs4973380
rs3128959
rs16903574
rs12551834
rs1214598
rs1885013
rs10519067
rs58939053
rs17664743
rs5758343
rs34290285
rs2212434
rs72774901
rs6461503
rs740474
rs2241099
rs1289273
rs3024665
rs6489785
rs12413578
rs848
rs1689510
rs2221641
rs10865050
rs61816766
rs228619
rs519973
rs249677
rs56375023
rs74847330
rs4943794
rs6990534
rs10789841
rs2477923
rs12941864
rs3540
rs144829310
rs479844
rs11236814
rs2910162
rs11168245
rs12365699
rs8030821
rs9372120
rs1059513
rs80064395
rs12123821
rs5743618
rs12625547
rs6800001
rs6594499
rs34004019
rs10414065
All
−0.5
0.0
0.5

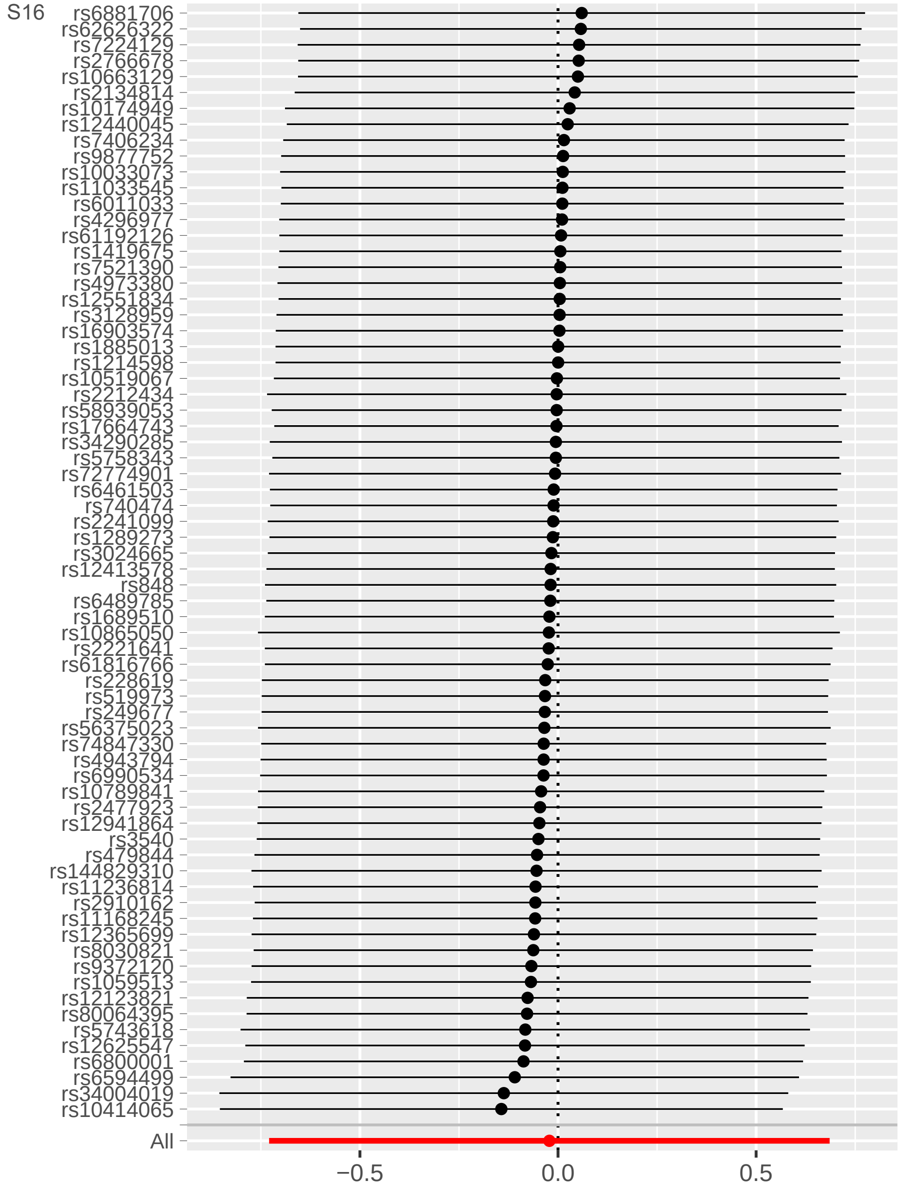
S16
rs6881706
rs62626322
rs7224129
rs2766678
rs10663129
rs2134814
rs10174949
rs12440045
rs7406234
rs9877752
rs10033073
rs11033545
rs6011033
rs4296977
rs61192126
rs1419675
rs7521390
rs4973380
rs12551834
rs3128959
rs16903574
rs1885013
rs1214598
rs10519067
rs2212434
rs58939053
rs17664743
rs34290285
rs5758343
rs72774901
rs6461503
rs740474
rs2241099
rs1289273
rs3024665
rs12413578
rs848
rs6489785
rs1689510
rs10865050
rs2221641
rs61816766
rs228619
rs519973
rs249677
rs56375023
rs74847330
rs4943794
rs6990534
rs10789841
rs2477923
rs12941864
rs3540
rs479844
rs144829310
rs11236814
rs2910162
rs11168245
rs12365699
rs8030821
rs9372120
rs1059513
rs12123821
rs80064395
rs5743618
rs12625547
rs6800001
rs6594499
rs34004019
rs10414065
All
−0.5
0.0
0.5

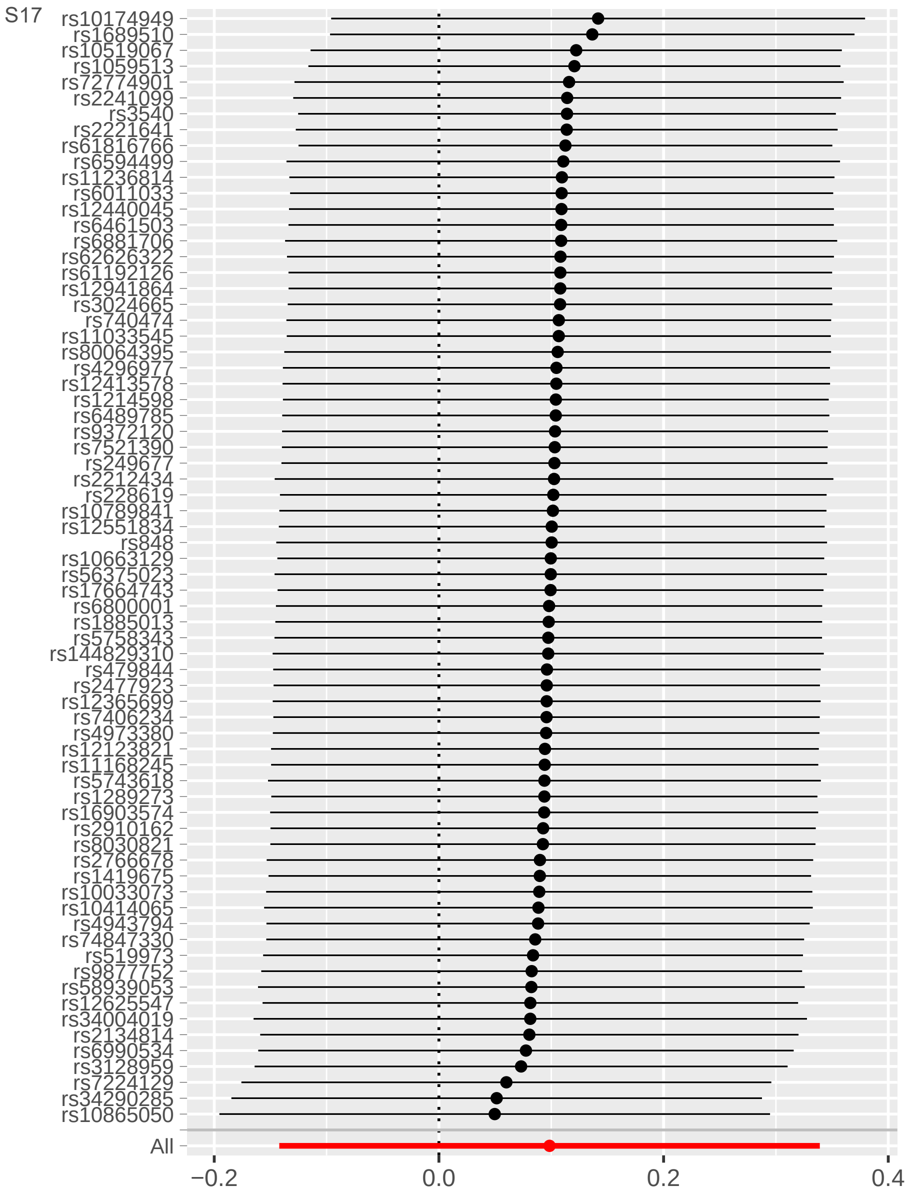
S17
rs10174949
rs1689510
rs10519067
rs1059513
rs72774901
rs2241099
rs3540
rs2221641
rs61816766
rs6594499
rs11236814
rs6011033
rs12440045
rs6461503
rs6881706
rs62626322
rs61192126
rs12941864
rs3024665
rs740474
rs11033545
rs80064395
rs4296977
rs12413578
rs1214598
rs6489785
rs9372120
rs7521390
rs249677
rs2212434
rs228619
rs10789841
rs12551834
rs848
rs10663129
rs56375023
rs17664743
rs6800001
rs1885013
rs5758343
rs144829310
rs479844
rs2477923
rs12365699
rs7406234
rs4973380
rs12123821
rs11168245
rs5743618
rs1289273
rs16903574
rs2910162
rs8030821
rs2766678
rs1419675
rs10033073
rs10414065
rs4943794
rs74847330
rs519973
rs9877752
rs58939053
rs12625547
rs34004019
rs2134814
rs6990534
rs3128959
rs7224129
rs34290285
rs10865050
All
−0.2
0.0
0.2
0.4

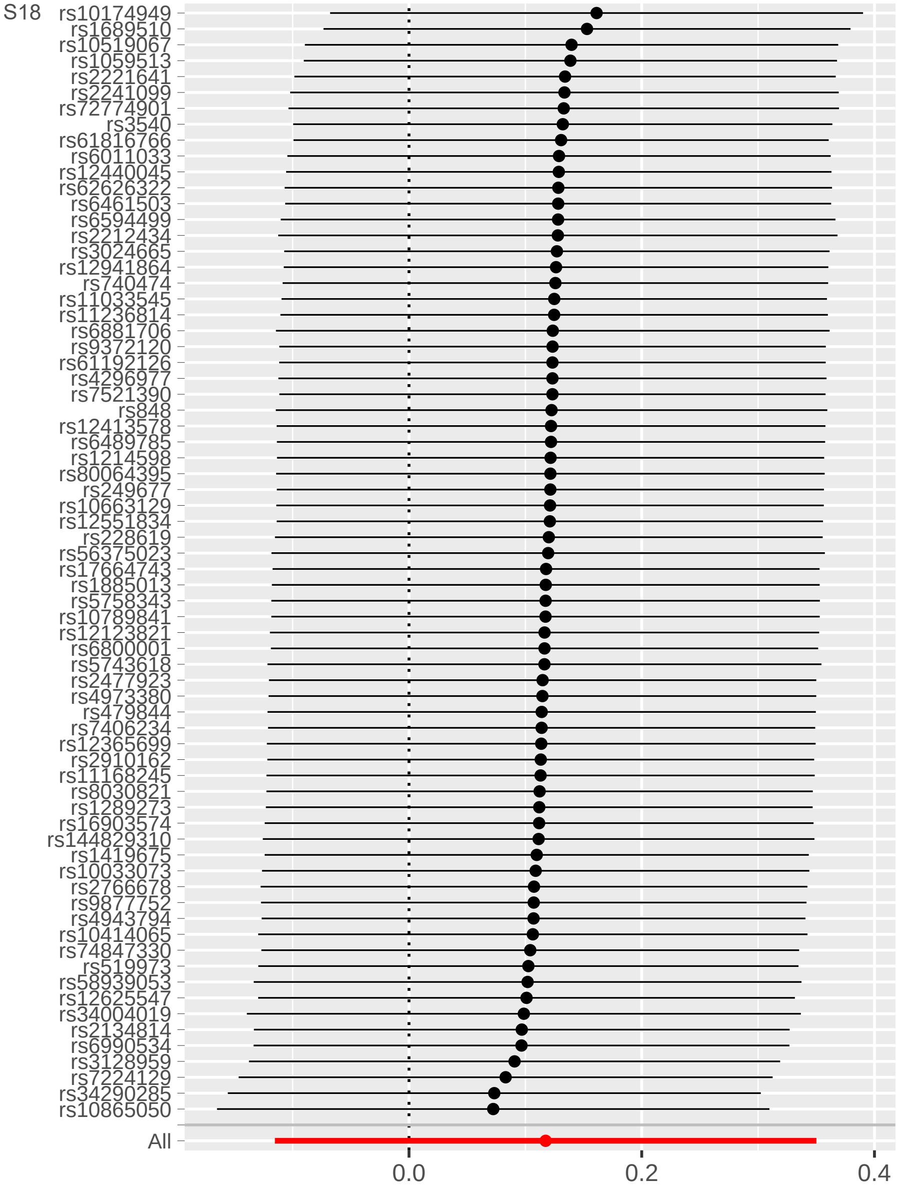
S18
rs10174949
rs1689510
rs10519067
rs1059513
rs2221641
rs2241099
rs72774901
rs3540
rs61816766
rs6011033
rs12440045
rs62626322
rs6461503
rs6594499
rs2212434
rs3024665
rs12941864
rs740474
rs11033545
rs11236814
rs6881706
rs9372120
rs61192126
rs4296977
rs7521390
rs848
rs12413578
rs6489785
rs1214598
rs80064395
rs249677
rs10663129
rs12551834
rs228619
rs56375023
rs17664743
rs1885013
rs5758343
rs10789841
rs12123821
rs6800001
rs5743618
rs2477923
rs4973380
rs479844
rs7406234
rs12365699
rs2910162
rs11168245
rs8030821
rs1289273
rs16903574
rs144829310
rs1419675
rs10033073
rs2766678
rs9877752
rs4943794
rs10414065
rs74847330
rs519973
rs58939053
rs12625547
rs34004019
rs2134814
rs6990534
rs3128959
rs7224129
rs34290285
rs10865050
All
0.0
0.2
0.4

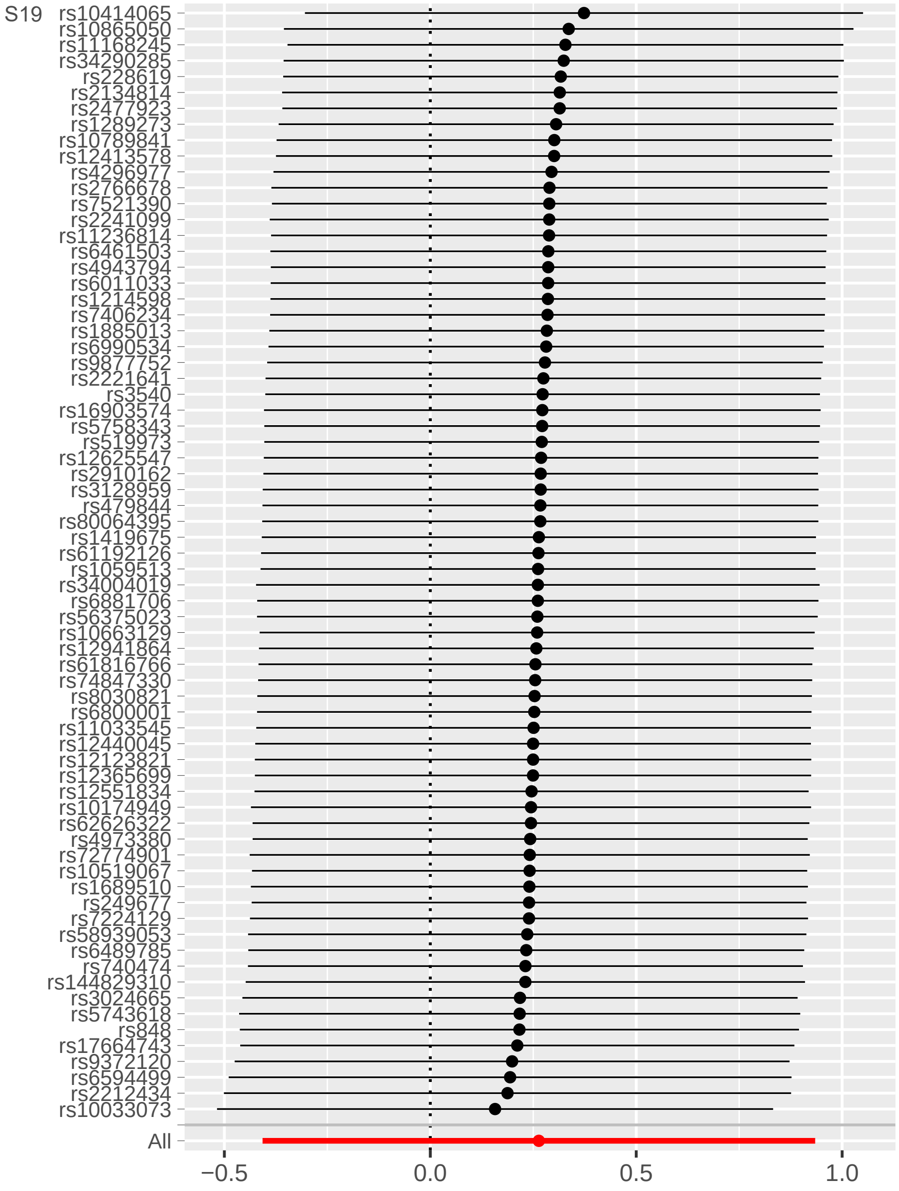
S19
rs10414065
rs10865050
rs11168245
rs34290285
rs228619
rs2134814
rs2477923
rs1289273
rs10789841
rs12413578
rs4296977
rs2766678
rs7521390
rs2241099
rs11236814
rs6461503
rs4943794
rs6011033
rs1214598
rs7406234
rs1885013
rs6990534
rs9877752
rs2221641
rs3540
rs16903574
rs5758343
rs519973
rs12625547
rs2910162
rs3128959
rs479844
rs80064395
rs1419675
rs61192126
rs1059513
rs34004019
rs6881706
rs56375023
rs10663129
rs12941864
rs61816766
rs74847330
rs8030821
rs6800001
rs11033545
rs12440045
rs12123821
rs12365699
rs12551834
rs10174949
rs62626322
rs4973380
rs72774901
rs10519067
rs1689510
rs249677
rs7224129
rs58939053
rs6489785
rs740474
rs144829310
rs3024665
rs5743618
rs848
rs17664743
rs9372120
rs6594499
rs2212434
rs10033073
All
−0.5
0.0
0.5
1.0

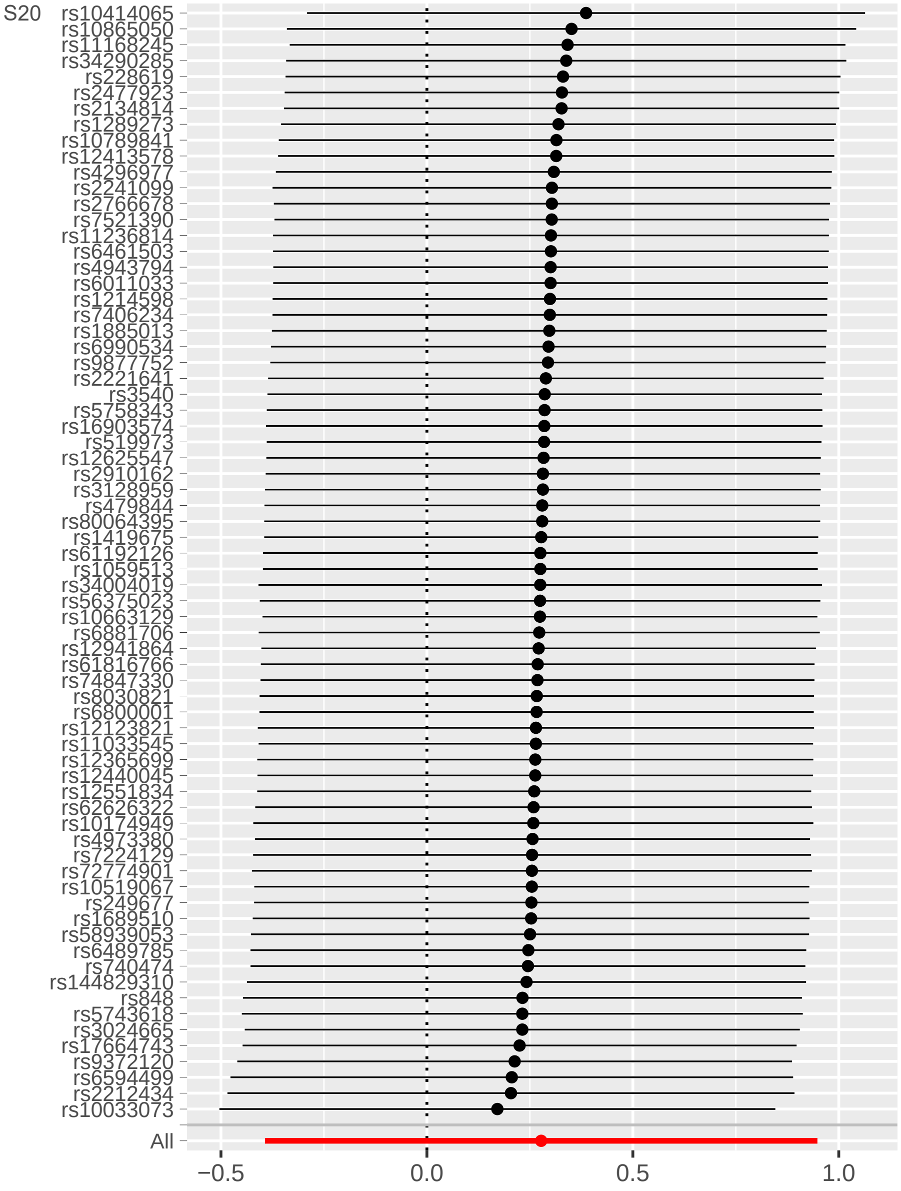

S20
rs10414065
rs10865050
rs11168245
rs34290285
rs228619
rs2477923
rs2134814
rs1289273
rs10789841
rs12413578
rs4296977
rs2241099
rs2766678
rs7521390
rs11236814
rs6461503
rs4943794
rs6011033
rs1214598
rs7406234
rs1885013
rs6990534
rs9877752
rs2221641
rs3540
rs5758343
rs16903574
rs519973
rs12625547
rs2910162
rs3128959
rs479844
rs80064395
rs1419675
rs61192126
rs1059513
rs34004019
rs56375023
rs10663129
rs6881706
rs12941864
rs61816766
rs74847330
rs8030821
rs6800001
rs12123821
rs11033545
rs12365699
rs12440045
rs12551834
rs62626322
rs10174949
rs4973380
rs7224129
rs72774901
rs10519067
rs249677
rs1689510
rs58939053
rs6489785
rs740474
rs144829310
rs848
rs5743618
rs3024665
rs17664743
rs9372120
rs6594499
rs2212434
rs10033073
All
−0.5
0.0
0.5
1.0

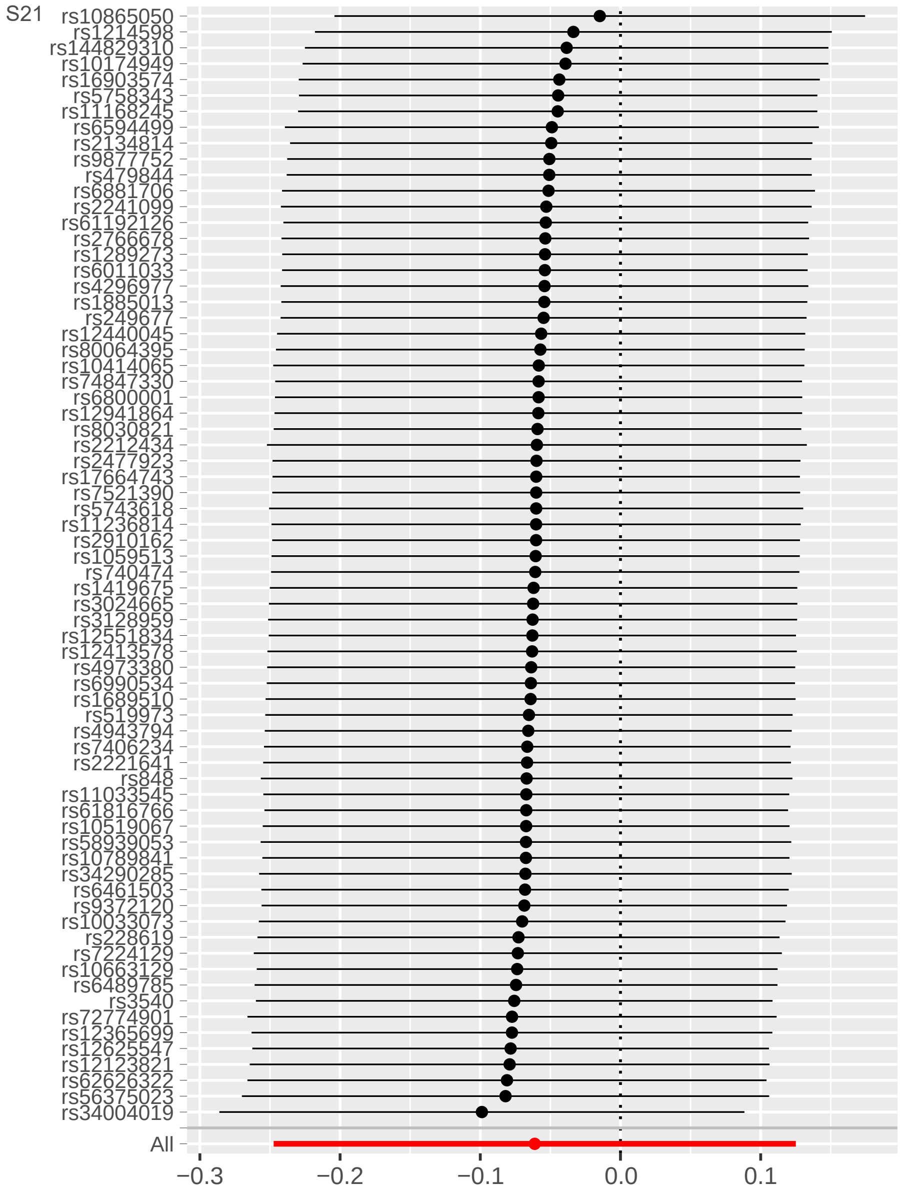
S21
rs10865050
rs1214598
rs144829310
rs10174949
rs16903574
rs5758343
rs11168245
rs6594499
rs2134814
rs9877752
rs479844
rs6881706
rs2241099
rs61192126
rs2766678
rs1289273
rs6011033
rs4296977
rs1885013
rs249677
rs12440045
rs80064395
rs10414065
rs74847330
rs6800001
rs12941864
rs8030821
rs2212434
rs2477923
rs17664743
rs7521390
rs5743618
rs11236814
rs2910162
rs1059513
rs740474
rs1419675
rs3024665
rs3128959
rs12551834
rs12413578
rs4973380
rs6990534
rs1689510
rs519973
rs4943794
rs7406234
rs2221641
rs848
rs11033545
rs61816766
rs10519067
rs58939053
rs10789841
rs34290285
rs6461503
rs9372120
rs10033073
rs228619
rs7224129
rs10663129
rs6489785
rs3540
rs72774901
rs12365699
rs12625547
rs12123821
rs62626322
rs56375023
rs34004019
All
−0.3
−0.2
−0.1
0.0
0.1

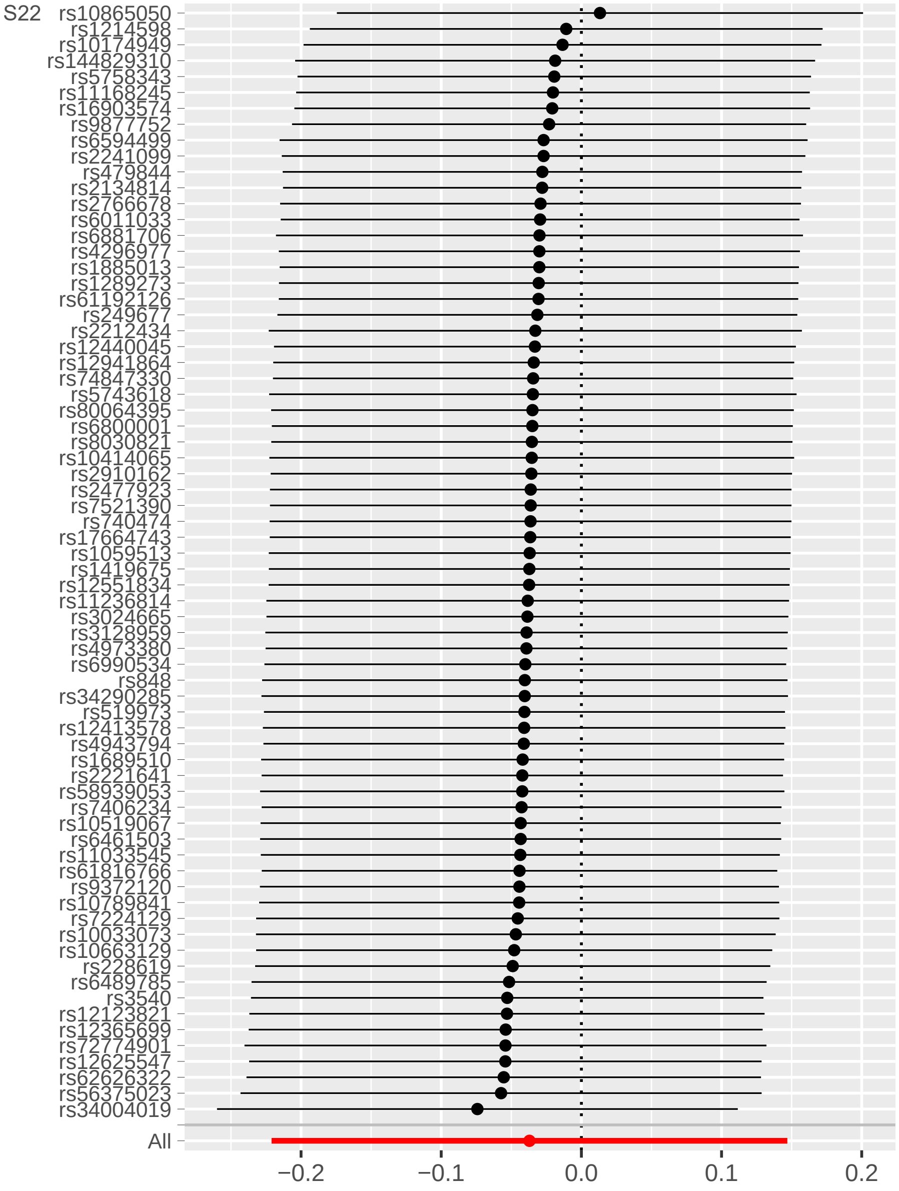
S22
rs10865050
rs1214598
rs10174949
rs144829310
rs5758343
rs11168245
rs16903574
rs9877752
rs6594499
rs2241099
rs479844
rs2134814
rs2766678
rs6011033
rs6881706
rs4296977
rs1885013
rs1289273
rs61192126
rs249677
rs2212434
rs12440045
rs12941864
rs74847330
rs5743618
rs80064395
rs6800001
rs8030821
rs10414065
rs2910162
rs2477923
rs7521390
rs740474
rs17664743
rs1059513
rs1419675
rs12551834
rs11236814
rs3024665
rs3128959
rs4973380
rs6990534
rs848
rs34290285
rs519973
rs12413578
rs4943794
rs1689510
rs2221641
rs58939053
rs7406234
rs10519067
rs6461503
rs11033545
rs61816766
rs9372120
rs10789841
rs7224129
rs10033073
rs10663129
rs228619
rs6489785
rs3540
rs12123821
rs12365699
rs72774901
rs12625547
rs62626322
rs56375023
rs34004019
All
−0.2
−0.1
0.0
0.1
0.2

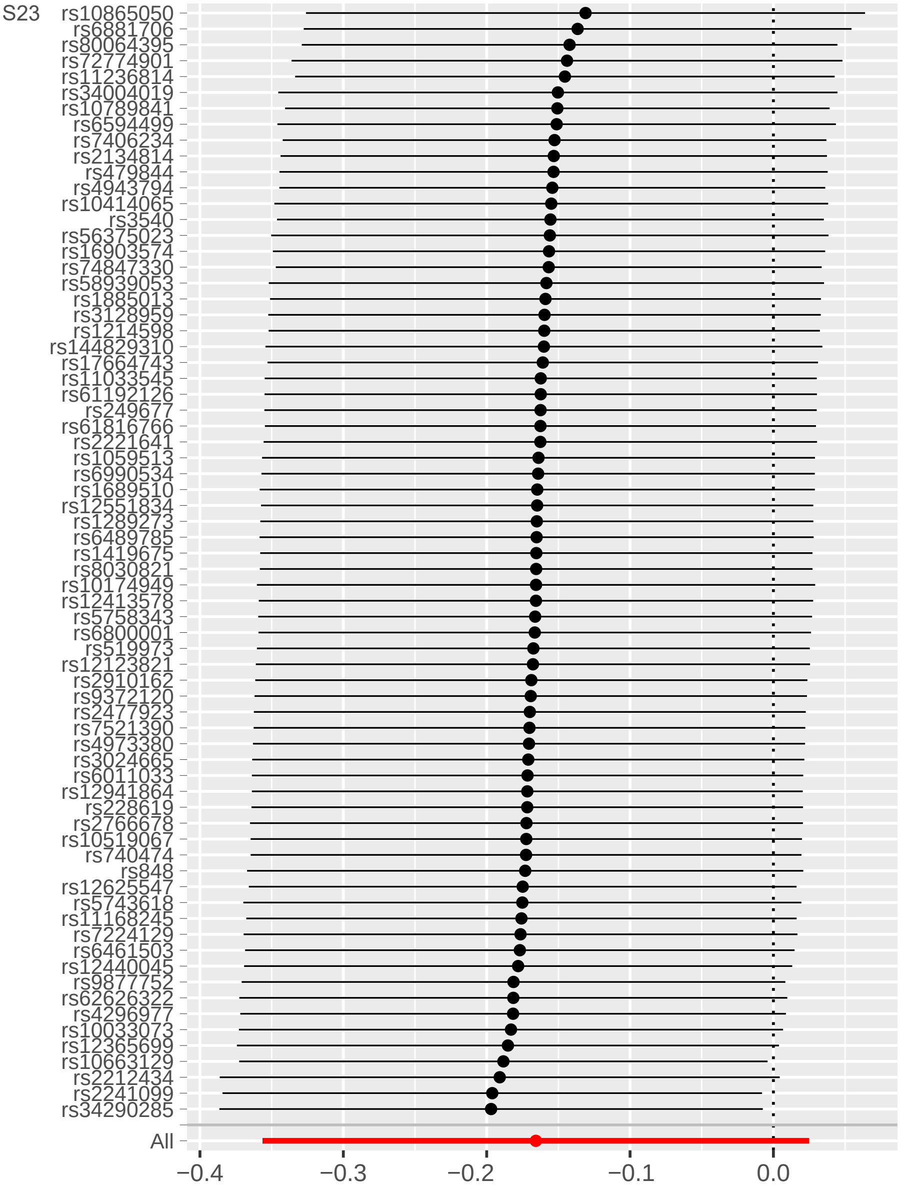

S23
rs10865050
rs6881706
rs80064395
rs72774901
rs11236814
rs34004019
rs10789841
rs6594499
rs7406234
rs2134814
rs479844
rs4943794
rs10414065
rs3540
rs56375023
rs16903574
rs74847330
rs58939053
rs1885013
rs3128959
rs1214598
rs144829310
rs17664743
rs11033545
rs61192126
rs249677
rs61816766
rs2221641
rs1059513
rs6990534
rs1689510
rs12551834
rs1289273
rs6489785
rs1419675
rs8030821
rs10174949
rs12413578
rs5758343
rs6800001
rs519973
rs12123821
rs2910162
rs9372120
rs2477923
rs7521390
rs4973380
rs3024665
rs6011033
rs12941864
rs228619
rs2766678
rs10519067
rs740474
rs848
rs12625547
rs5743618
rs11168245
rs7224129
rs6461503
rs12440045
rs9877752
rs62626322
rs4296977
rs10033073
rs12365699
rs10663129
rs2212434
rs2241099
rs34290285
All
−0.4
−0.3
−0.2
−0.1
0.0

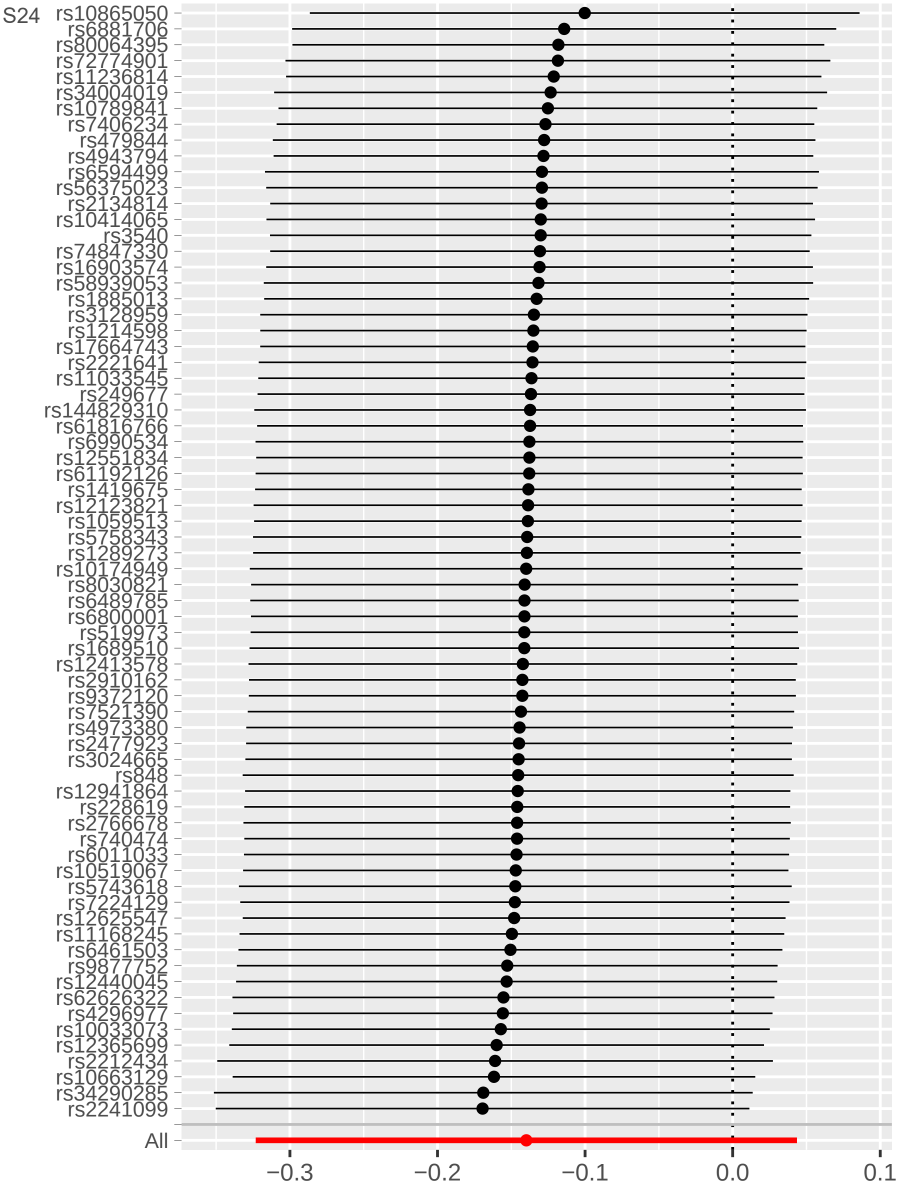

S24
rs10865050
rs6881706
rs80064395
rs72774901
rs11236814
rs34004019
rs10789841
rs7406234
rs479844
rs4943794
rs6594499
rs56375023
rs2134814
rs10414065
rs3540
rs74847330
rs16903574
rs58939053
rs1885013
rs3128959
rs1214598
rs17664743
rs2221641
rs11033545
rs249677
rs144829310
rs61816766
rs6990534
rs12551834
rs61192126
rs1419675
rs12123821
rs1059513
rs5758343
rs1289273
rs10174949
rs8030821
rs6489785
rs6800001
rs519973
rs1689510
rs12413578
rs2910162
rs9372120
rs7521390
rs4973380
rs2477923
rs3024665
rs848
rs12941864
rs228619
rs2766678
rs740474
rs6011033
rs10519067
rs5743618
rs7224129
rs12625547
rs11168245
rs6461503
rs9877752
rs12440045
rs62626322
rs4296977
rs10033073
rs12365699
rs2212434
rs10663129
rs34290285
rs2241099
All
−0.3
−0.2
−0.1
0.0
0.1

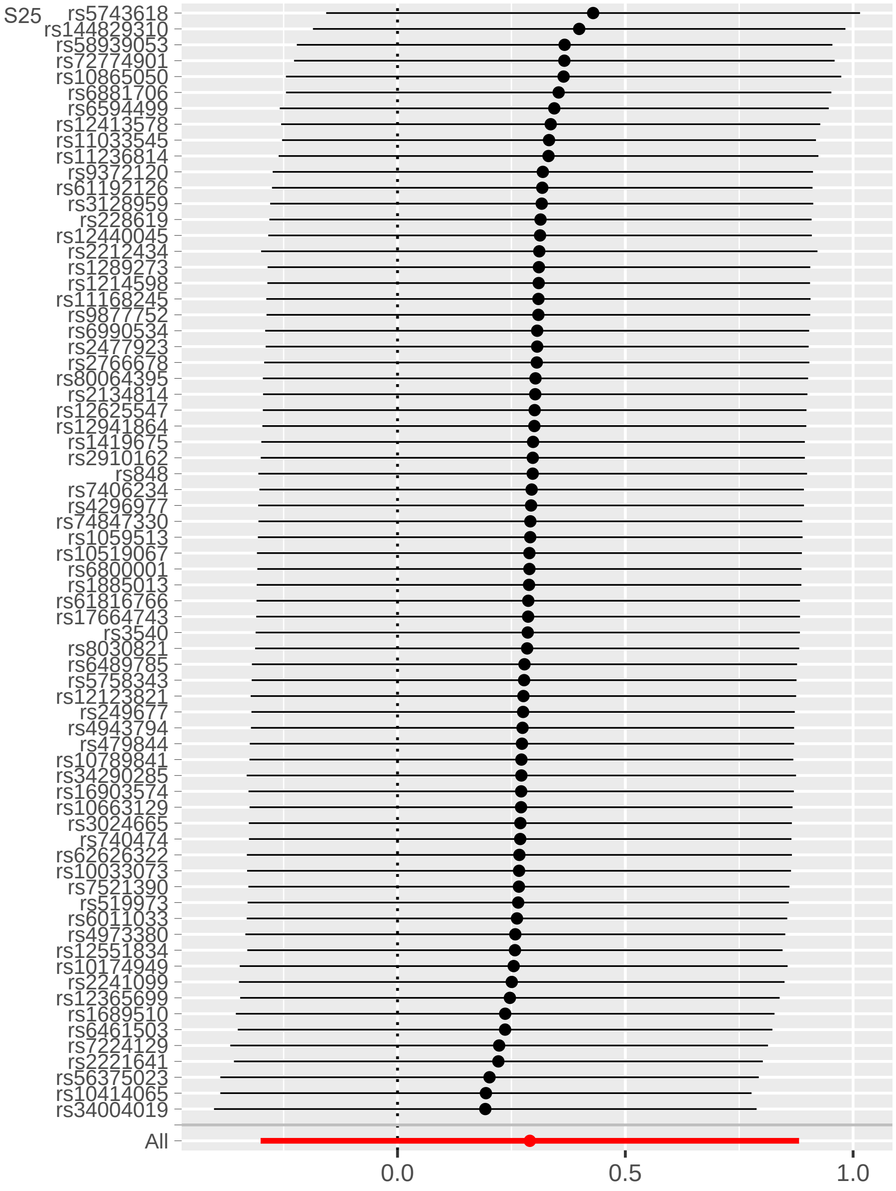
S25
rs5743618
rs144829310
rs58939053
rs72774901
rs10865050
rs6881706
rs6594499
rs12413578
rs11033545
rs11236814
rs9372120
rs61192126
rs3128959
rs228619
rs12440045
rs2212434
rs1289273
rs1214598
rs11168245
rs9877752
rs6990534
rs2477923
rs2766678
rs80064395
rs2134814
rs12625547
rs12941864
rs1419675
rs2910162
rs848
rs7406234
rs4296977
rs74847330
rs1059513
rs10519067
rs6800001
rs1885013
rs61816766
rs17664743
rs3540
rs8030821
rs6489785
rs5758343
rs12123821
rs249677
rs4943794
rs479844
rs10789841
rs34290285
rs16903574
rs10663129
rs3024665
rs740474
rs62626322
rs10033073
rs7521390
rs519973
rs6011033
rs4973380
rs12551834
rs10174949
rs2241099
rs12365699
rs1689510
rs6461503
rs7224129
rs2221641
rs56375023
rs10414065
rs34004019
All
0.0
0.5
1.0

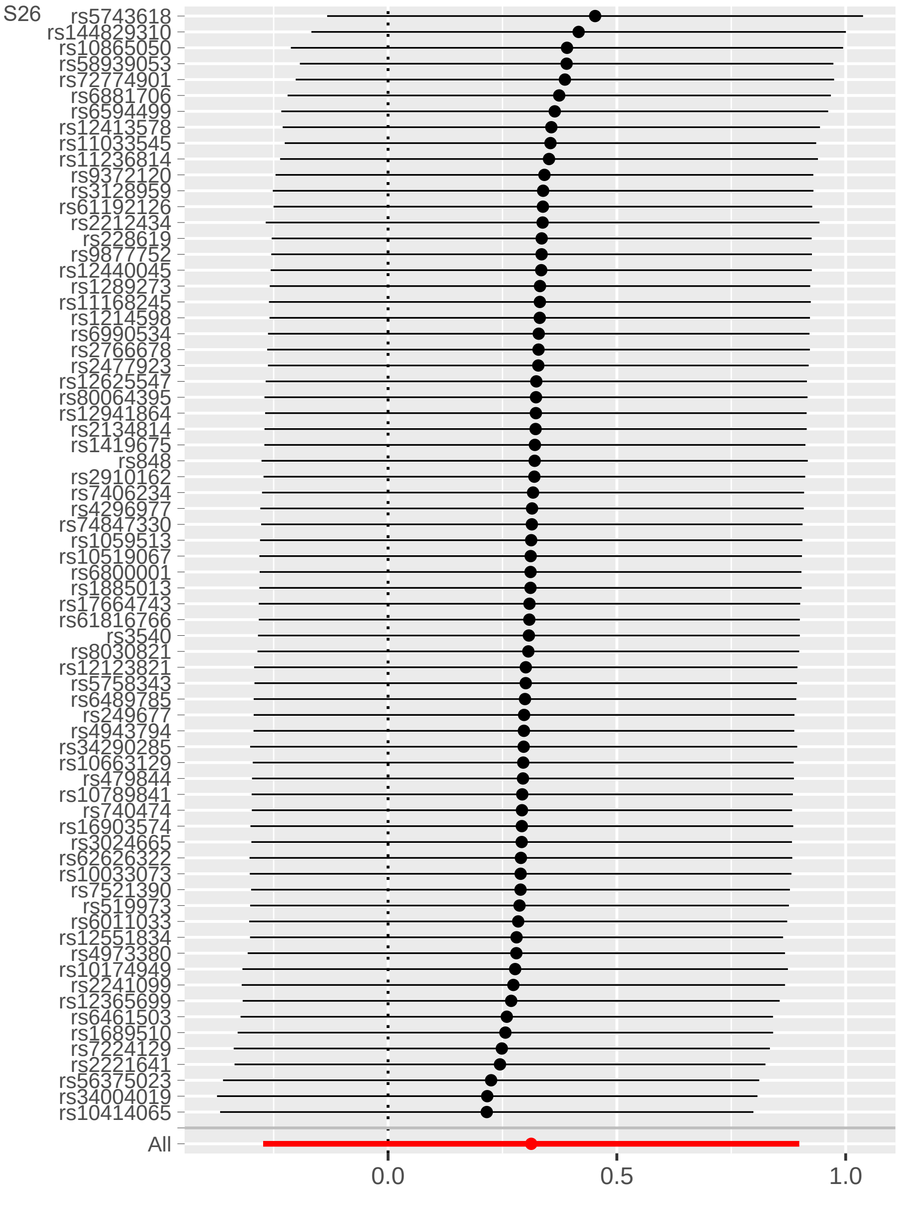

S26
rs5743618
rs144829310
rs10865050
rs58939053
rs72774901
rs6881706
rs6594499
rs12413578
rs11033545
rs11236814
rs9372120
rs3128959
rs61192126
rs2212434
rs228619
rs9877752
rs12440045
rs1289273
rs11168245
rs1214598
rs6990534
rs2766678
rs2477923
rs12625547
rs80064395
rs12941864
rs2134814
rs1419675
rs848
rs2910162
rs7406234
rs4296977
rs74847330
rs1059513
rs10519067
rs6800001
rs1885013
rs17664743
rs61816766
rs3540
rs8030821
rs12123821
rs5758343
rs6489785
rs249677
rs4943794
rs34290285
rs10663129
rs479844
rs10789841
rs740474
rs16903574
rs3024665
rs62626322
rs10033073
rs7521390
rs519973
rs6011033
rs12551834
rs4973380
rs10174949
rs2241099
rs12365699
rs6461503
rs1689510
rs7224129
rs2221641
rs56375023
rs34004019
rs10414065
All
0.0
0.5
1.0

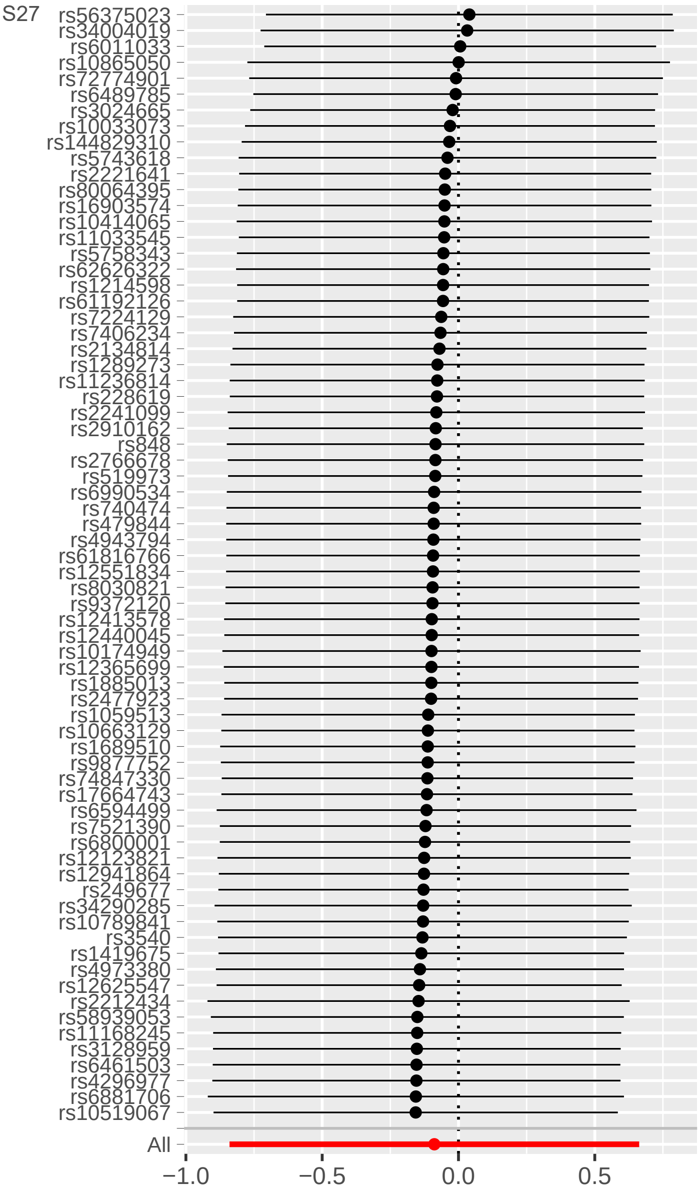
S27
rs56375023
rs34004019
rs6011033
rs10865050
rs72774901
rs6489785
rs3024665
rs10033073
rs144829310
rs5743618
rs2221641
rs80064395
rs16903574
rs10414065
rs11033545
rs5758343
rs62626322
rs1214598
rs61192126
rs7224129
rs7406234
rs2134814
rs1289273
rs11236814
rs228619
rs2241099
rs2910162
rs848
rs2766678
rs519973
rs6990534
rs740474
rs479844
rs4943794
rs61816766
rs12551834
rs8030821
rs9372120
rs12413578
rs12440045
rs10174949
rs12365699
rs1885013
rs2477923
rs1059513
rs10663129
rs1689510
rs9877752
rs74847330
rs17664743
rs6594499
rs7521390
rs6800001
rs12123821
rs12941864
rs249677
rs34290285
rs10789841
rs3540
rs1419675
rs4973380
rs12625547
rs2212434
rs58939053
rs11168245
rs3128959
rs6461503
rs4296977
rs6881706
rs10519067
All
−1.0
−0.5
0.0
0.5

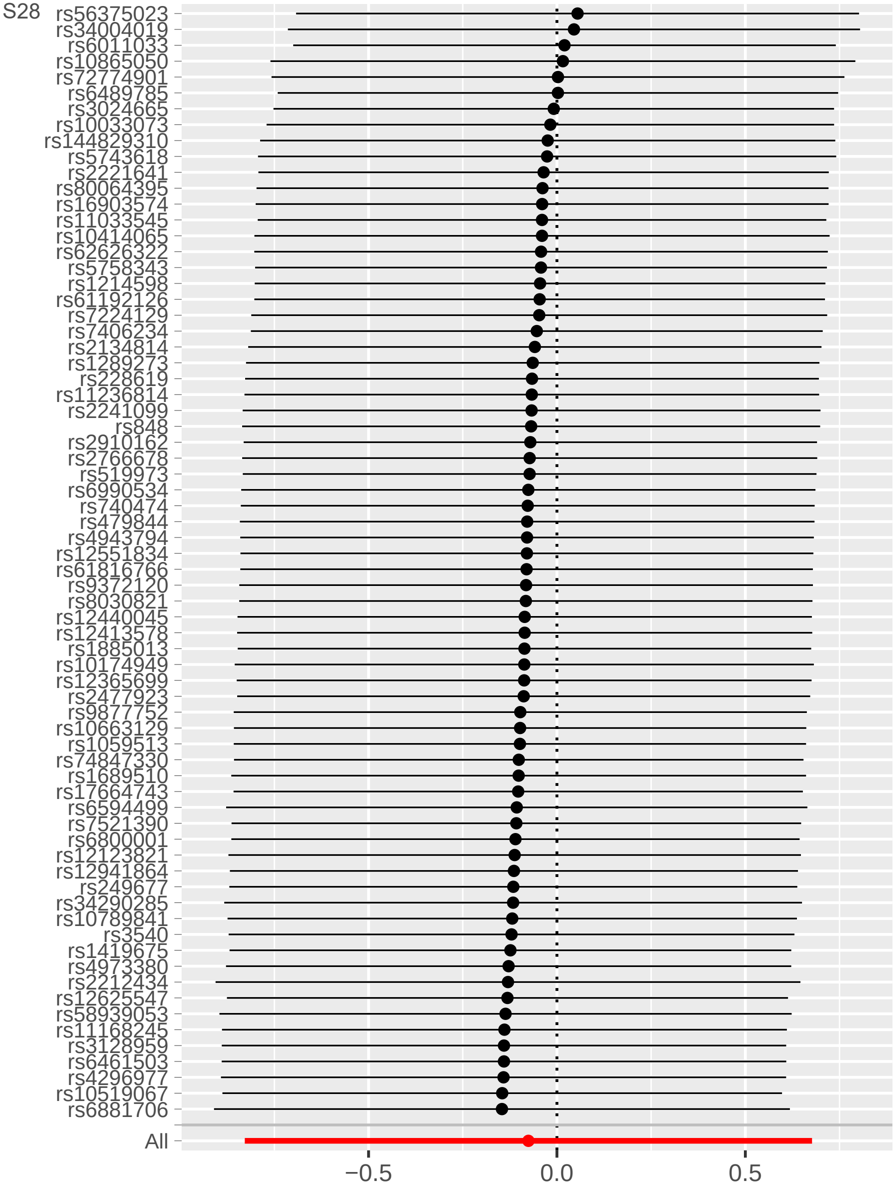

S28
rs56375023
rs34004019
rs6011033
rs10865050
rs72774901
rs6489785
rs3024665
rs10033073
rs144829310
rs5743618
rs2221641
rs80064395
rs16903574
rs11033545
rs10414065
rs62626322
rs5758343
rs1214598
rs61192126
rs7224129
rs7406234
rs2134814
rs1289273
rs228619
rs11236814
rs2241099
rs848
rs2910162
rs2766678
rs519973
rs6990534
rs740474
rs479844
rs4943794
rs12551834
rs61816766
rs9372120
rs8030821
rs12440045
rs12413578
rs1885013
rs10174949
rs12365699
rs2477923
rs9877752
rs10663129
rs1059513
rs74847330
rs1689510
rs17664743
rs6594499
rs7521390
rs6800001
rs12123821
rs12941864
rs249677
rs34290285
rs10789841
rs3540
rs1419675
rs4973380
rs2212434
rs12625547
rs58939053
rs11168245
rs3128959
rs6461503
rs4296977
rs10519067
rs6881706
All
−0.5
0.0
0.5

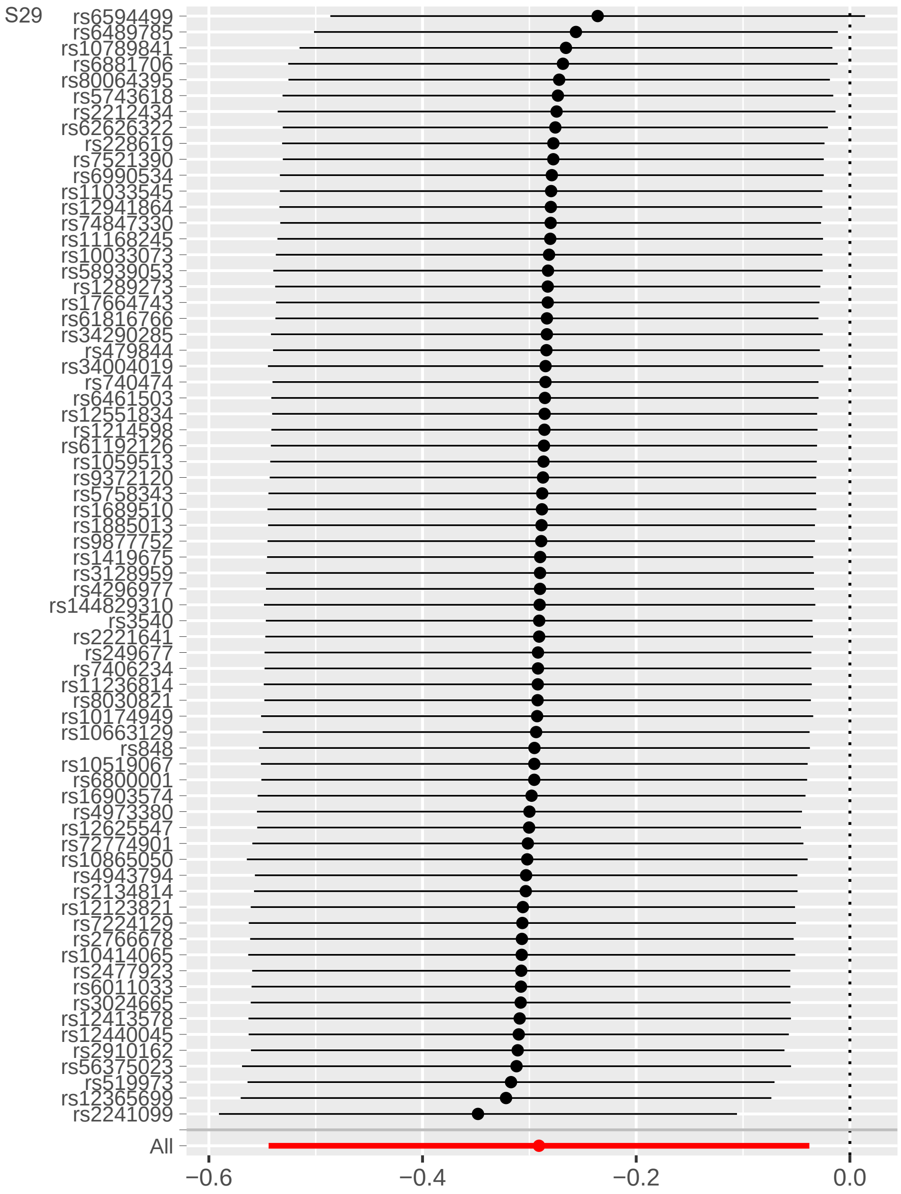

S29
rs6594499
rs6489785
rs10789841
rs6881706
rs80064395
rs5743618
rs2212434
rs62626322
rs228619
rs7521390
rs6990534
rs11033545
rs12941864
rs74847330
rs11168245
rs10033073
rs58939053
rs1289273
rs17664743
rs61816766
rs34290285
rs479844
rs34004019
rs740474
rs6461503
rs12551834
rs1214598
rs61192126
rs1059513
rs9372120
rs5758343
rs1689510
rs1885013
rs9877752
rs1419675
rs3128959
rs4296977
rs144829310
rs3540
rs2221641
rs249677
rs7406234
rs11236814
rs8030821
rs10174949
rs10663129
rs848
rs10519067
rs6800001
rs16903574
rs4973380
rs12625547
rs72774901
rs10865050
rs4943794
rs2134814
rs12123821
rs7224129
rs2766678
rs10414065
rs2477923
rs6011033
rs3024665
rs12413578
rs12440045
rs2910162
rs56375023
rs519973
rs12365699
rs2241099
All
−0.6
−0.4
−0.2
0.0

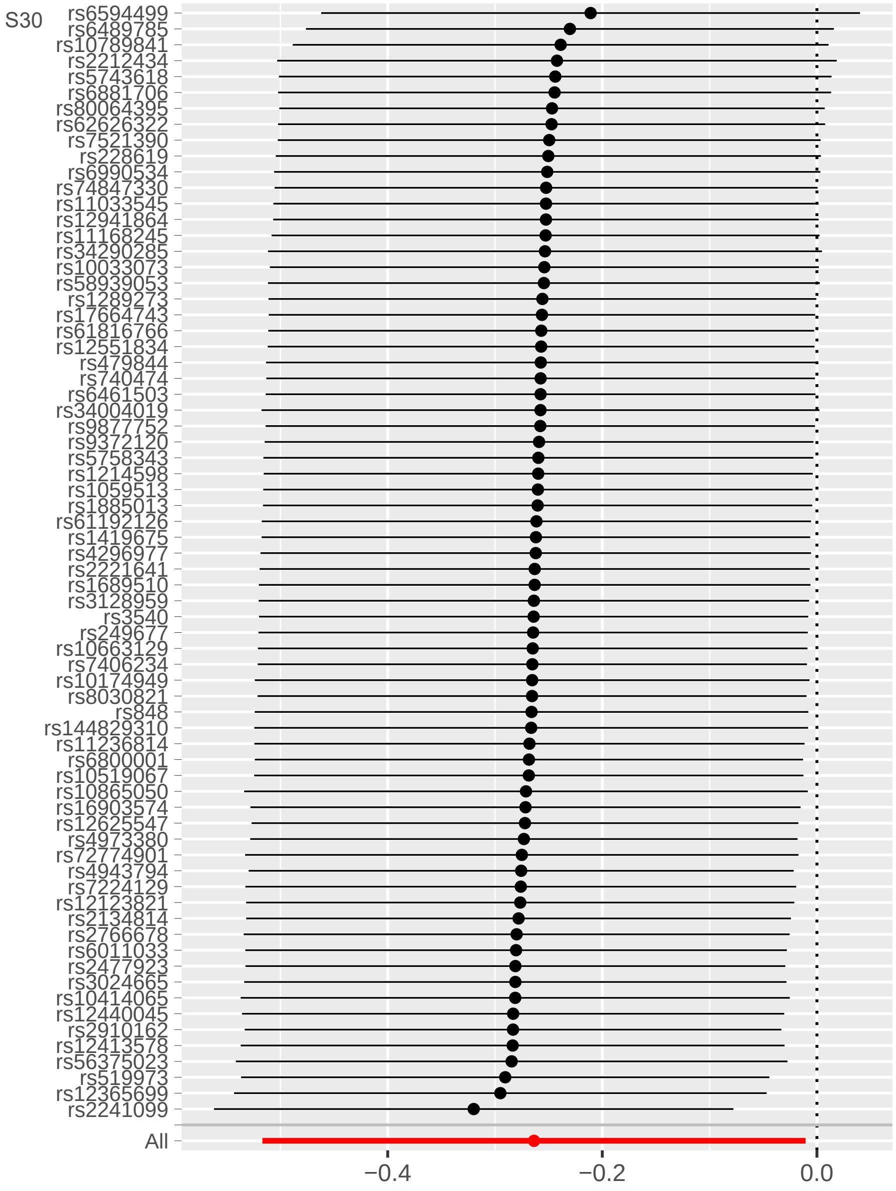
S30
rs6594499
rs6489785
rs10789841
rs2212434
rs5743618
rs6881706
rs80064395
rs62626322
rs7521390
rs228619
rs6990534
rs74847330
rs11033545
rs12941864
rs11168245
rs34290285
rs10033073
rs58939053
rs1289273
rs17664743
rs61816766
rs12551834
rs479844
rs740474
rs6461503
rs34004019
rs9877752
rs9372120
rs5758343
rs1214598
rs1059513
rs1885013
rs61192126
rs1419675
rs4296977
rs2221641
rs1689510
rs3128959
rs3540
rs249677
rs10663129
rs7406234
rs10174949
rs8030821
rs848
rs144829310
rs11236814
rs6800001
rs10519067
rs10865050
rs16903574
rs12625547
rs4973380
rs72774901
rs4943794
rs7224129
rs12123821
rs2134814
rs2766678
rs6011033
rs2477923
rs3024665
rs10414065
rs12440045
rs2910162
rs12413578
rs56375023
rs519973
rs12365699
rs2241099
All
−0.4
−0.2
0.0

S31

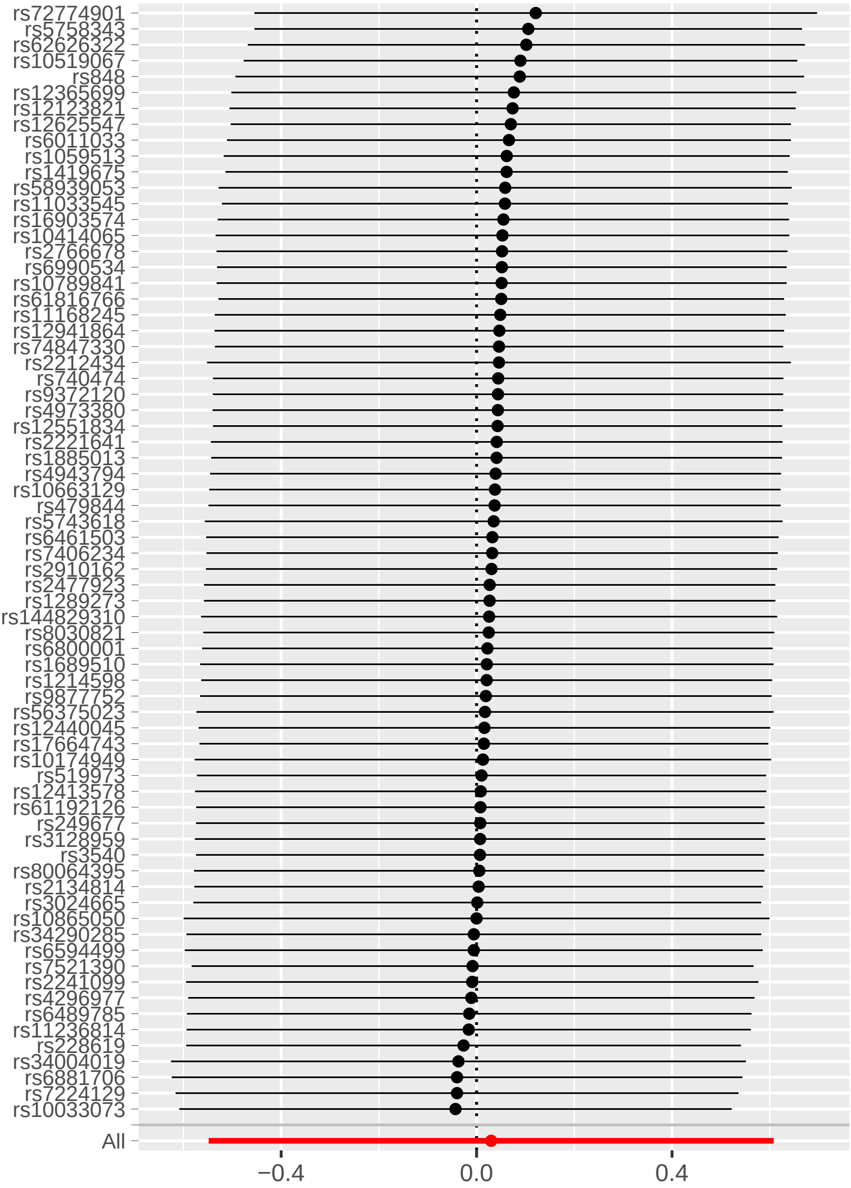
rs72774901
rs5758343
rs62626322
rs10519067
rs848
rs12365699
rs12123821
rs12625547
rs6011033
rs1059513
rs1419675
rs58939053
rs11033545
rs16903574
rs10414065
rs2766678
rs6990534
rs10789841
rs61816766
rs11168245
rs12941864
rs74847330
rs2212434
rs740474
rs9372120
rs4973380
rs12551834
rs2221641
rs1885013
rs4943794
rs10663129
rs479844
rs5743618
rs6461503
rs7406234
rs2910162
rs2477923
rs1289273
rs144829310
rs8030821
rs6800001
rs1689510
rs1214598
rs9877752
rs56375023
rs12440045
rs17664743
rs10174949
rs519973
rs12413578
rs61192126
rs249677
rs3128959
rs3540
rs80064395
rs2134814
rs3024665
rs10865050
rs34290285
rs6594499
rs7521390
rs2241099
rs4296977
rs6489785
rs11236814
rs228619
rs34004019
rs6881706
rs7224129
rs10033073
All
−0.4
0.0
0.4

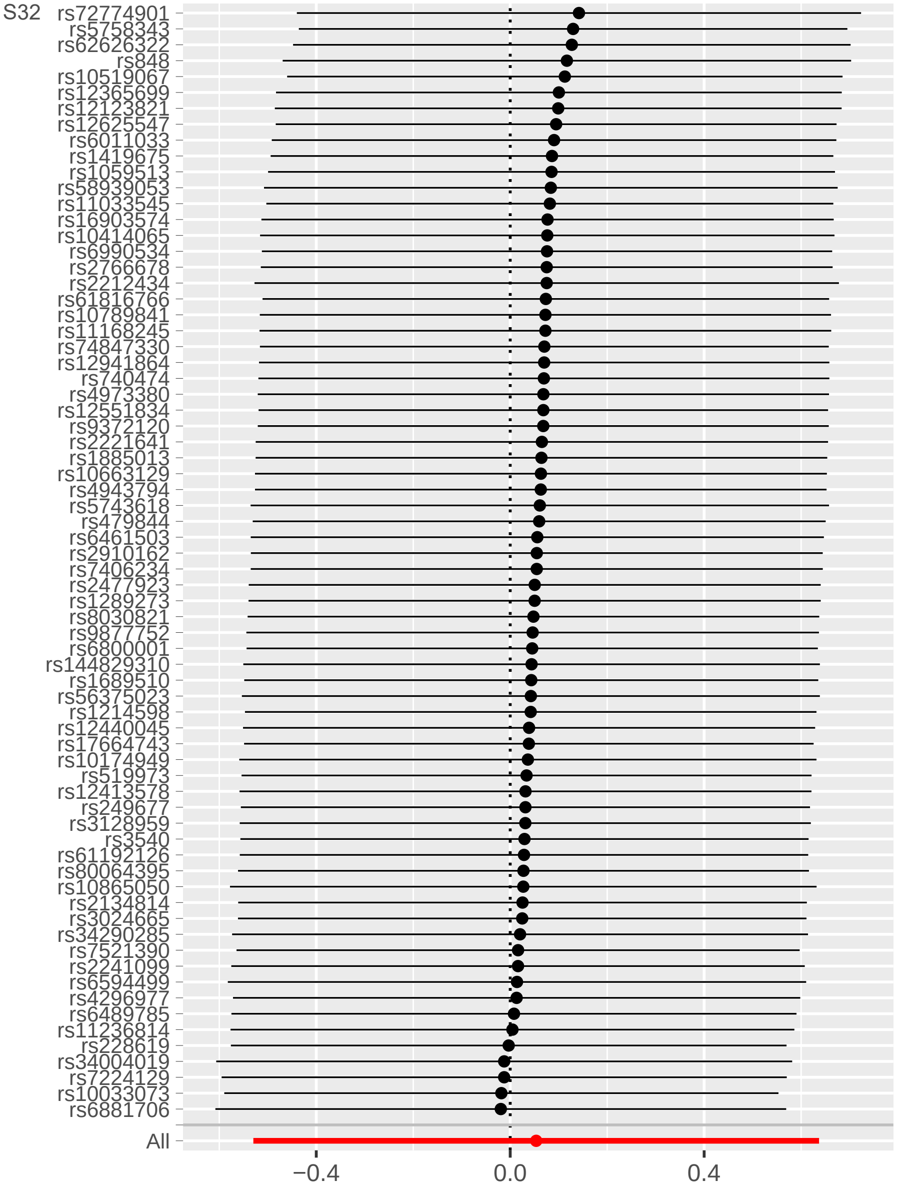
S32
rs72774901
rs5758343
rs62626322
rs848
rs10519067
rs12365699
rs12123821
rs12625547
rs6011033
rs1419675
rs1059513
rs58939053
rs11033545
rs16903574
rs10414065
rs6990534
rs2766678
rs2212434
rs61816766
rs10789841
rs11168245
rs74847330
rs12941864
rs740474
rs4973380
rs12551834
rs9372120
rs2221641
rs1885013
rs10663129
rs4943794
rs5743618
rs479844
rs6461503
rs2910162
rs7406234
rs2477923
rs1289273
rs8030821
rs9877752
rs6800001
rs144829310
rs1689510
rs56375023
rs1214598
rs12440045
rs17664743
rs10174949
rs519973
rs12413578
rs249677
rs3128959
rs3540
rs61192126
rs80064395
rs10865050
rs2134814
rs3024665
rs34290285
rs7521390
rs2241099
rs6594499
rs4296977
rs6489785
rs11236814
rs228619
rs34004019
rs7224129
rs10033073
rs6881706
All
−0.4
0.0
0.4

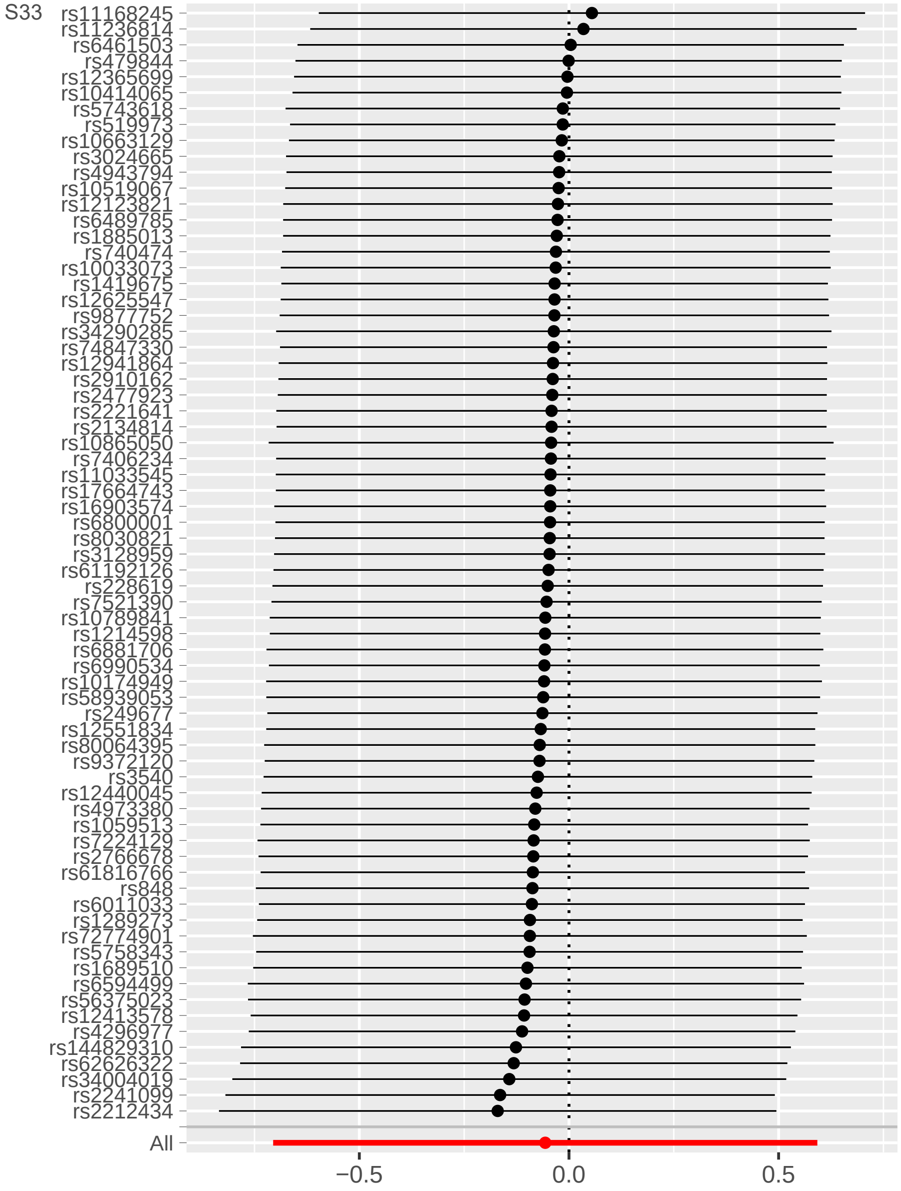
S33
rs11168245
rs11236814
rs6461503
rs479844
rs12365699
rs10414065
rs5743618
rs519973
rs10663129
rs3024665
rs4943794
rs10519067
rs12123821
rs6489785
rs1885013
rs740474
rs10033073
rs1419675
rs12625547
rs9877752
rs34290285
rs74847330
rs12941864
rs2910162
rs2477923
rs2221641
rs2134814
rs10865050
rs7406234
rs11033545
rs17664743
rs16903574
rs6800001
rs8030821
rs3128959
rs61192126
rs228619
rs7521390
rs10789841
rs1214598
rs6881706
rs6990534
rs10174949
rs58939053
rs249677
rs12551834
rs80064395
rs9372120
rs3540
rs12440045
rs4973380
rs1059513
rs7224129
rs2766678
rs61816766
rs848
rs6011033
rs1289273
rs72774901
rs5758343
rs1689510
rs6594499
rs56375023
rs12413578
rs4296977
rs144829310
rs62626322
rs34004019
rs2241099
rs2212434
All
−0.5
0.0
0.5

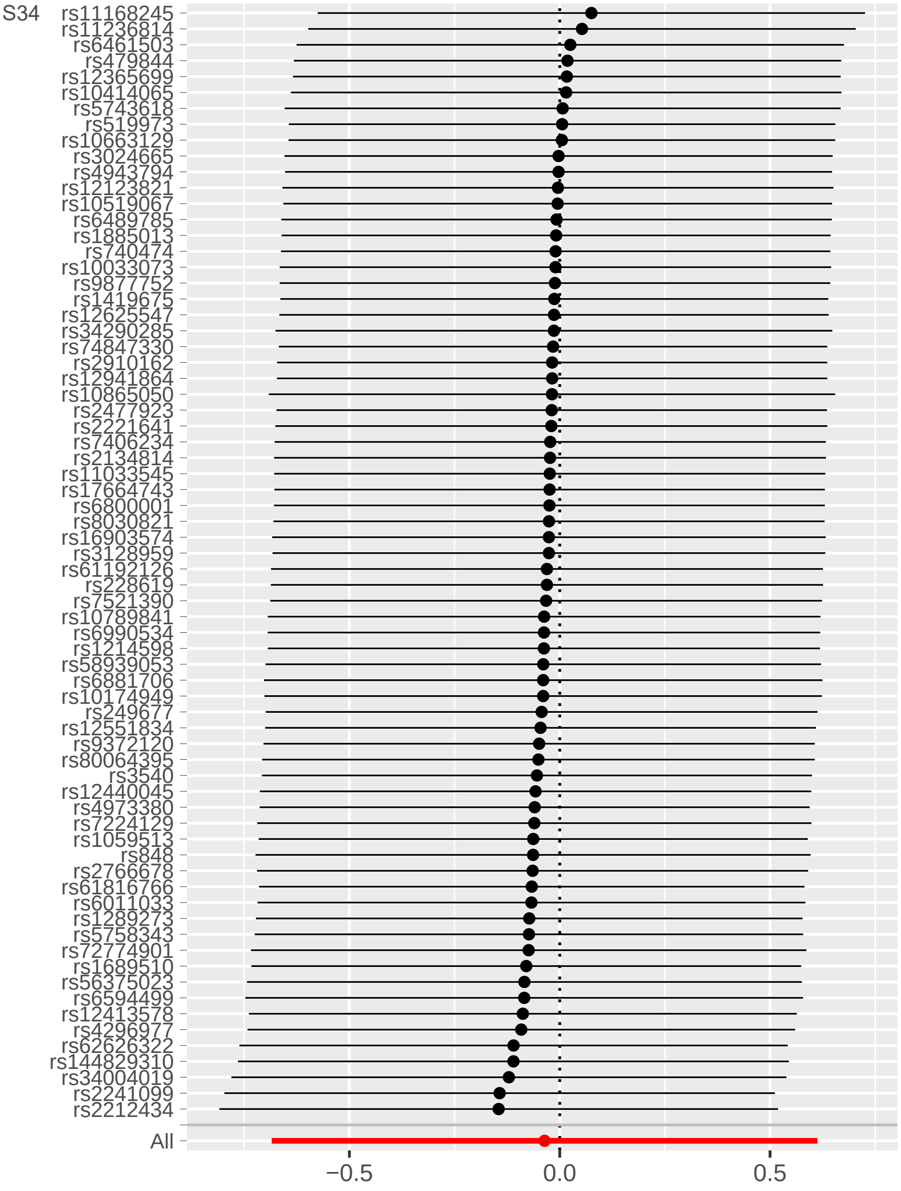

S34
rs11168245
rs11236814
rs6461503
rs479844
rs12365699
rs10414065
rs5743618
rs519973
rs10663129
rs3024665
rs4943794
rs12123821
rs10519067
rs6489785
rs1885013
rs740474
rs10033073
rs9877752
rs1419675
rs12625547
rs34290285
rs74847330
rs2910162
rs12941864
rs10865050
rs2477923
rs2221641
rs7406234
rs2134814
rs11033545
rs17664743
rs6800001
rs8030821
rs16903574
rs3128959
rs61192126
rs228619
rs7521390
rs10789841
rs6990534
rs1214598
rs58939053
rs6881706
rs10174949
rs249677
rs12551834
rs9372120
rs80064395
rs3540
rs12440045
rs4973380
rs7224129
rs1059513
rs848
rs2766678
rs61816766
rs6011033
rs1289273
rs5758343
rs72774901
rs1689510
rs56375023
rs6594499
rs12413578
rs4296977
rs62626322
rs144829310
rs34004019
rs2241099
rs2212434
All
−0.5
0.0
0.5

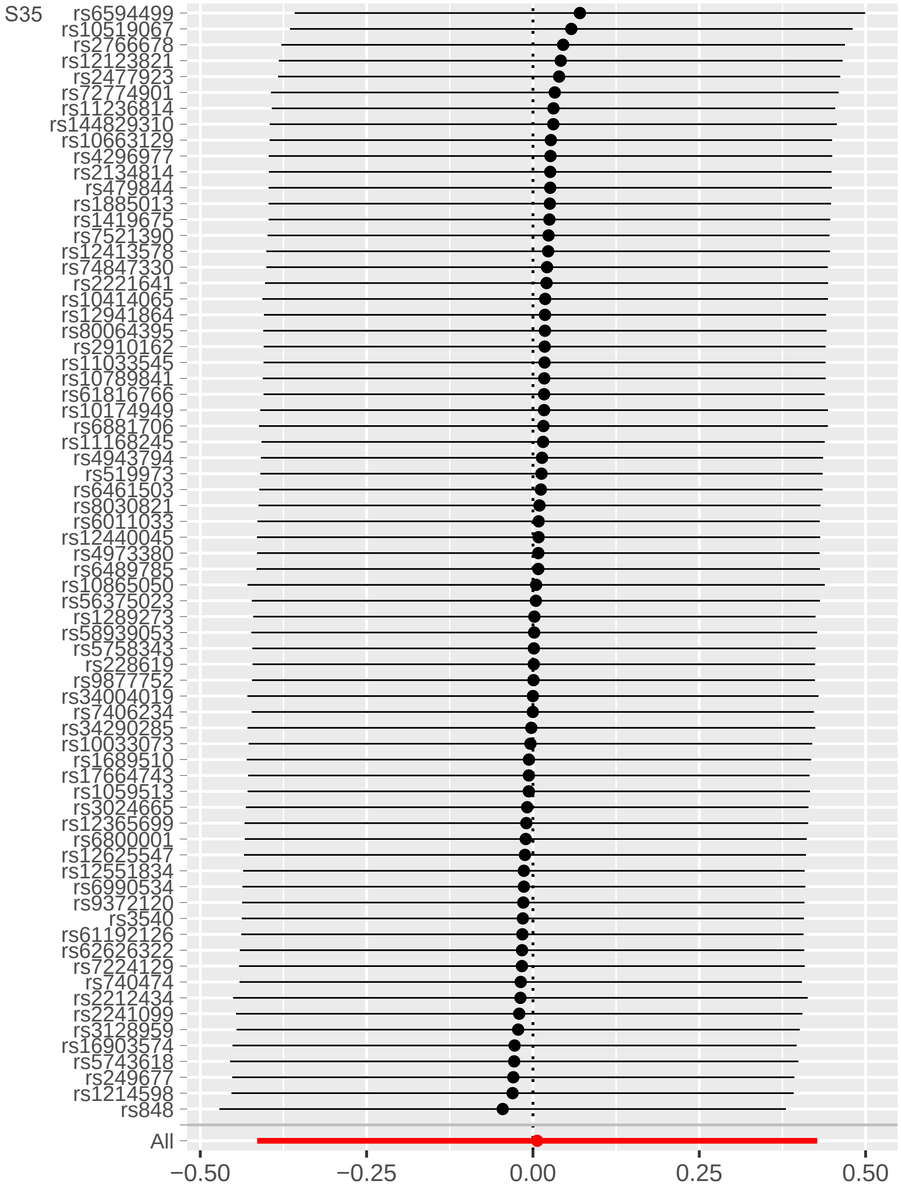

S35
rs6594499
rs10519067
rs2766678
rs12123821
rs2477923
rs72774901
rs11236814
rs144829310
rs10663129
rs4296977
rs2134814
rs479844
rs1885013
rs1419675
rs7521390
rs12413578
rs74847330
rs2221641
rs10414065
rs12941864
rs80064395
rs2910162
rs11033545
rs10789841
rs61816766
rs10174949
rs6881706
rs11168245
rs4943794
rs519973
rs6461503
rs8030821
rs6011033
rs12440045
rs4973380
rs6489785
rs10865050
rs56375023
rs1289273
rs58939053
rs5758343
rs228619
rs9877752
rs34004019
rs7406234
rs34290285
rs10033073
rs1689510
rs17664743
rs1059513
rs3024665
rs12365699
rs6800001
rs12625547
rs12551834
rs6990534
rs9372120
rs3540
rs61192126
rs62626322
rs7224129
rs740474
rs2212434
rs2241099
rs3128959
rs16903574
rs5743618
rs249677
rs1214598
rs848
All
−0.50
−0.25
0.00
0.25
0.50

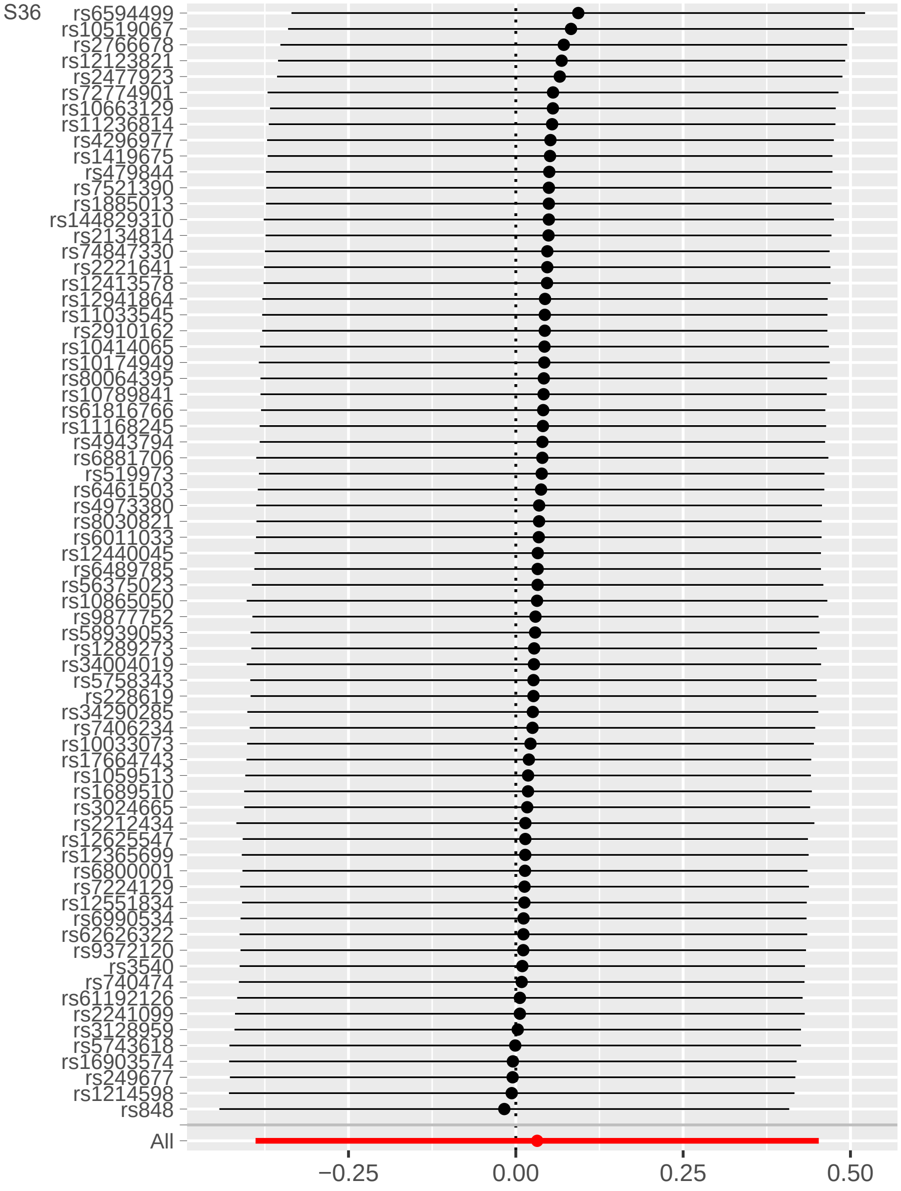
S36
rs6594499
rs10519067
rs2766678
rs12123821
rs2477923
rs72774901
rs10663129
rs11236814
rs4296977
rs1419675
rs479844
rs7521390
rs1885013
rs144829310
rs2134814
rs74847330
rs2221641
rs12413578
rs12941864
rs11033545
rs2910162
rs10414065
rs10174949
rs80064395
rs10789841
rs61816766
rs11168245
rs4943794
rs6881706
rs519973
rs6461503
rs4973380
rs8030821
rs6011033
rs12440045
rs6489785
rs56375023
rs10865050
rs9877752
rs58939053
rs1289273
rs34004019
rs5758343
rs228619
rs34290285
rs7406234
rs10033073
rs17664743
rs1059513
rs1689510
rs3024665
rs2212434
rs12625547
rs12365699
rs6800001
rs7224129
rs12551834
rs6990534
rs62626322
rs9372120
rs3540
rs740474
rs61192126
rs2241099
rs3128959
rs5743618
rs16903574
rs249677
rs1214598
rs848
All
−0.25
0.00
0.25
0.50

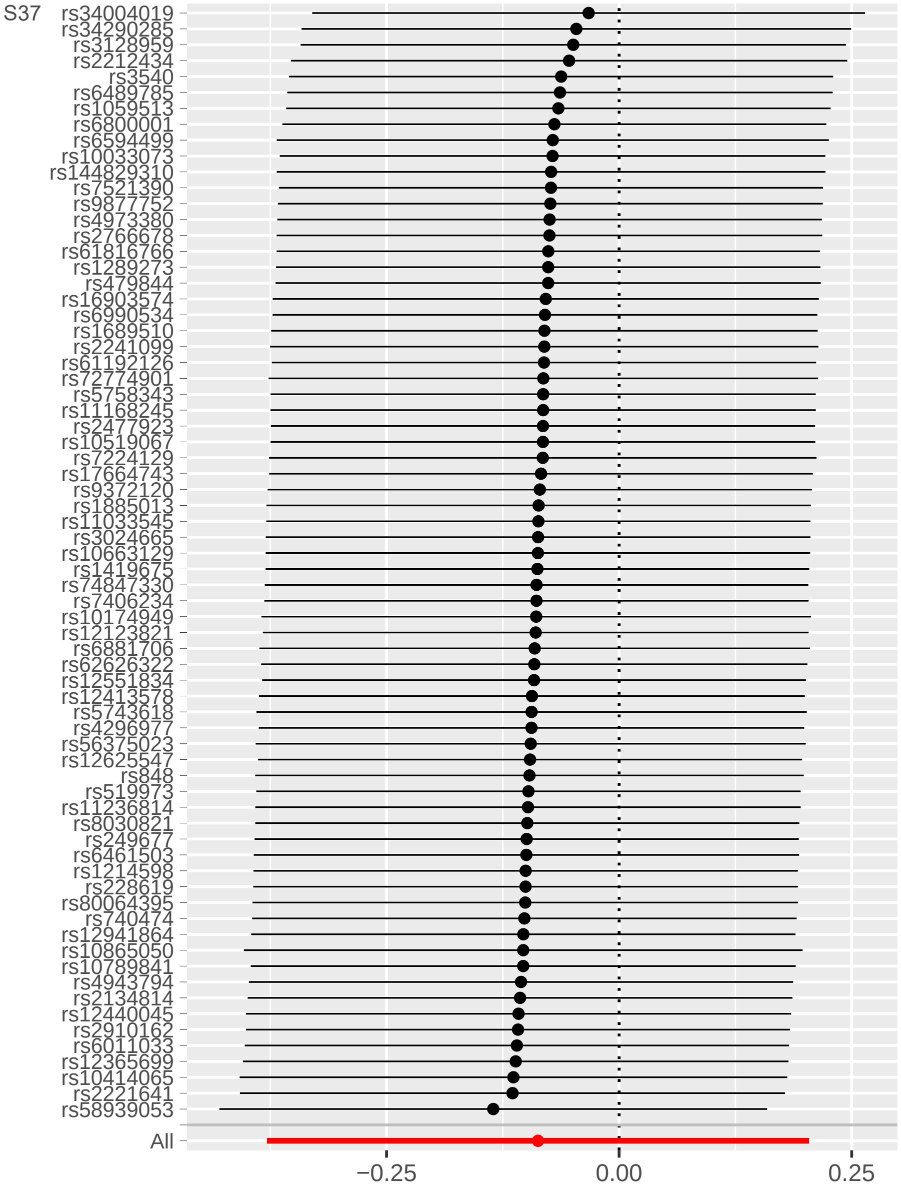
S37
rs34004019
rs34290285
rs3128959
rs2212434
rs3540
rs6489785
rs1059513
rs6800001
rs6594499
rs10033073
rs144829310
rs7521390
rs9877752
rs4973380
rs2766678
rs61816766
rs1289273
rs479844
rs16903574
rs6990534
rs1689510
rs2241099
rs61192126
rs72774901
rs5758343
rs11168245
rs2477923
rs10519067
rs7224129
rs17664743
rs9372120
rs1885013
rs11033545
rs3024665
rs10663129
rs1419675
rs74847330
rs7406234
rs10174949
rs12123821
rs6881706
rs62626322
rs12551834
rs12413578
rs5743618
rs4296977
rs56375023
rs12625547
rs848
rs519973
rs11236814
rs8030821
rs249677
rs6461503
rs1214598
rs228619
rs80064395
rs740474
rs12941864
rs10865050
rs10789841
rs4943794
rs2134814
rs12440045
rs2910162
rs6011033
rs12365699
rs10414065
rs2221641
rs58939053
All
−0.25
0.00
0.25

S38

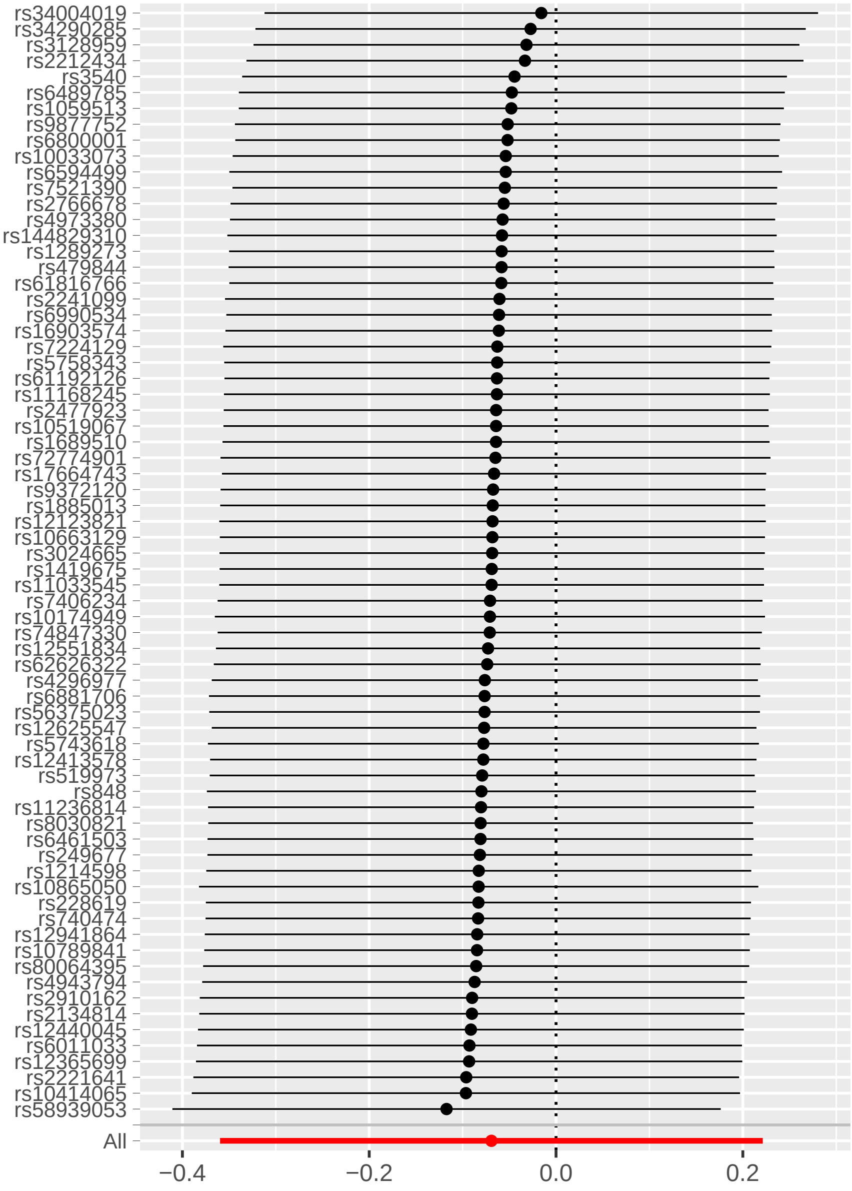

rs34004019
rs34290285
rs3128959
rs2212434
rs3540
rs6489785
rs1059513
rs9877752
rs6800001
rs10033073
rs6594499
rs7521390
rs2766678
rs4973380
rs144829310
rs1289273
rs479844
rs61816766
rs2241099
rs6990534
rs16903574
rs7224129
rs5758343
rs61192126
rs11168245
rs2477923
rs10519067
rs1689510
rs72774901
rs17664743
rs9372120
rs1885013
rs12123821
rs10663129
rs3024665
rs1419675
rs11033545
rs7406234
rs10174949
rs74847330
rs12551834
rs62626322
rs4296977
rs6881706
rs56375023
rs12625547
rs5743618
rs12413578
rs519973
rs848
rs11236814
rs8030821
rs6461503
rs249677
rs1214598
rs10865050
rs228619
rs740474
rs12941864
rs10789841
rs80064395
rs4943794
rs2910162
rs2134814
rs12440045
rs6011033
rs12365699
rs2221641
rs10414065
rs58939053
All
−0.4
−0.2
0.0
0.2

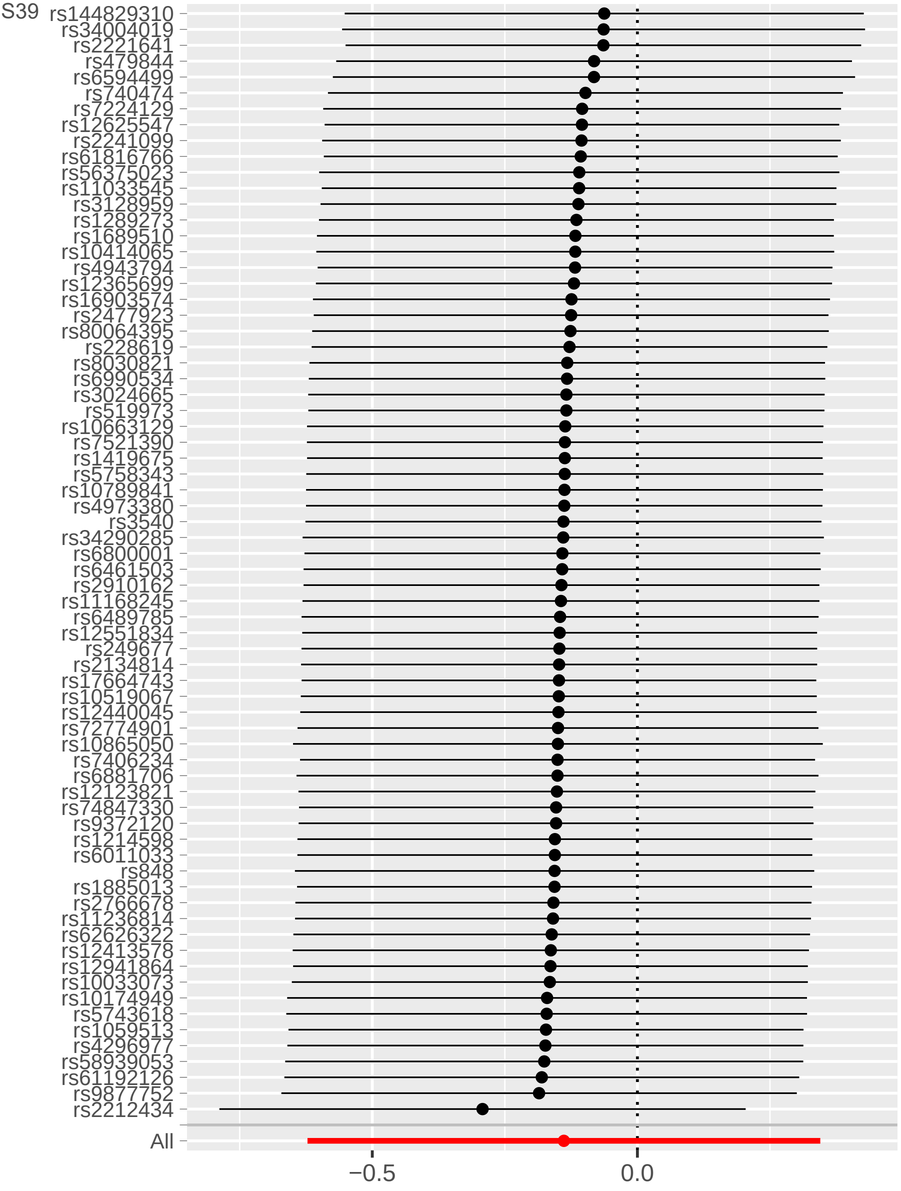
S39
rs144829310
rs34004019
rs2221641
rs479844
rs6594499
rs740474
rs7224129
rs12625547
rs2241099
rs61816766
rs56375023
rs11033545
rs3128959
rs1289273
rs1689510
rs10414065
rs4943794
rs12365699
rs16903574
rs2477923
rs80064395
rs228619
rs8030821
rs6990534
rs3024665
rs519973
rs10663129
rs7521390
rs1419675
rs5758343
rs10789841
rs4973380
rs3540
rs34290285
rs6800001
rs6461503
rs2910162
rs11168245
rs6489785
rs12551834
rs249677
rs2134814
rs17664743
rs10519067
rs12440045
rs72774901
rs10865050
rs7406234
rs6881706
rs12123821
rs74847330
rs9372120
rs1214598
rs6011033
rs848
rs1885013
rs2766678
rs11236814
rs62626322
rs12413578
rs12941864
rs10033073
rs10174949
rs5743618
rs1059513
rs4296977
rs58939053
rs61192126
rs9877752
rs2212434
All
−0.5
0.0

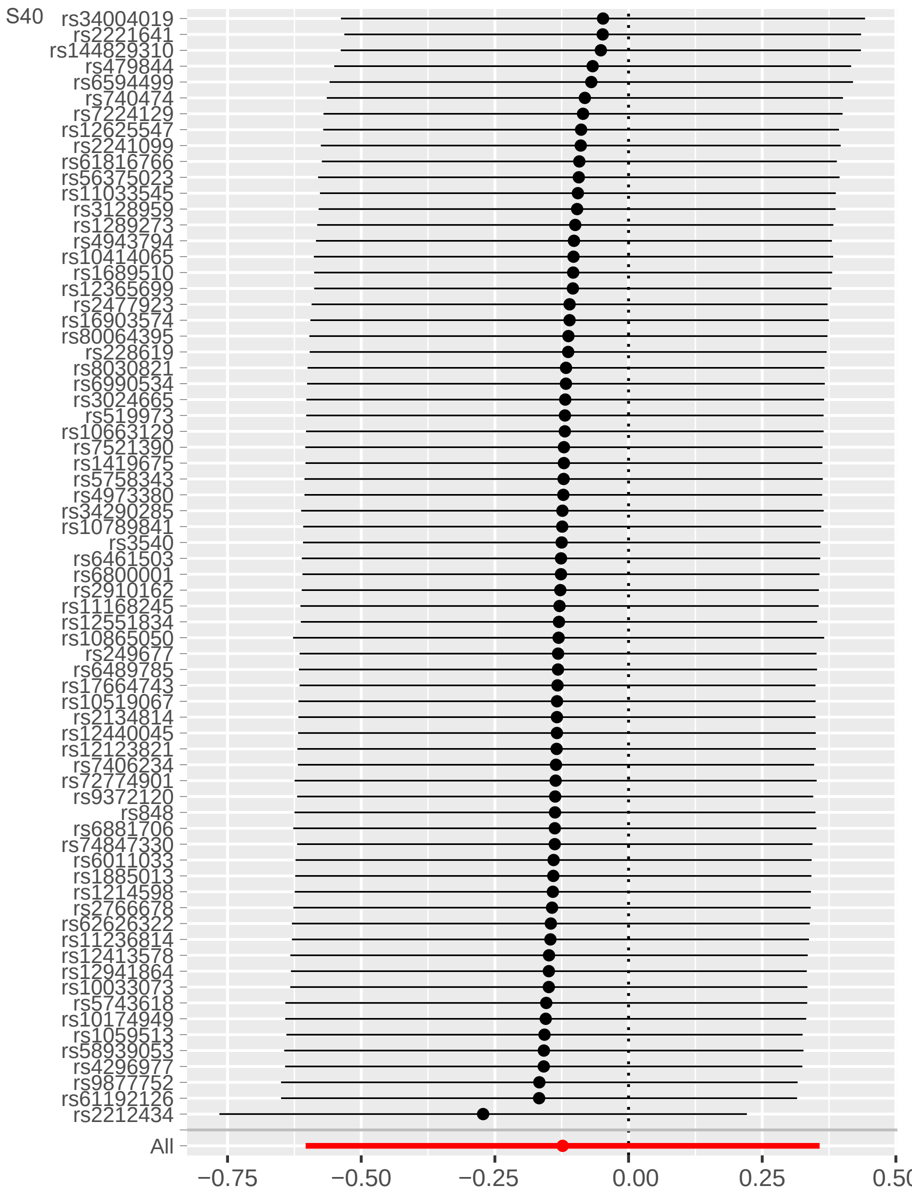
S40
rs34004019
rs2221641
rs144829310
rs479844
rs6594499
rs740474
rs7224129
rs12625547
rs2241099
rs61816766
rs56375023
rs11033545
rs3128959
rs1289273
rs4943794
rs10414065
rs1689510
rs12365699
rs2477923
rs16903574
rs80064395
rs228619
rs8030821
rs6990534
rs3024665
rs519973
rs10663129
rs7521390
rs1419675
rs5758343
rs4973380
rs34290285
rs10789841
rs3540
rs6461503
rs6800001
rs2910162
rs11168245
rs12551834
rs10865050
rs249677
rs6489785
rs17664743
rs10519067
rs2134814
rs12440045
rs12123821
rs7406234
rs72774901
rs9372120
rs848
rs6881706
rs74847330
rs6011033
rs1885013
rs1214598
rs2766678
rs62626322
rs11236814
rs12413578
rs12941864
rs10033073
rs5743618
rs10174949
rs1059513
rs58939053
rs4296977
rs9877752
rs61192126
rs2212434
All
−0.75
−0.50
−0.25
0.00
0.25
0.50

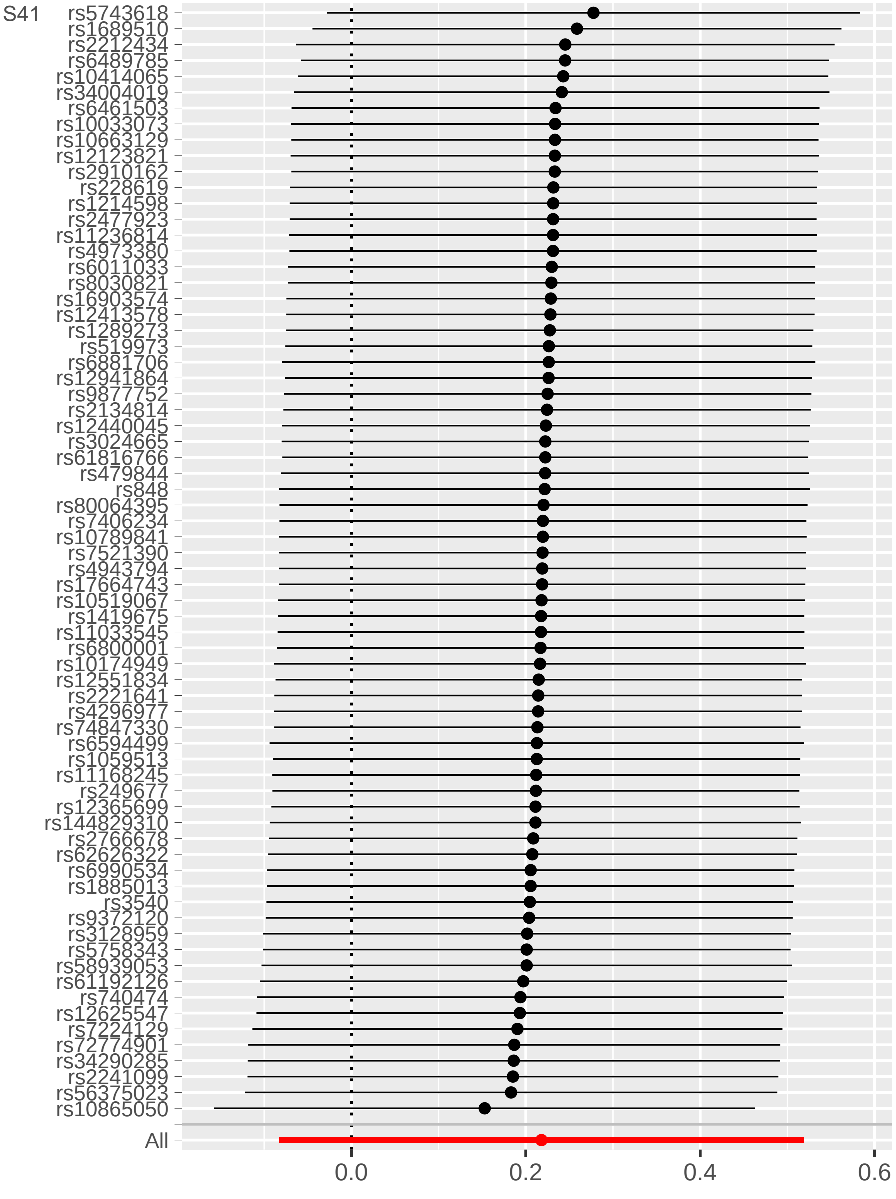

S41
rs5743618
rs1689510
rs2212434
rs6489785
rs10414065
rs34004019
rs6461503
rs10033073
rs10663129
rs12123821
rs2910162
rs228619
rs1214598
rs2477923
rs11236814
rs4973380
rs6011033
rs8030821
rs16903574
rs12413578
rs1289273
rs519973
rs6881706
rs12941864
rs9877752
rs2134814
rs12440045
rs3024665
rs61816766
rs479844
rs848
rs80064395
rs7406234
rs10789841
rs7521390
rs4943794
rs17664743
rs10519067
rs1419675
rs11033545
rs6800001
rs10174949
rs12551834
rs2221641
rs4296977
rs74847330
rs6594499
rs1059513
rs11168245
rs249677
rs12365699
rs144829310
rs2766678
rs62626322
rs6990534
rs1885013
rs3540
rs9372120
rs3128959
rs5758343
rs58939053
rs61192126
rs740474
rs12625547
rs7224129
rs72774901
rs34290285
rs2241099
rs56375023
rs10865050
All
0.0
0.2
0.4
0.6

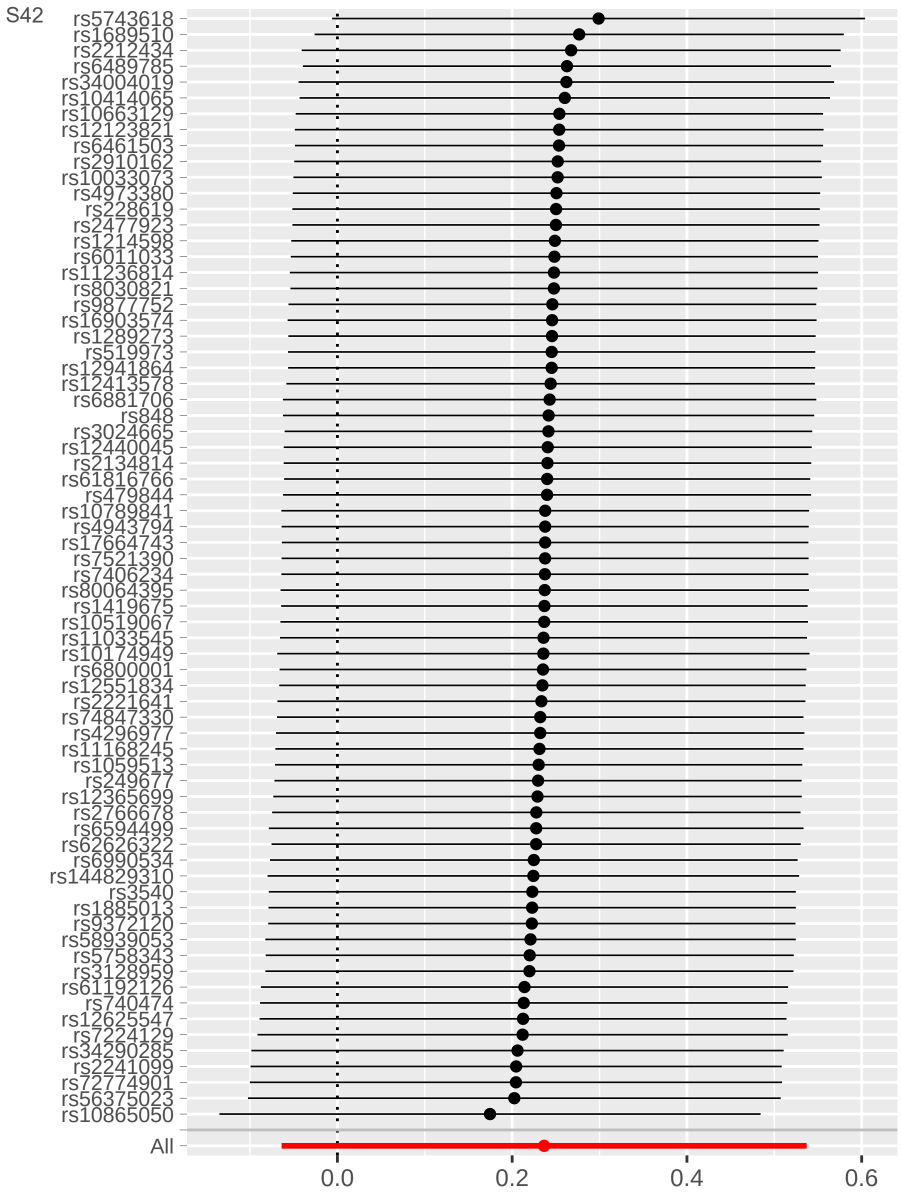

S42
rs5743618
rs1689510
rs2212434
rs6489785
rs34004019
rs10414065
rs10663129
rs12123821
rs6461503
rs2910162
rs10033073
rs4973380
rs228619
rs2477923
rs1214598
rs6011033
rs11236814
rs8030821
rs9877752
rs16903574
rs1289273
rs519973
rs12941864
rs12413578
rs6881706
rs848
rs3024665
rs12440045
rs2134814
rs61816766
rs479844
rs10789841
rs4943794
rs17664743
rs7521390
rs7406234
rs80064395
rs1419675
rs10519067
rs11033545
rs10174949
rs6800001
rs12551834
rs2221641
rs74847330
rs4296977
rs11168245
rs1059513
rs249677
rs12365699
rs2766678
rs6594499
rs62626322
rs6990534
rs144829310
rs3540
rs1885013
rs9372120
rs58939053
rs5758343
rs3128959
rs61192126
rs740474
rs12625547
rs7224129
rs34290285
rs2241099
rs72774901
rs56375023
rs10865050
All
0.0
0.2
0.4
0.6

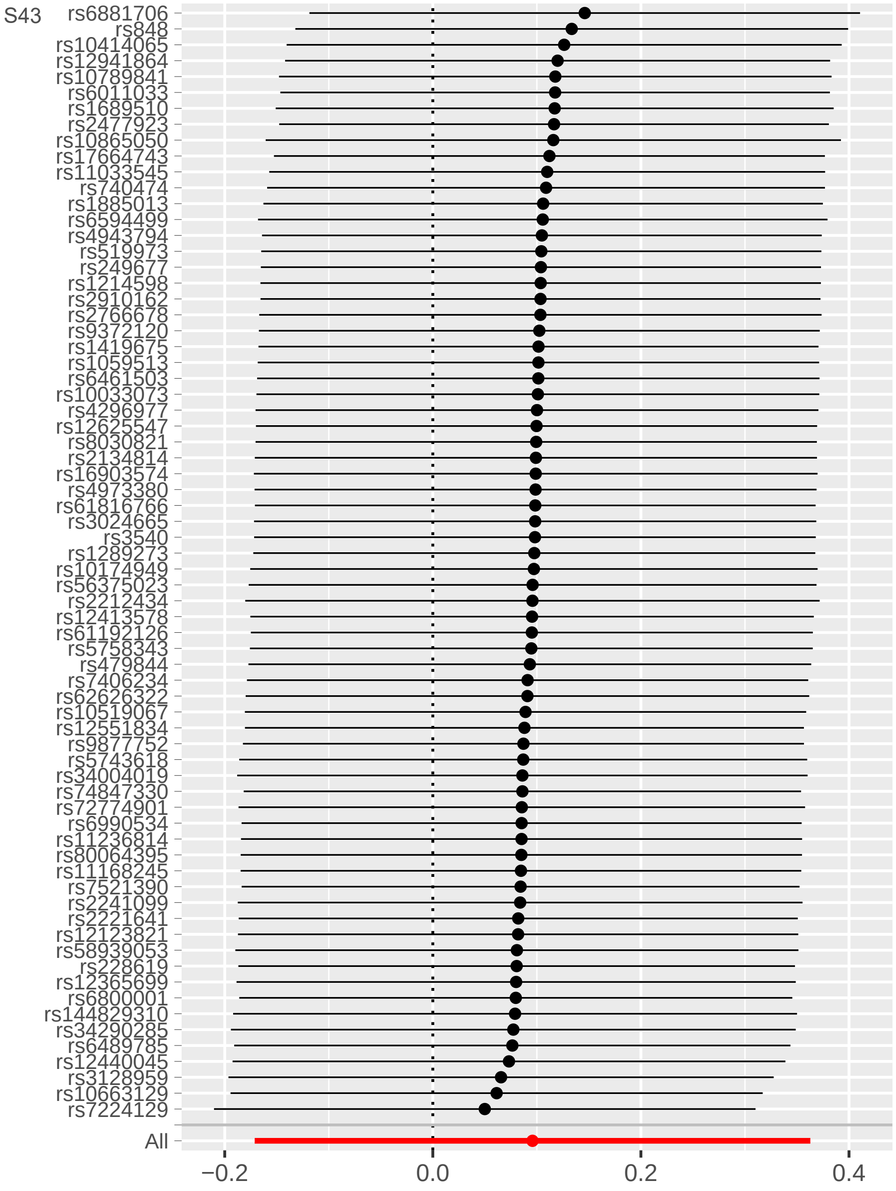

S43
rs6881706
rs848
rs10414065
rs12941864
rs10789841
rs6011033
rs1689510
rs2477923
rs10865050
rs17664743
rs11033545
rs740474
rs1885013
rs6594499
rs4943794
rs519973
rs249677
rs1214598
rs2910162
rs2766678
rs9372120
rs1419675
rs1059513
rs6461503
rs10033073
rs4296977
rs12625547
rs8030821
rs2134814
rs16903574
rs4973380
rs61816766
rs3024665
rs3540
rs1289273
rs10174949
rs56375023
rs2212434
rs12413578
rs61192126
rs5758343
rs479844
rs7406234
rs62626322
rs10519067
rs12551834
rs9877752
rs5743618
rs34004019
rs74847330
rs72774901
rs6990534
rs11236814
rs80064395
rs11168245
rs7521390
rs2241099
rs2221641
rs12123821
rs58939053
rs228619
rs12365699
rs6800001
rs144829310
rs34290285
rs6489785
rs12440045
rs3128959
rs10663129
rs7224129
All
−0.2
0.0
0.2
0.4

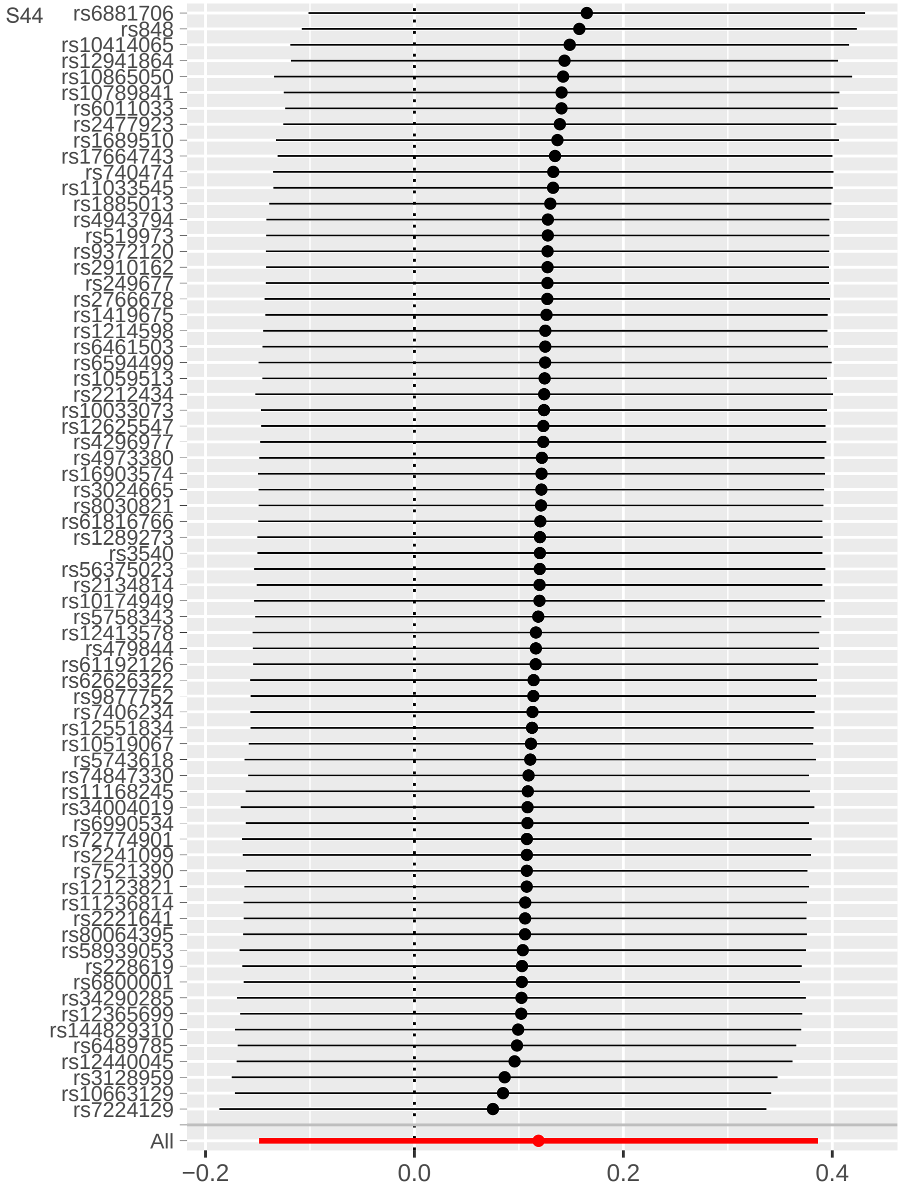
S44
rs6881706
rs848
rs10414065
rs12941864
rs10865050
rs10789841
rs6011033
rs2477923
rs1689510
rs17664743
rs740474
rs11033545
rs1885013
rs4943794
rs519973
rs9372120
rs2910162
rs249677
rs2766678
rs1419675
rs1214598
rs6461503
rs6594499
rs1059513
rs2212434
rs10033073
rs12625547
rs4296977
rs4973380
rs16903574
rs3024665
rs8030821
rs61816766
rs1289273
rs3540
rs56375023
rs2134814
rs10174949
rs5758343
rs12413578
rs479844
rs61192126
rs62626322
rs9877752
rs7406234
rs12551834
rs10519067
rs5743618
rs74847330
rs11168245
rs34004019
rs6990534
rs72774901
rs2241099
rs7521390
rs12123821
rs11236814
rs2221641
rs80064395
rs58939053
rs228619
rs6800001
rs34290285
rs12365699
rs144829310
rs6489785
rs12440045
rs3128959
rs10663129
rs7224129
All
−0.2
0.0
0.2
0.4

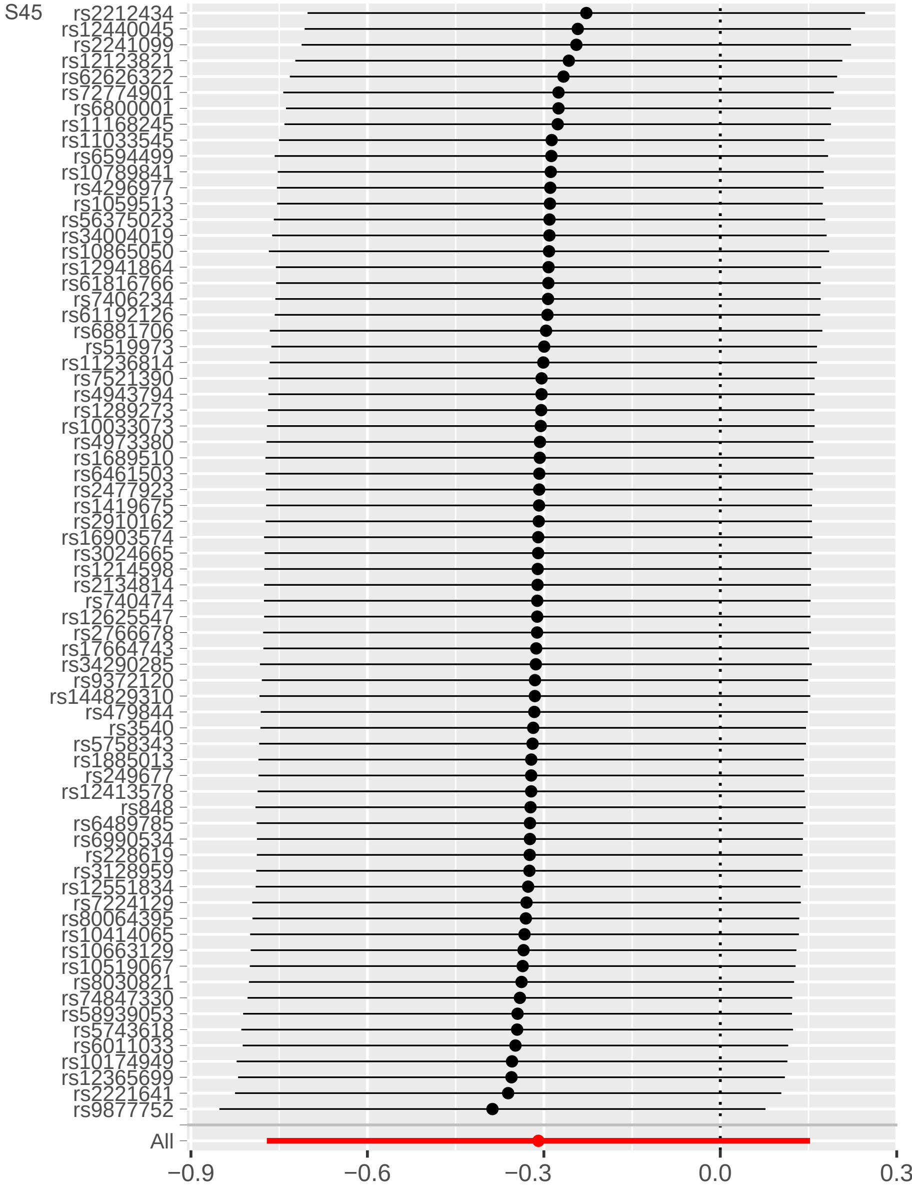

S45
rs2212434
rs12440045
rs2241099
rs12123821
rs62626322
rs72774901
rs6800001
rs11168245
rs11033545
rs6594499
rs10789841
rs4296977
rs1059513
rs56375023
rs34004019
rs10865050
rs12941864
rs61816766
rs7406234
rs61192126
rs6881706
rs519973
rs11236814
rs7521390
rs4943794
rs1289273
rs10033073
rs4973380
rs1689510
rs6461503
rs2477923
rs1419675
rs2910162
rs16903574
rs3024665
rs1214598
rs2134814
rs740474
rs12625547
rs2766678
rs17664743
rs34290285
rs9372120
rs144829310
rs479844
rs3540
rs5758343
rs1885013
rs249677
rs12413578
rs848
rs6489785
rs6990534
rs228619
rs3128959
rs12551834
rs7224129
rs80064395
rs10414065
rs10663129
rs10519067
rs8030821
rs74847330
rs58939053
rs5743618
rs6011033
rs10174949
rs12365699
rs2221641
rs9877752
All
−0.9
−0.6
−0.3
0.0
0.3

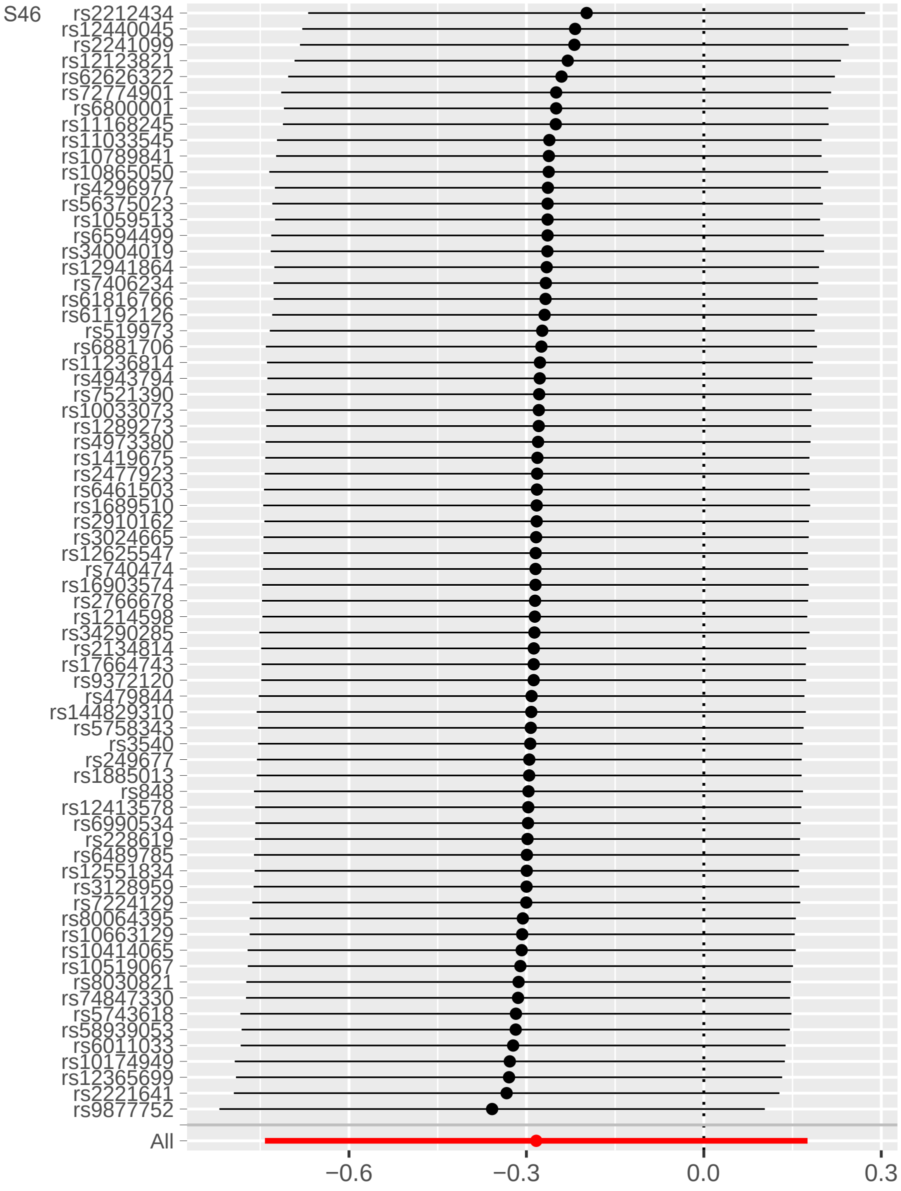
S46
rs2212434
rs12440045
rs2241099
rs12123821
rs62626322
rs72774901
rs6800001
rs11168245
rs11033545
rs10789841
rs10865050
rs4296977
rs56375023
rs1059513
rs6594499
rs34004019
rs12941864
rs7406234
rs61816766
rs61192126
rs519973
rs6881706
rs11236814
rs4943794
rs7521390
rs10033073
rs1289273
rs4973380
rs1419675
rs2477923
rs6461503
rs1689510
rs2910162
rs3024665
rs12625547
rs740474
rs16903574
rs2766678
rs1214598
rs34290285
rs2134814
rs17664743
rs9372120
rs479844
rs144829310
rs5758343
rs3540
rs249677
rs1885013
rs848
rs12413578
rs6990534
rs228619
rs6489785
rs12551834
rs3128959
rs7224129
rs80064395
rs10663129
rs10414065
rs10519067
rs8030821
rs74847330
rs5743618
rs58939053
rs6011033
rs10174949
rs12365699
rs2221641
rs9877752
All
−0.6
−0.3
0.0
0.3

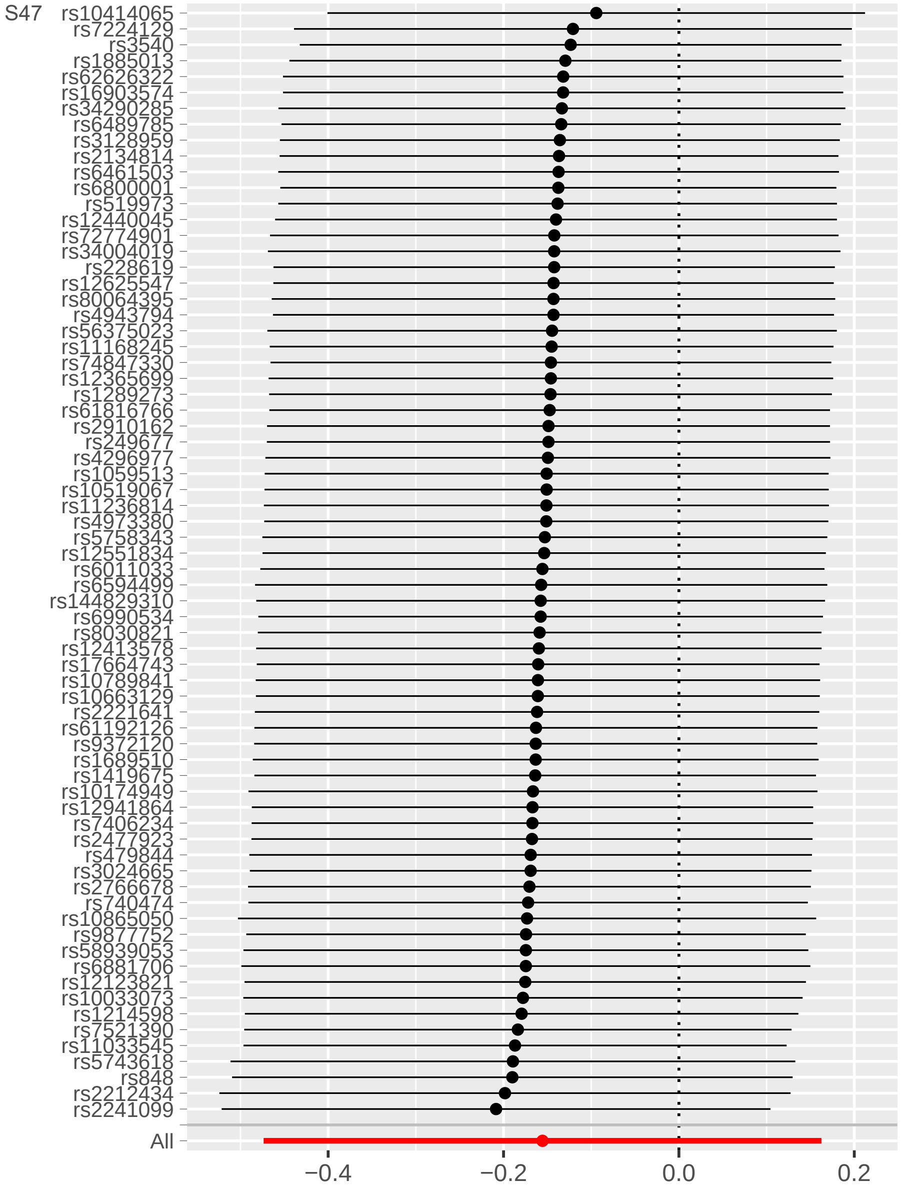

S47
rs10414065
rs7224129
rs3540
rs1885013
rs62626322
rs16903574
rs34290285
rs6489785
rs3128959
rs2134814
rs6461503
rs6800001
rs519973
rs12440045
rs72774901
rs34004019
rs228619
rs12625547
rs80064395
rs4943794
rs56375023
rs11168245
rs74847330
rs12365699
rs1289273
rs61816766
rs2910162
rs249677
rs4296977
rs1059513
rs10519067
rs11236814
rs4973380
rs5758343
rs12551834
rs6011033
rs6594499
rs144829310
rs6990534
rs8030821
rs12413578
rs17664743
rs10789841
rs10663129
rs2221641
rs61192126
rs9372120
rs1689510
rs1419675
rs10174949
rs12941864
rs7406234
rs2477923
rs479844
rs3024665
rs2766678
rs740474
rs10865050
rs9877752
rs58939053
rs6881706
rs12123821
rs10033073
rs1214598
rs7521390
rs11033545
rs5743618
rs848
rs2212434
rs2241099
All
−0.4
−0.2
0.0
0.2

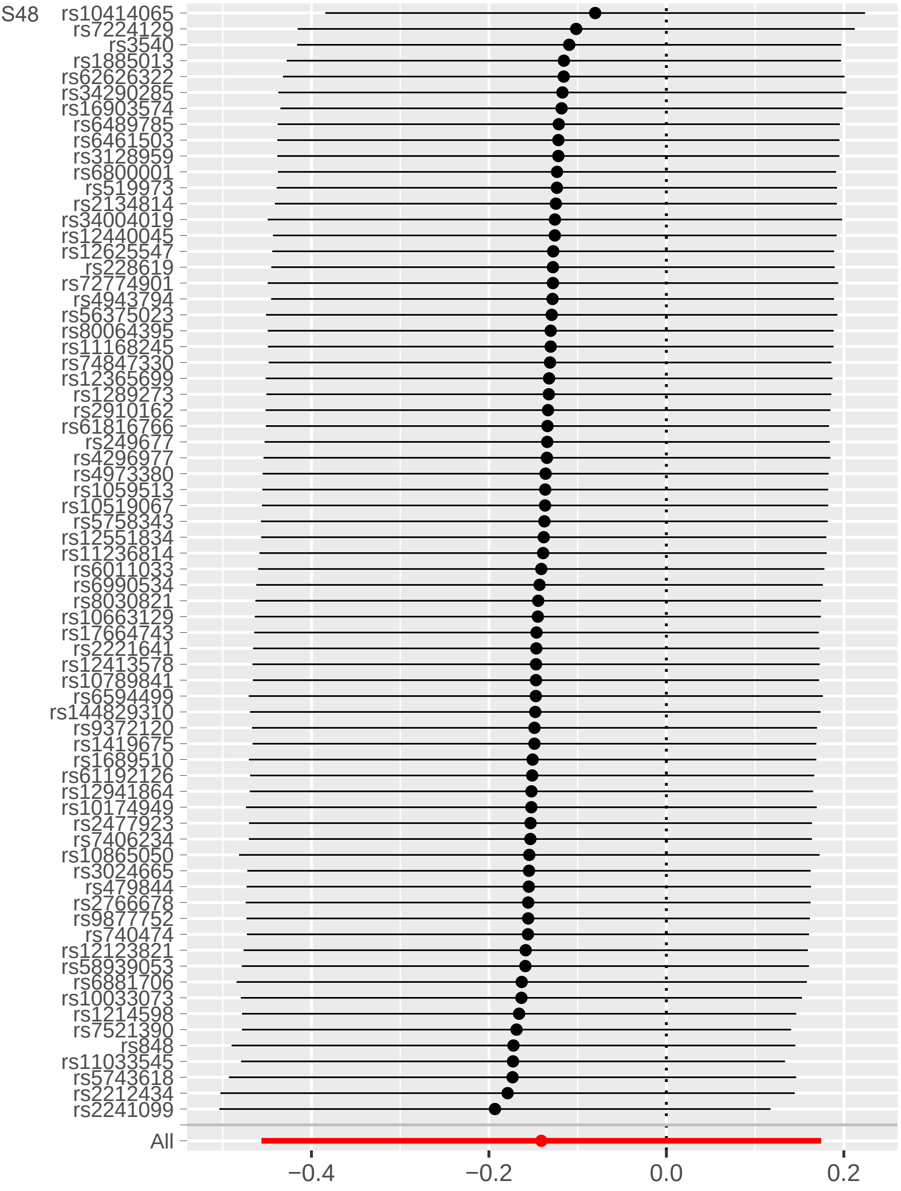
S48
rs10414065
rs7224129
rs3540
rs1885013
rs62626322
rs34290285
rs16903574
rs6489785
rs6461503
rs3128959
rs6800001
rs519973
rs2134814
rs34004019
rs12440045
rs12625547
rs228619
rs72774901
rs4943794
rs56375023
rs80064395
rs11168245
rs74847330
rs12365699
rs1289273
rs2910162
rs61816766
rs249677
rs4296977
rs4973380
rs1059513
rs10519067
rs5758343
rs12551834
rs11236814
rs6011033
rs6990534
rs8030821
rs10663129
rs17664743
rs2221641
rs12413578
rs10789841
rs6594499
rs144829310
rs9372120
rs1419675
rs1689510
rs61192126
rs12941864
rs10174949
rs2477923
rs7406234
rs10865050
rs3024665
rs479844
rs2766678
rs9877752
rs740474
rs12123821
rs58939053
rs6881706
rs10033073
rs1214598
rs7521390
rs848
rs11033545
rs5743618
rs2212434
rs2241099
All
−0.4
−0.2
0.0
0.2

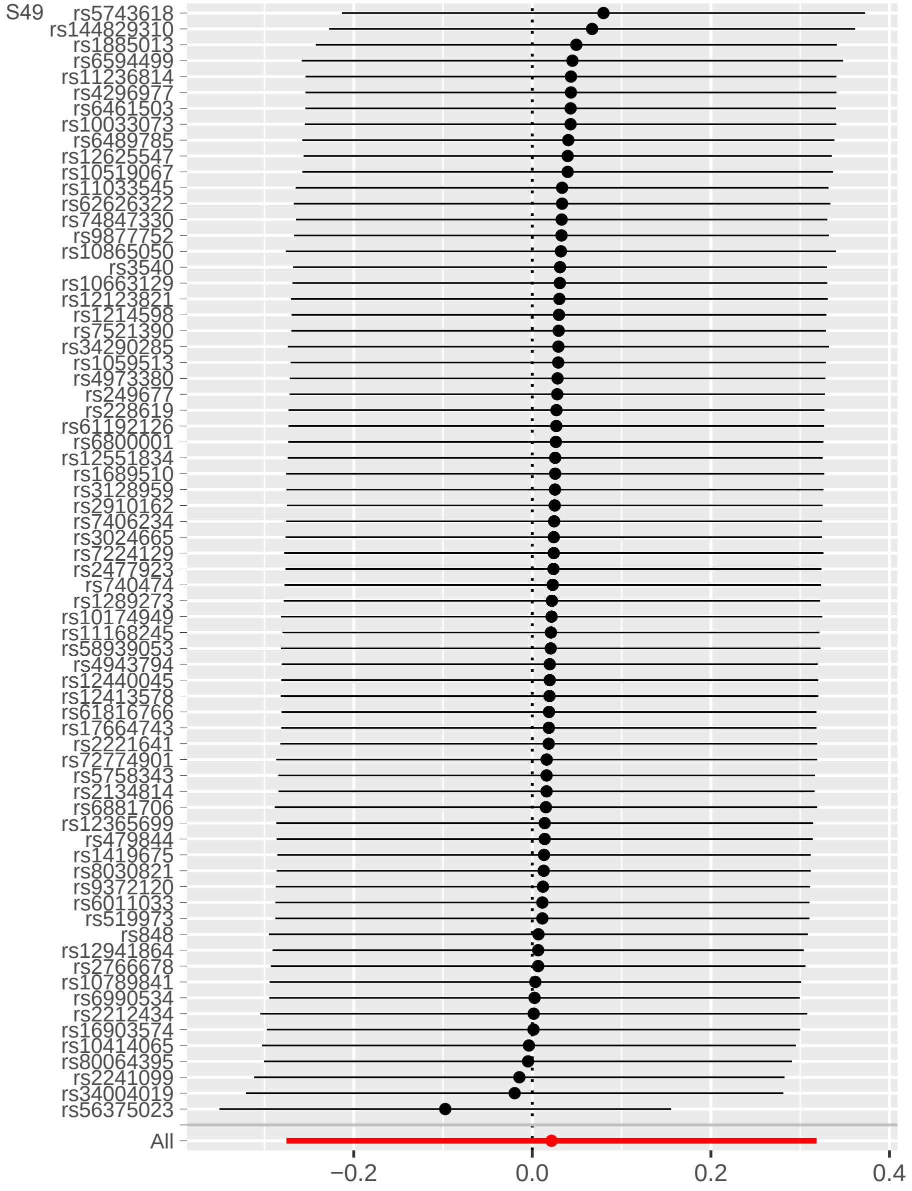
S49
rs5743618
rs144829310
rs1885013
rs6594499
rs11236814
rs4296977
rs6461503
rs10033073
rs6489785
rs12625547
rs10519067
rs11033545
rs62626322
rs74847330
rs9877752
rs10865050
rs3540
rs10663129
rs12123821
rs1214598
rs7521390
rs34290285
rs1059513
rs4973380
rs249677
rs228619
rs61192126
rs6800001
rs12551834
rs1689510
rs3128959
rs2910162
rs7406234
rs3024665
rs7224129
rs2477923
rs740474
rs1289273
rs10174949
rs11168245
rs58939053
rs4943794
rs12440045
rs12413578
rs61816766
rs17664743
rs2221641
rs72774901
rs5758343
rs2134814
rs6881706
rs12365699
rs479844
rs1419675
rs8030821
rs9372120
rs6011033
rs519973
rs848
rs12941864
rs2766678
rs10789841
rs6990534
rs2212434
rs16903574
rs10414065
rs80064395
rs2241099
rs34004019
rs56375023
All
−0.2
0.0
0.2
0.4

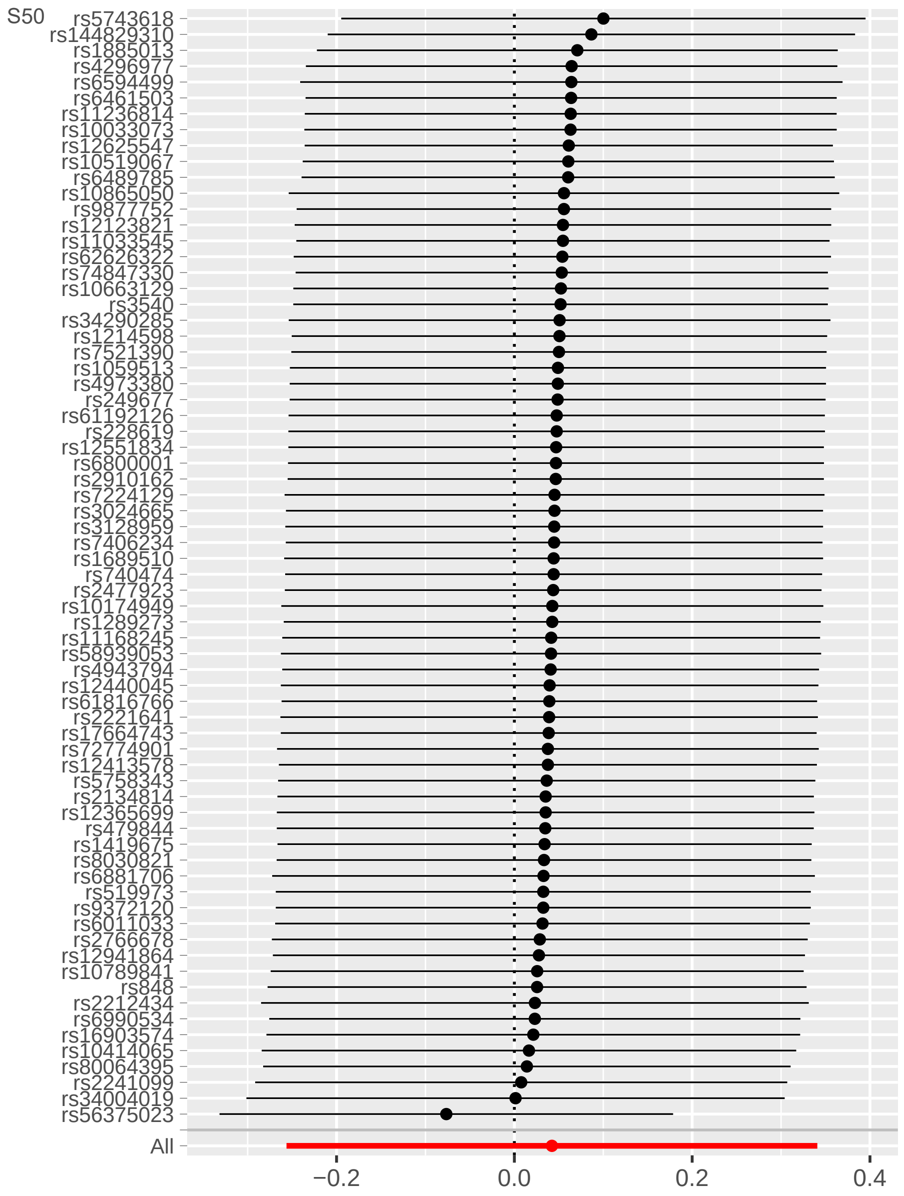

S50
rs5743618
rs144829310
rs1885013
rs4296977
rs6594499
rs6461503
rs11236814
rs10033073
rs12625547
rs10519067
rs6489785
rs10865050
rs9877752
rs12123821
rs11033545
rs62626322
rs74847330
rs10663129
rs3540
rs34290285
rs1214598
rs7521390
rs1059513
rs4973380
rs249677
rs61192126
rs228619
rs12551834
rs6800001
rs2910162
rs7224129
rs3024665
rs3128959
rs7406234
rs1689510
rs740474
rs2477923
rs10174949
rs1289273
rs11168245
rs58939053
rs4943794
rs12440045
rs61816766
rs2221641
rs17664743
rs72774901
rs12413578
rs5758343
rs2134814
rs12365699
rs479844
rs1419675
rs8030821
rs6881706
rs519973
rs9372120
rs6011033
rs2766678
rs12941864
rs10789841
rs848
rs2212434
rs6990534
rs16903574
rs10414065
rs80064395
rs2241099
rs34004019
rs56375023
All
−0.2
0.0
0.2
0.4

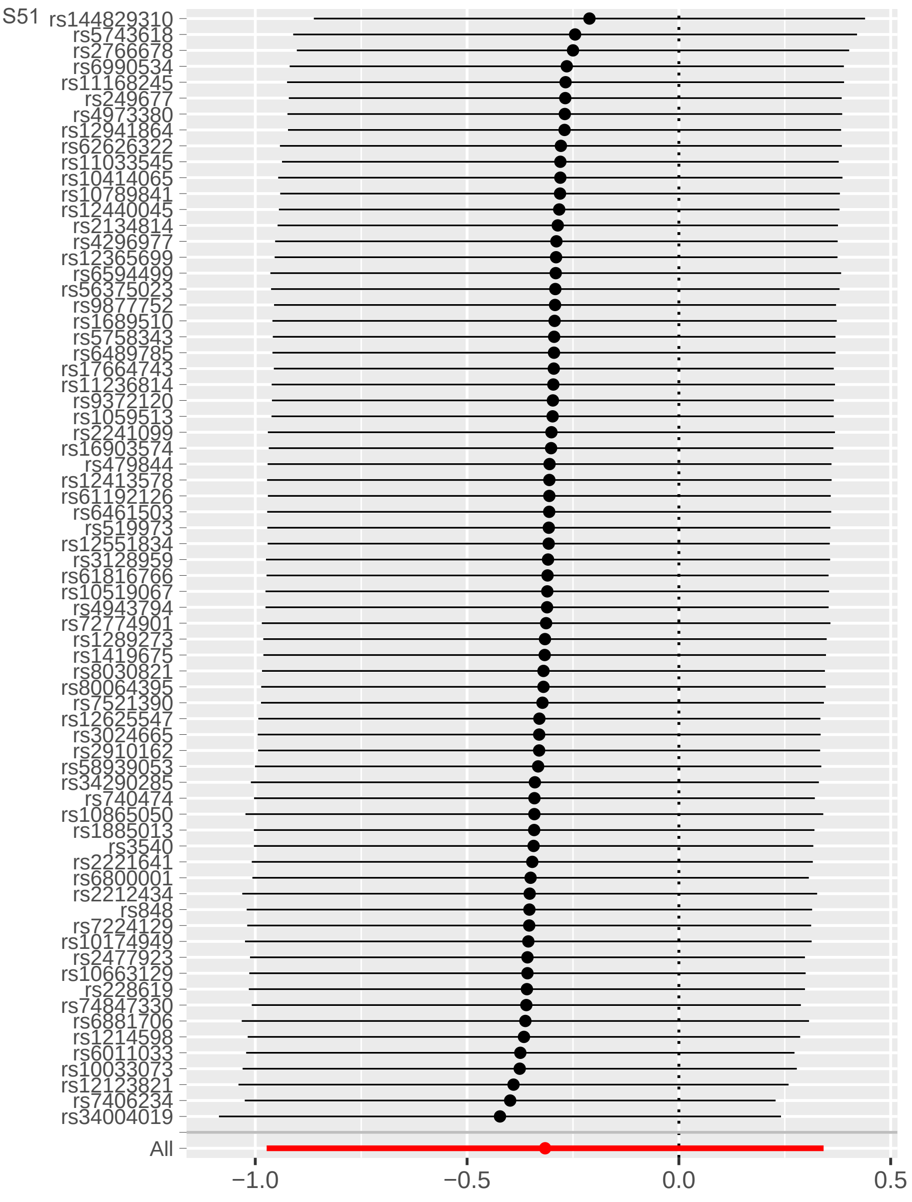
S51
rs144829310
rs5743618
rs2766678
rs6990534
rs11168245
rs249677
rs4973380
rs12941864
rs62626322
rs11033545
rs10414065
rs10789841
rs12440045
rs2134814
rs4296977
rs12365699
rs6594499
rs56375023
rs9877752
rs1689510
rs5758343
rs6489785
rs17664743
rs11236814
rs9372120
rs1059513
rs2241099
rs16903574
rs479844
rs12413578
rs61192126
rs6461503
rs519973
rs12551834
rs3128959
rs61816766
rs10519067
rs4943794
rs72774901
rs1289273
rs1419675
rs8030821
rs80064395
rs7521390
rs12625547
rs3024665
rs2910162
rs58939053
rs34290285
rs740474
rs10865050
rs1885013
rs3540
rs2221641
rs6800001
rs2212434
rs848
rs7224129
rs10174949
rs2477923
rs10663129
rs228619
rs74847330
rs6881706
rs1214598
rs6011033
rs10033073
rs12123821
rs7406234
rs34004019
All
−1.0
−0.5
0.0
0.5

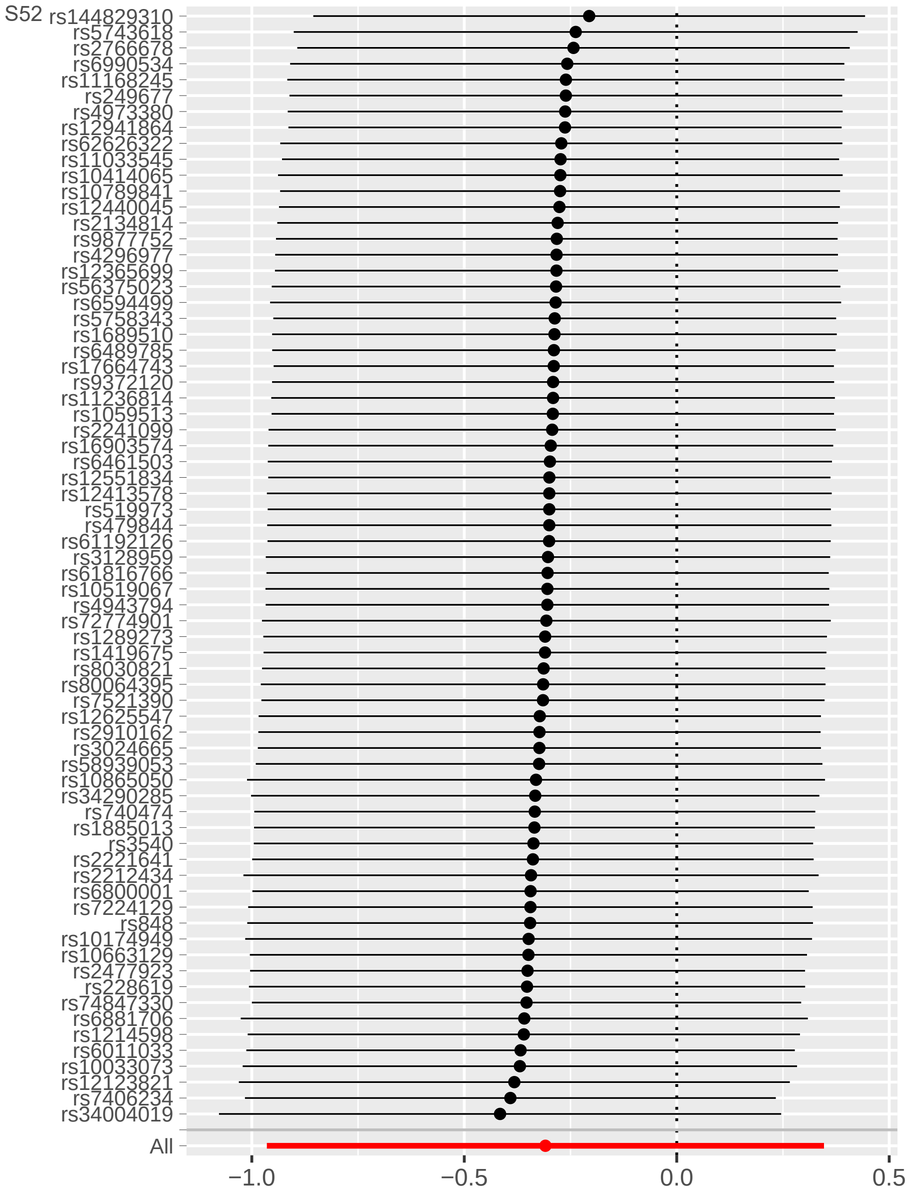
S52
rs144829310
rs5743618
rs2766678
rs6990534
rs11168245
rs249677
rs4973380
rs12941864
rs62626322
rs11033545
rs10414065
rs10789841
rs12440045
rs2134814
rs9877752
rs4296977
rs12365699
rs56375023
rs6594499
rs5758343
rs1689510
rs6489785
rs17664743
rs9372120
rs11236814
rs1059513
rs2241099
rs16903574
rs6461503
rs12551834
rs12413578
rs519973
rs479844
rs61192126
rs3128959
rs61816766
rs10519067
rs4943794
rs72774901
rs1289273
rs1419675
rs8030821
rs80064395
rs7521390
rs12625547
rs2910162
rs3024665
rs58939053
rs10865050
rs34290285
rs740474
rs1885013
rs3540
rs2221641
rs2212434
rs6800001
rs7224129
rs848
rs10174949
rs10663129
rs2477923
rs228619
rs74847330
rs6881706
rs1214598
rs6011033
rs10033073
rs12123821
rs7406234
rs34004019
All
−1.0
−0.5
0.0
0.5

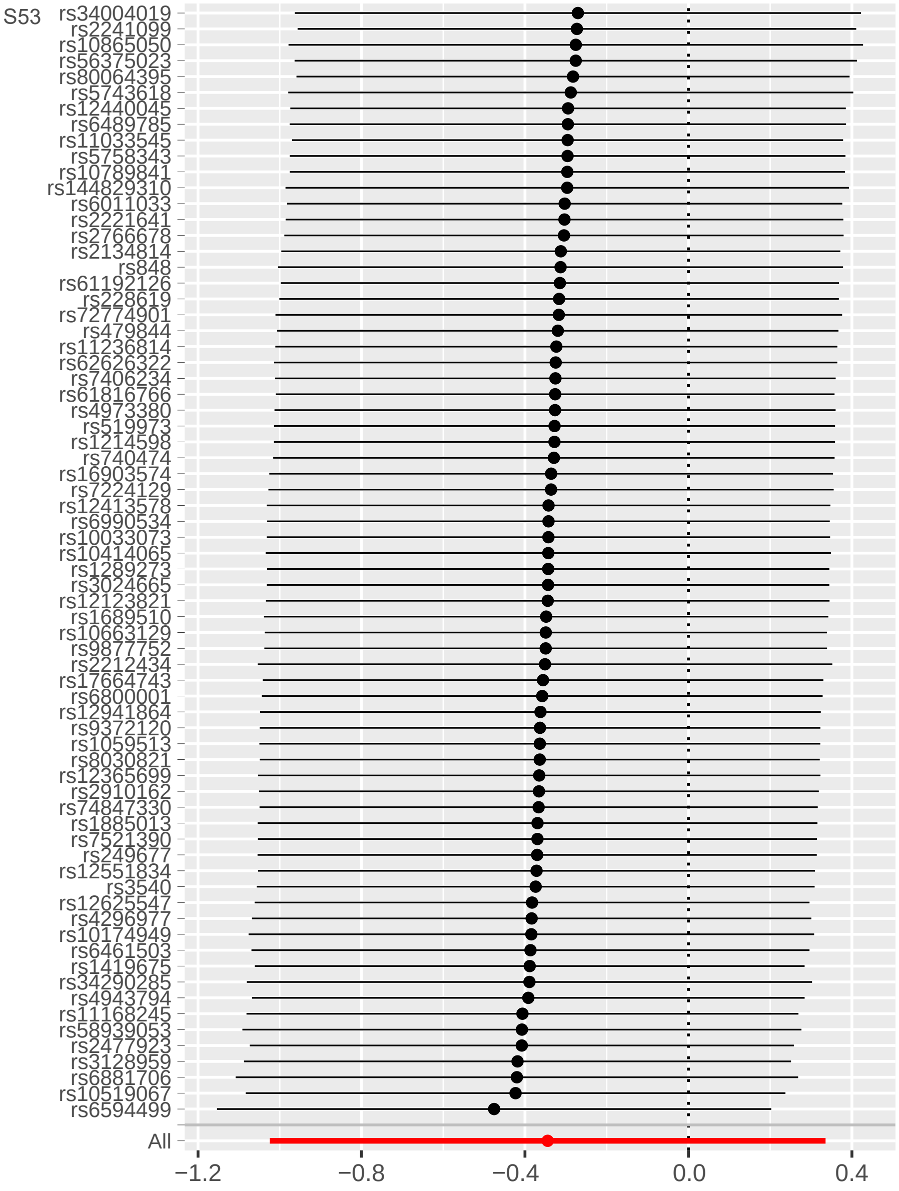

S53
rs34004019
rs2241099
rs10865050
rs56375023
rs80064395
rs5743618
rs12440045
rs6489785
rs11033545
rs5758343
rs10789841
rs144829310
rs6011033
rs2221641
rs2766678
rs2134814
rs848
rs61192126
rs228619
rs72774901
rs479844
rs11236814
rs62626322
rs7406234
rs61816766
rs4973380
rs519973
rs1214598
rs740474
rs16903574
rs7224129
rs12413578
rs6990534
rs10033073
rs10414065
rs1289273
rs3024665
rs12123821
rs1689510
rs10663129
rs9877752
rs2212434
rs17664743
rs6800001
rs12941864
rs9372120
rs1059513
rs8030821
rs12365699
rs2910162
rs74847330
rs1885013
rs7521390
rs249677
rs12551834
rs3540
rs12625547
rs4296977
rs10174949
rs6461503
rs1419675
rs34290285
rs4943794
rs11168245
rs58939053
rs2477923
rs3128959
rs6881706
rs10519067
rs6594499
All
−1.2
−0.8
−0.4
0.0
0.4

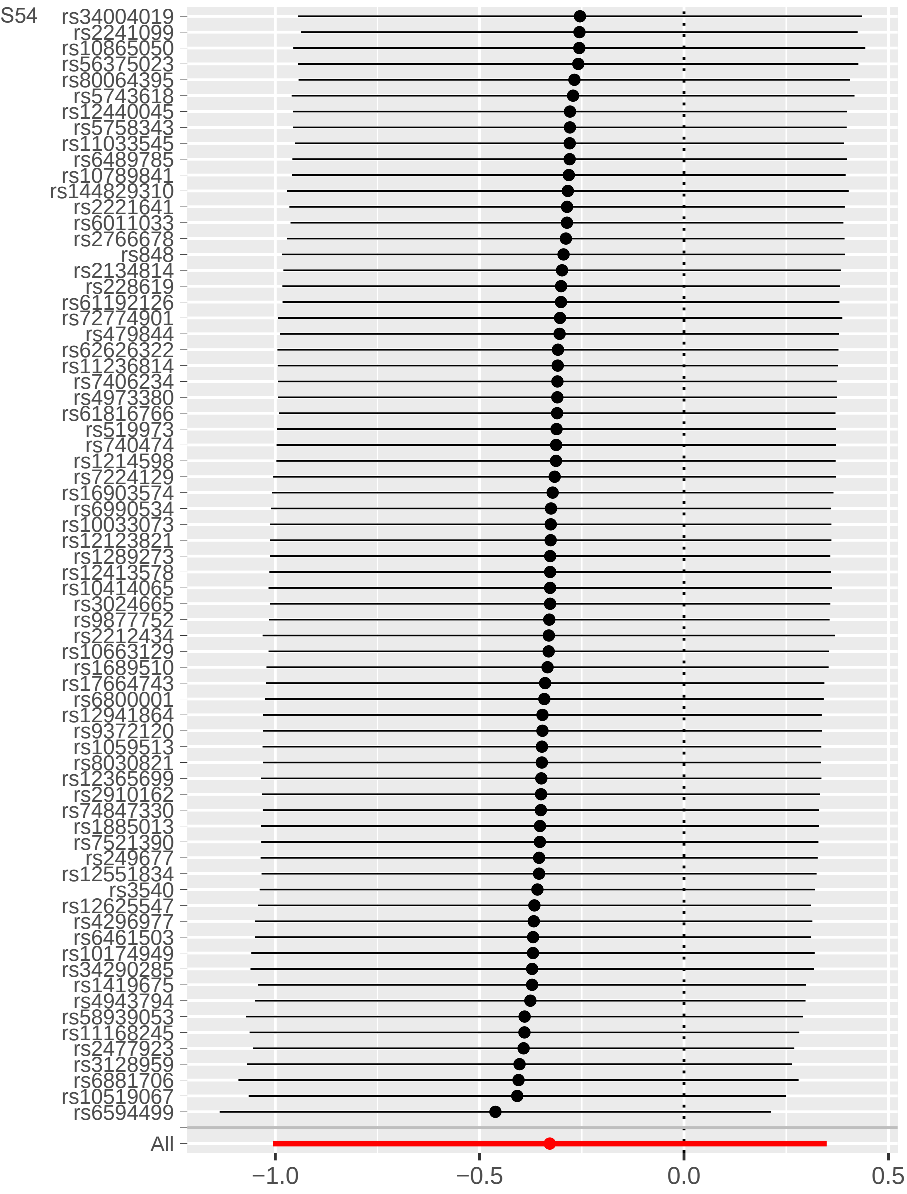
S54
rs34004019
rs2241099
rs10865050
rs56375023
rs80064395
rs5743618
rs12440045
rs5758343
rs11033545
rs6489785
rs10789841
rs144829310
rs2221641
rs6011033
rs2766678
rs848
rs2134814
rs228619
rs61192126
rs72774901
rs479844
rs62626322
rs11236814
rs7406234
rs4973380
rs61816766
rs519973
rs740474
rs1214598
rs7224129
rs16903574
rs6990534
rs10033073
rs12123821
rs1289273
rs12413578
rs10414065
rs3024665
rs9877752
rs2212434
rs10663129
rs1689510
rs17664743
rs6800001
rs12941864
rs9372120
rs1059513
rs8030821
rs12365699
rs2910162
rs74847330
rs1885013
rs7521390
rs249677
rs12551834
rs3540
rs12625547
rs4296977
rs6461503
rs10174949
rs34290285
rs1419675
rs4943794
rs58939053
rs11168245
rs2477923
rs3128959
rs6881706
rs10519067
rs6594499
All
−1.0
−0.5
0.0
0.5

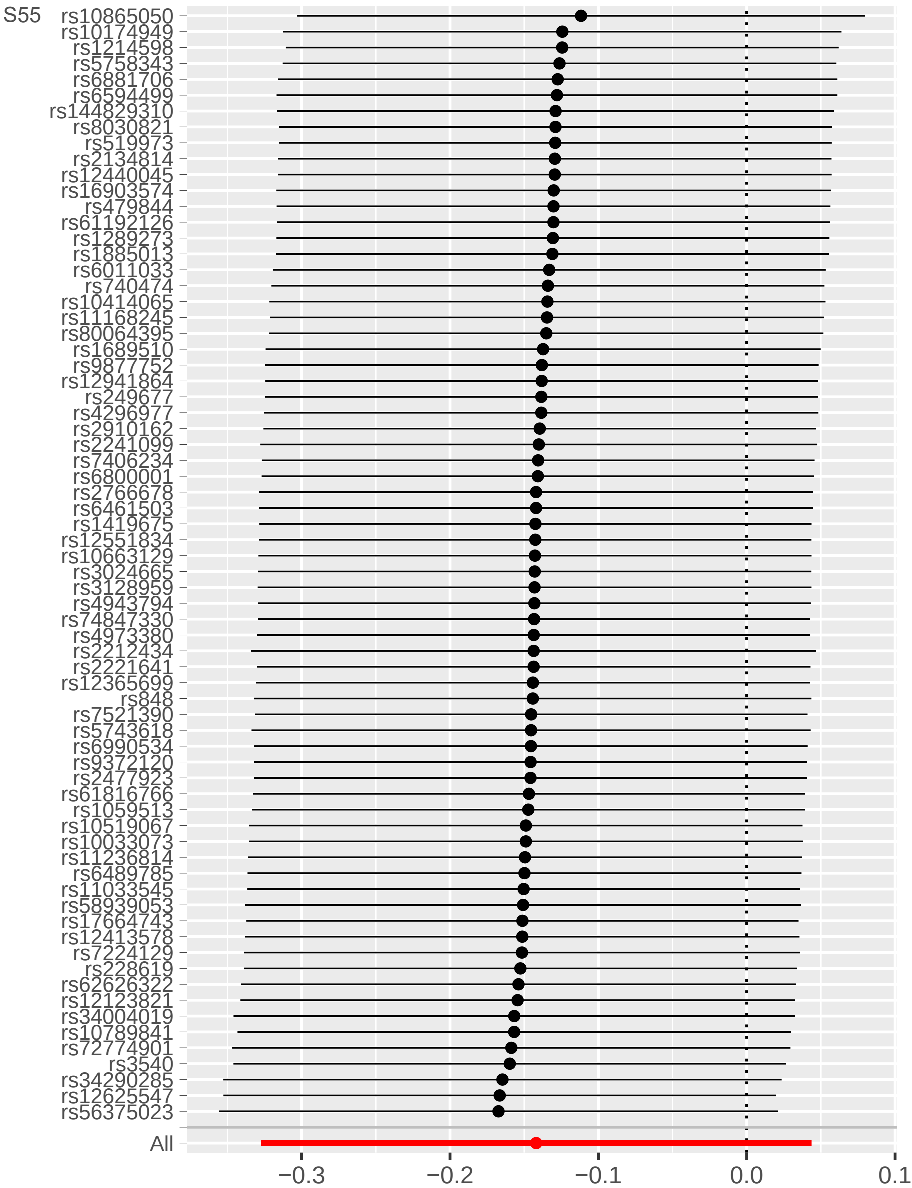
S55
rs10865050
rs10174949
rs1214598
rs5758343
rs6881706
rs6594499
rs144829310
rs8030821
rs519973
rs2134814
rs12440045
rs16903574
rs479844
rs61192126
rs1289273
rs1885013
rs6011033
rs740474
rs10414065
rs11168245
rs80064395
rs1689510
rs9877752
rs12941864
rs249677
rs4296977
rs2910162
rs2241099
rs7406234
rs6800001
rs2766678
rs6461503
rs1419675
rs12551834
rs10663129
rs3024665
rs3128959
rs4943794
rs74847330
rs4973380
rs2212434
rs2221641
rs12365699
rs848
rs7521390
rs5743618
rs6990534
rs9372120
rs2477923
rs61816766
rs1059513
rs10519067
rs10033073
rs11236814
rs6489785
rs11033545
rs58939053
rs17664743
rs12413578
rs7224129
rs228619
rs62626322
rs12123821
rs34004019
rs10789841
rs72774901
rs3540
rs34290285
rs12625547
rs56375023
All
−0.3
−0.2
−0.1
0.0
0.1

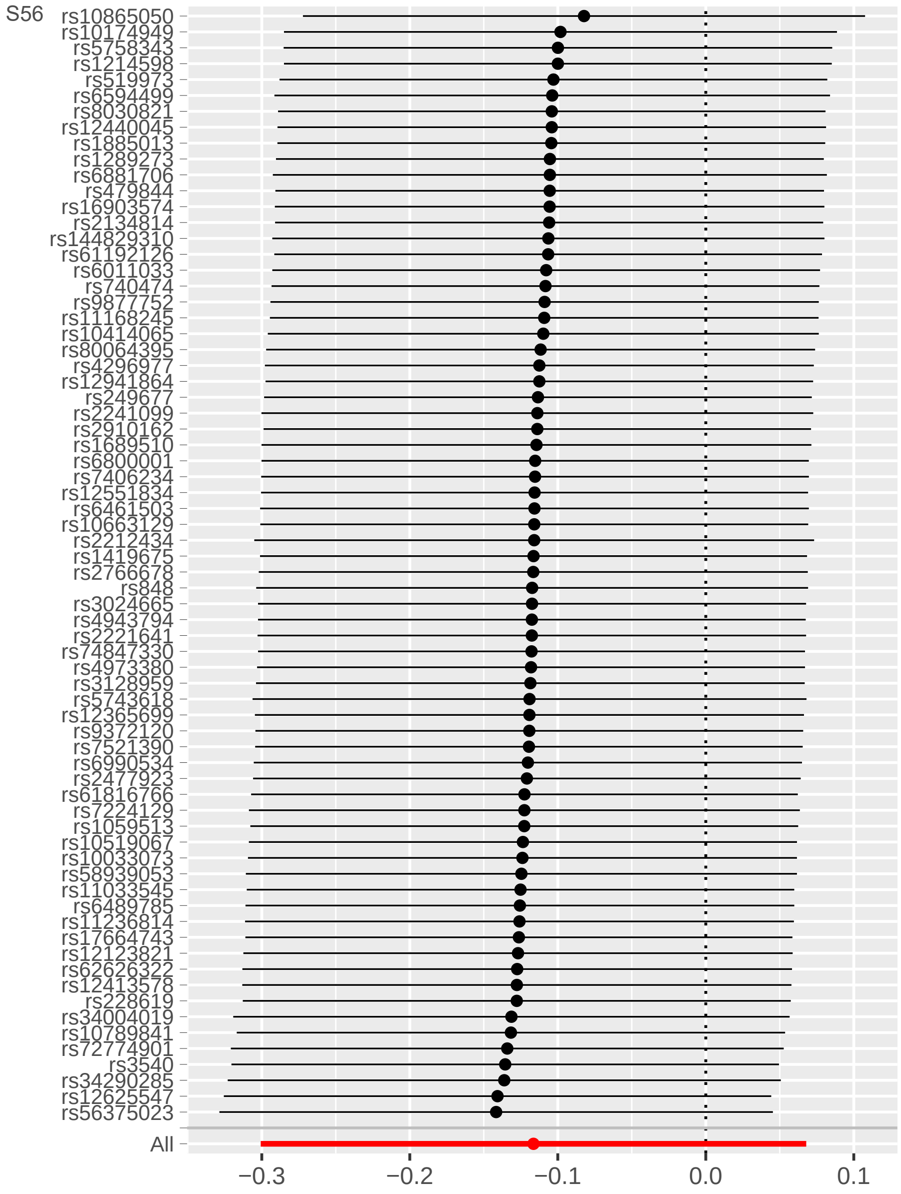

S56
rs10865050
rs10174949
rs5758343
rs1214598
rs519973
rs6594499
rs8030821
rs12440045
rs1885013
rs1289273
rs6881706
rs479844
rs16903574
rs2134814
rs144829310
rs61192126
rs6011033
rs740474
rs9877752
rs11168245
rs10414065
rs80064395
rs4296977
rs12941864
rs249677
rs2241099
rs2910162
rs1689510
rs6800001
rs7406234
rs12551834
rs6461503
rs10663129
rs2212434
rs1419675
rs2766678
rs848
rs3024665
rs4943794
rs2221641
rs74847330
rs4973380
rs3128959
rs5743618
rs12365699
rs9372120
rs7521390
rs6990534
rs2477923
rs61816766
rs7224129
rs1059513
rs10519067
rs10033073
rs58939053
rs11033545
rs6489785
rs11236814
rs17664743
rs12123821
rs62626322
rs12413578
rs228619
rs34004019
rs10789841
rs72774901
rs3540
rs34290285
rs12625547
rs56375023
All
−0.3
−0.2
−0.1
0.0
0.1
